# Effect of intravenous clarithromycin in patients with sepsis, respiratory and multiple organ dysfunction syndrome: a randomized clinical trial

**DOI:** 10.1186/s13054-022-04055-4

**Published:** 2022-06-18

**Authors:** Eleni Karakike, Brendon P. Scicluna, Maria Roumpoutsou, Ioannis Mitrou, Niki Karampela, Athanasios Karageorgos, Konstantinos Psaroulis, Eleni Massa, Achillefs Pitsoulis, Panagiotis Chaloulis, Evanthia Pappa, Irene T. Schrijver, Frantzeska Frantzeskaki, Malvina Lada, Nicolas Dauby, David De Bels, Ioannis Floros, Souzana Anisoglou, Eleni Antoniadou, Maria Patrani, Glykeria Vlachogianni, Eleni Mouloudi, Anastasia Antoniadou, David Grimaldi, Thierry Roger, W. Joost Wiersinga, Iraklis Tsangaris, Evangelos J. Giamarellos-Bourboulis

**Affiliations:** 1 4th Department of Internal Medicine, National and Kapodistrian University of Athens, 1 Rimini Street, 124 62 Athens, Greece; 2 Division of Infectious Diseases, Center for Experimental Molecular Medicine, Amsterdam University Medical Centers, Academic Medical Center, University of Amsterdam, Amsterdam, The Netherlands; 3 Department of Applied Biomedical Science, Faculty of Health Sciences, Mater Dei Hospital, University of Malta, Msida, Malta; 4 Centre for Molecular Medicine and Biobanking, University of Malta, Msida, Malta; 5 Intensive Care Unit, Korgialeneio Benakeio General Hospital, Athens, Greece; 6 Intensive Care Unit, Aghios Dimitrios General Hospital, Thessaloniki, Greece; 7 Intensive Care Unit, Hippokration General Hospital, Athens, Greece; 8 Intensive Care Unit, G. Gennimatas General Hospital, Thessaloniki, Greece; 9 Intensive Care Unit, Theageneion General Hospital, Thessaloniki, Greece; 10 Intensive Care Unit, Laiko General Hospital, Athens, Greece; 11 Infectious Diseases Service, Department of Medicine, Lausanne University Hospital and University of Lausanne, Lausanne, Switzerland; 12 2nd Department of Critical Care Medicine, National and Kapodistrian University of Athens, Athens, Greece; 13 2nd Department of Internal Medicine, Sismanogleion General Hospital, Athens, Greece; 14 Department of Infectious Diseases, Centre Hospitalier Universitaire Saint-Pierre, Université Libre de Bruxelles (ULB), Brussels, Belgium; 15 Institute for Medical Immunology, Université Libre de Bruxelles (ULB), Brussels, Belgium; 16 Department of Intensive Care, Centre Hospitalier Universitaire Brugmann, Brussels, Belgium; 17 Department of Intensive Care, CUB-Erasme, Université Libre de Bruxelles (ULB), Brussels, Belgium

**Keywords:** Macrolides, Clarithromycin, Sepsis, Multiple organ dysfunction, Recurrence, Cholesterol

## Abstract

**Background:**

Clarithromycin may act as immune-regulating treatment in sepsis and acute respiratory dysfunction syndrome. However, clinical evidence remains inconclusive. We aimed to evaluate whether clarithromycin improves 28-day mortality among patients with sepsis, respiratory and multiple organ dysfunction syndrome.

**Methods:**

We conducted a multicenter, randomized, clinical trial in patients with sepsis. Participants with ratio of partial oxygen pressure to fraction of inspired oxygen less than 200 and more than 3 SOFA points from systems other than the respiratory function were enrolled between December 2017 and September 2019. Patients were randomized to receive 1 gr of clarithromycin or placebo intravenously once daily for 4 consecutive days. The primary endpoint was 28-day all-cause mortality. Secondary outcomes were 90-day mortality; sepsis response (defined as at least 25% decrease in SOFA score by day 7); sepsis recurrence; and differences in peripheral blood cell populations and leukocyte transcriptomics.

**Results:**

Fifty-five patients were allocated to each arm. By day 28, 27 (49.1%) patients in the clarithromycin and 25 (45.5%) in the placebo group died (risk difference 3.6% [95% confidence interval (CI) − 15.7 to 22.7]; *P* = 0.703, adjusted OR 1.03 [95%CI 0.35–3.06]; *P* = 0.959). There were no statistical differences in 90-day mortality and sepsis response. Clarithromycin was associated with lower incidence of sepsis recurrence (OR 0.21 [95%CI 0.06–0.68]; *P* = 0.012); significant increase in monocyte HLA-DR expression; expansion of non-classical monocytes; and upregulation of genes involved in cholesterol homeostasis. Serious and non-serious adverse events were equally distributed.

**Conclusions:**

Clarithromycin did not reduce mortality among patients with sepsis with respiratory and multiple organ dysfunction. Clarithromycin was associated with lower sepsis recurrence, possibly through a mechanism of immune restoration.

*Clinical trial registration* clinicaltrials.gov identifier NCT03345992 registered 17 November 2017; EudraCT 2017-001056-55.

**Supplementary Information:**

The online version contains supplementary material available at 10.1186/s13054-022-04055-4.

## Introduction

Sepsis mortality remains unacceptably high, reaching 26.7% for in-hospital cases and 41.9% for patients hospitalized in the intensive care unit (ICU) [1]. It accounts for 19.7% of global deaths [2], and it is accompanied by considerable long-term morbidity [3]. Despite early recognition, timely antimicrobial administration and organ support, further adjunctive therapies are required [4]. However, the majority of potential interventions targeting the host immune response yielded conflicting results [5–11], possibly due to incomplete understanding of underlying pathophysiological mechanisms [12].

Macrolides may exert immune-modulating effects [13], as demonstrated for the exacerbation of chronic obstructive pulmonary disease [14] and for the management of community-acquired pneumonia (CAP) [15, 16]. Combination therapy with β-lactams leads to decreased mortality and is currently recommended as first-line treatment in CAP [16]. Our group investigated the immune-modulating properties of clarithromycin in severe infections caused by bacteria outside the macrolide antimicrobial spectrum, i.e., ventilator-associated pneumonia (VAP) and Gram-negative infections. Results showed that adjunctive clarithromycin, compared to placebo, improved survival among the most severely ill patients, particularly those with acute respiratory dysfunction syndrome (ARDS) [17, 18]. This benefit has been associated with improving signs of sepsis-induced immunosuppression, a hallmark of the protracted course of healthcare-associated infections [19]. This was characterized by higher production of interleukin (IL)-6 from circulating monocytes, decrease in the ratio of circulating IL-10 to TNFα (tumor necrosis factor-alpha) and increase in CD86 expression on circulating monocytes.

Based on those observations, we initiated the INtravenous CLArithromycin in Sepsis and multiple organ dysfunction Syndrome (INCLASS) study with the aim to assess the adjunctive role clarithromycin on top of standard-of-care treatment for high-risk patients with healthcare-associated infections and sepsis.

## Patients and methods

### Ethics

INCLASS was a phase 3, multi-center, randomized, placebo-controlled, double blind, clinical trial, conducted in thirteen study sites (11 multidisciplinary ICUs and 2 general Internal Medicine wards) in tertiary, teaching hospitals in Greece and Belgium (additional information in Additional files 1 and 2). The protocol and informed consent form were approved in Greece [National Organization for Medicines (51239/01-06-2017), National Ethics Committee (52086/2017)] and Belgium [Federal Agency of Medicines and Health Products (1078386/16-04-2018), Central Ethics Committee, Erasme University Hospital (P2018/376, 19-10-2018)]. Study registration was with EudraCT (2017-001056-55) and Clinicaltrials.gov (NCT03345992). Written informed consent was provided by patients or legal representatives, prior to inclusion. The complete study protocol and history of amended versions are provided in Additional files 1 and 2.

### Participants

Adults with sepsis and multiple organ dysfunction syndrome (MODS) were eligible to participate. Inclusion criteria were sepsis associated with hospital-acquired (HAP), healthcare-associated pneumonia (HCAP), VAP, primary Gram-negative bacteremia or intra-abdominal infection; PaO_2_/ FiO_2_ < 200; and total Sequential Organ Failure Assessment (SOFA) score for non-respiratory organ dysfunctions more than 3. Main exclusion criteria were pregnancy or lactation, neutropenia (< 1000 neutrophils/ mm^3^), recent high-dose corticosteroid intake, macrolide allergy and macrolide intake for the current infection. A complete list of exclusion criteria and definitions is provided in Additional file 1.

### Randomization and intervention

Patients were assigned to blind treatment with clarithromycin or placebo, following a random allocation sequence, with a 1:1 design and by block sizes of 10, stratified per study site. The allocation sequence was generated by an independent statistician prior to the study commencement and delivered to each study site within sealed individual envelopes, labeled as per study participant code. The envelope was unsealed by the study pharmacist, to prepare the study drug. All other parties involved (investigators, patients, healthcare providers, data collectors) were blinded to the study arm.

Patients were randomized to intravenous clarithromycin (1gr dissolved into 20 ml water for injection and then diluted to a final volume of 250 ml 5% dextrose in water) or placebo (equal volume of water for injection diluted to a final volume of 250 ml 5% dextrose in water), infused once daily within 1 h, for four consecutive days. The final preparations were visually similar. Other therapies were left at the discretion of the attending physicians. There was no specific time window from enrollment to study drug administration, which was required to be as soon as logistically possible.

### Procedures

Patients were followed-up daily until day 28 or hospital discharge (whichever came first). In case of earlier discharge, visit of day 28 was performed by phone call, to assess the primary outcome and safety. Another phone call was performed at day 90 with the patient or their caregiver, to assess survival. Data were captured in one Case Report Form; source data verification was performed by trained clinical research associates. Adverse events were captured daily until day 28 or discharge, and as patient or provider-reported events up to day 90. National Cancer Institute Common Terminology Criteria for Adverse Events, version 5.0 (2017), were used for classification.

Blood cell populations and monocyte human leukocyte antigen (mHLA)-DR expression were measured on days 1, 5 and 10, using flow cytometry. Total ribonucleic acid (RNA) was isolated from PAXgene blood RNA tubes (Qiagen), collected on days 1 and 5, using PaxGene Blood miRNA kits according to the manufacturer’s instructions (Qiagen). RNA-sequencing libraries were prepared using KAPA RNA Hyperprep with RiboErase (Roche) kits. Libraries were sequenced using the Illumina HiSeq4000 instrument (Illumina). All day 1 sampling was performed before study drug administration. Detailed methodology and bioinformatics are shown in Additional file 1.

### Outcomes

The primary outcome was 28-day all-cause mortality. Secondary outcomes were 90-day mortality; 28-day mortality in septic shock; early sepsis response (≥ 25% decrease in day 3 SOFA score from baseline); sepsis response (≥ 25% decrease in day 7 SOFA score from baseline); new sepsis among patients with sepsis response; the time to new sepsis episode; and differences in cell populations and gene expression.

### Statistical analysis

The sample size was calculated for the primary endpoint, anticipating a 55% 28-day mortality, among control patients with sepsis and respiratory dysfunction, and 25% reduction with clarithromycin [17, 18]. To detect this difference, with 80% power at 10% significance level, 55 patients were required in each study arm. No interim analysis was planned.

Clinical outcomes were assessed on an intention-to-treat principle, according to the pre-defined statistical analysis plan (Additional file 2). The Fisher’s two-sided exact test, confirmed by logistic regression analysis, was used to assess the primary outcome. Kaplan–Meier curves were used to assess survival by arm, and the arm effect on 28-day mortality was evaluated with Cox-regression model. Univariate and stepwise multivariable logistic regression analyses were done using pre-specified co-variables (age, sex, SOFA, Acute Pathophysiology and Chronic Health Evaluation—APACHE II, Charlson comorbidity index—CCI, and adequacy of empirical antimicrobial treatment) associated with 28-day mortality. The treatment effect on secondary outcomes was compared with the Pearson Chi-square test, or the two-sided Fisher’s exact test, if categorical, whereas quantitative variables were assessed using Student’s t test, or Mann–Whitney U test, as appropriate. Subgroup analyses were pre-planned and are shown in Additional file 1.

A number of post hoc multivariable regression analyses were performed with regard to the primary outcome, to explore whether a simplified model, adapted for the study sample size (including SOFA, CCI, appropriateness of empirical antimicrobial treatment), early versus late start of treatment and co-administered antimicrobials would impact on 28-day mortality. The impact of enrollment site was assessed by the Breslow–Day test, and heterogeneity was further evaluated by the *I*^2^ statistic. Post hoc Cox- and Poisson regression analyses were performed to assess the cumulative incidence of new sepsis and further explore study outcomes (IBM SPSS statistics, version 24.0). Any two-tailed *p* < 0.05 was considered significant. No adjustment was performed for multiple comparisons, and secondary endpoints should be interpreted as exploratory.

RNA-sequencing data analysis, as well as the *I*^2^ statistic for study site heterogeneity, was performed in R (version 3.51, R Core Team 2014). Additional information is provided in Additional file 1.

## Results

### Baseline characteristics

**Fig. 1:**
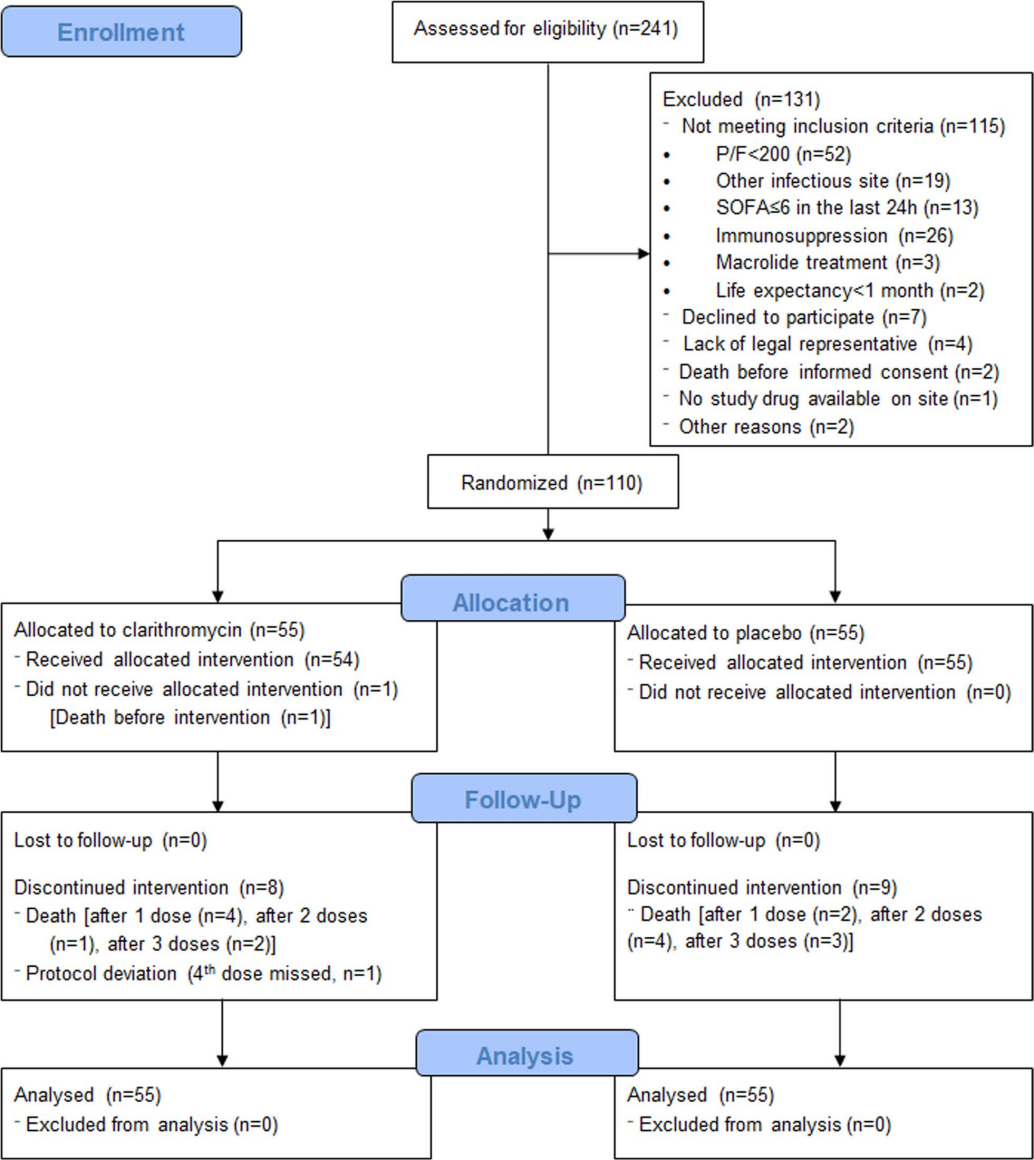
CONSORT Flow Diagram in INCLASS trial. SOFA, Sequential Organ Failure Assessment

From December 2017 to June 2019, 241 patients were assessed for eligibility and 110 were randomized to blind treatment. The first patient was enrolled on December 20, 2017, and the last visit of the last participant was on September 22, 2019. Main reasons for exclusion of patients were respiratory ratio ≥ 200, non-eligible infections and immunosuppression (Fig. 1). Fifty-five patients were included in each study arm within median 2 (1–4) days after meeting inclusion criteria and 4 (1–6) days after sepsis onset. One patient (1.8%) in the clarithromycin arm did not receive any dose of study drug due to early death, and another patient (1.8%) in the clarithromycin arm did not receive the fourth dose of the study drug due to protocol deviation judged to be a study team error. There were no losses to follow-up, nor consent withdrawals, and all 110 patients were included in the analysis of the primary endpoint and the 90-day outcomes.

Overall, baseline demographic and clinical characteristics were similar between study arms; patients were predominantly male, with high comorbidity burden and high SOFA and APACHE II scores. VAP was the most common infection (Table 1; Additional file 1: Tables S1 and S2). Enrollment by participating site is shown in Additional file 1: Table S3.

**Table 1:** Baseline characteristics of patients enrolled in the INCLASS study

	Clarithromycin (*n* = 55)	Placebo (*n* = 55)	Total (*n*= 110)	*P* value
Age (years), median (Q1–Q3)	74 (67–80)	73 (60–79)	74 (62–80)	0.354
Sex, *n* (%)				0.316
Male	39 (70.9)	33 (60.0)	72 (65.5)	
Female	16 (29.1)	22 (40.0)	38 (34.5)	
Charlson comorbidity index, mean (SD)	5.3 (2.8)	5.6 (2.8)	5.4 (2.8)	0.496
APACHE II score, mean (SD)	20.0 (6.4)	22.0 (7.1)	21.0 (6.8)	0.193
SOFA score, median (Q1-Q3)	10 (9–12)	11 (9–13)	10 (9–12)	0.364
Presence of ARDS, *n* (%)	23 (41.8)	25 (45.5)	48 (43.6)	0.848
Presence of septic shock, *n* (%)	30 (54.5)	39 (70.9)	69 (62.7)	0.114
Laboratory values, median (Q1-Q3)				
Lactate, mmol/l	1.8 (1.4–2.6)	2.1 (1.4–3.0)	2.0 (1.4–2.8)	0.459
PaO_2_/FiO_2_	142 (111–171)	149 (116–167)	144 (114–165)	0.886
White blood cell count, × 10^3^/mm^3^	15.9 (10.8–20.8)	16.7 (10.9–21.4)	16.2 (10.9–20.9)	0.832
Platelet count, × 10^3^/mm^3^	130 (196–264)	197 (138–278)	197 (137–268)	0.907
Creatinine, mg/dl	1.80 (1.05–2.41)	1.40 (0.80–2.40)	1.60 (0.87–2.4)	0.140
CRP, mg/l	169.0 (91.5–259.4)	158.0 (66.2–233.8)	162.9 (81.7–254.8)	0.543
PCT, ng/ml	1.42 (0.48–9.54)	1.36 (0.55–5.43)	1.42 (0.55–5.71)	0.900
Organ support at enrollment				
Mechanical Ventilation, n (%)	45 (81.8)	44 (80.0)	89 (80.9)	1.00
PEEP^a^, median (Q1-Q3)	8 (6–10)	8 (7–10)	8 (6–10)	0.524
Tidal Volume^a^, median (Q1-Q3)	502 (460–550)	500 (450–550)	500 (455–550)	0.638
Noradrenaline use, *n* (%)	54 (98.2)	55 (100.0)	99 (90.0)	1.00
Renal Replacement Therapy, *n* (%)	8 (14.5)	9 (16.4)	17 (15.5)	1.00
Source of sepsis, *n* (%)				
Lower respiratory tract infection	34 (61.8)	41 (74.5)	75 (68.2)	0.219
Healthcare-associated	12 (21.8)	11 (20.0)	23 (20.9)	1.00
Hospital-acquired	6 (10.9)	15 (27.3)	21 (19.1)	0.051
Ventilator-associated	16 (29.1)	15 (27.3)	31 (28.2)	1.00
Intra-abdominal infection	15 (27.3)	12 (21.8)	27 (24.5)	0.658
Primary Gram-negative bacteremia	6 (10.9)	2 (3.6)	8 (7.3)	0.271

SI conversion factors: To convert white blood cell count to 10^9^/L, multiply by 1; platelet count to 10^9^/L, multiply by 1; and (serum) creatinine to μmol/L, multiply by 88.4

APACHE, Acute Pathophysiology and Chronic Health Evaluation; ARDS, acute respiratory distress syndrome; CRP, C-reactive protein; PCT, procalcitonin; PEEP, positive end-expiratory pressure; SD, standard deviation; and SOFA, Sequential Organ Failure Assessment

^a^Refers to patients under mechanical ventilation

### Primary outcome

**Fig. 2:**
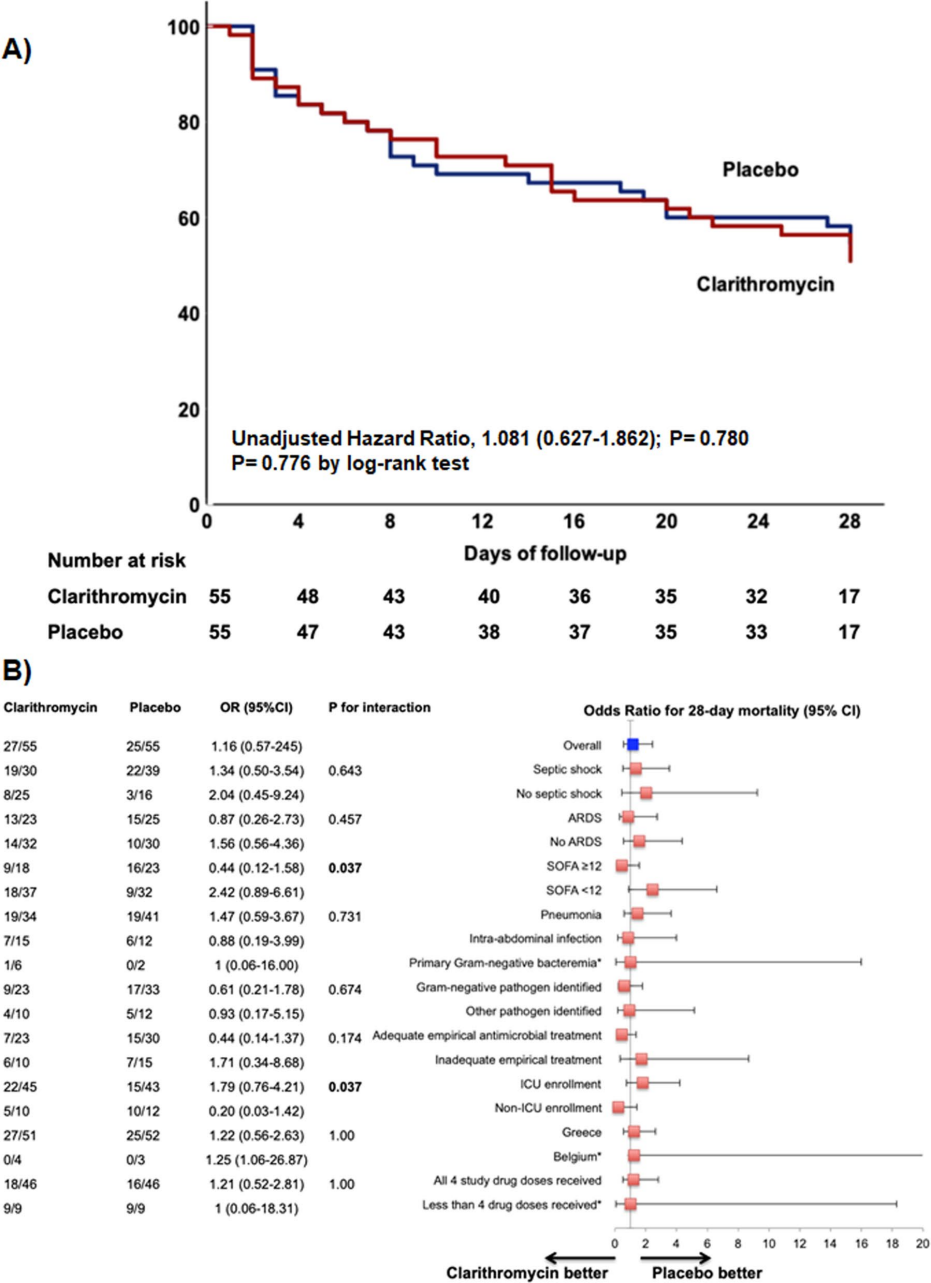
Twenty-eight-day survival among trial participants (**A**) 28-day survival analysis by Kaplan–Meier curves among patients with sepsis and multiple organ dysfunction syndrome, treated with clarithromycin or placebo. Hazard ratio is provided by Cox-regression analysis. (**B**) Risk of death within 28-days in pre-specified subgroups among patients treated with clarithromycin or placebo. *P* values for interactions between treatment arm and subgroup are provided by the Breslow–Day test. *Calculated using the Firth correction. ARDS, acute respiratory distress syndrome; CI, confidence intervals; ICU, intensive care unit; SOFA, Sequential Organ Failure Assessment

By day 28, 27 (49.1%) patients in the clarithromycin group and 25 (45.5%) in the placebo group had died, yielding an absolute difference in mortality risk of 3.6% (95%CI − 15.7 to 22.7; *P* = 0.703); the unadjusted odds ratio (OR) for clarithromycin relative to placebo was 1.16 (95% CI 0.55–2.45; *P* = 0.849) (Table 2). This was confirmed by survival analysis (Fig. 2A). After adjustment for covariates, the OR was 1.03 (95%CI 0.35–3.06; *P* = 0.604) (Additional file 1: Table S4), and the model was able to explain 30.3% of 28-day mortality variance. No heterogeneity related to study site was detected (Additional file 1: Figure S1). Study enrollment within the first 48 h from sepsis onset did not impact final outcome (Additional file 1: Table S5). In post hoc regression analysis, the study drug treatment effect remained unaltered by the type of antimicrobial treatment (Additional file 1: Tables S6 to S10).

**Table 2:** Study clinical outcomes

	Clarithromycin (n = 55)	Placebo (n = 55)	OR for clarithromycin	*P* value
Primary outcome, *n* (%, 95% CI)				
Mortality at 28 days	27 (49.1, 35.5–62.8)	25 (45.5, 32.2–59.3)	1.157 (0.547–2.448)	0.849
Secondary outcomes				
Mortality at 90 days, *n* (%, 95% CI)	41 (74.5, 60.7–84.9)	38 (69.1, 55.0–80.5)	1.310 (0.569–3.016)	0.672
Early sepsis response on day 3, *n* (%, 95% CI)	18 (32.7, 21.1–46.8)	23 (41.8, 28.9–55.9)	0.677 (0.311–1.473)	0.430
Sepsis response on day 7, *n* (%, 95% CI)	23 (41.8, 28.9–55.9)	28 (50.9, 37.2–64.5)	0.693 (0.327–1.471)	0.445
New sepsis episode until day 28^a^, *n* (%, 95% CI)	7 (30.4, 14.1–53.0)	19 (67.9, 47.6–83.4)	0.207 (0.063–0.682)	**0.012**
Days to new sepsis episode up to day 28, mean (SD)^a^ *n* (%, 95% CI)	18 (7)	15 (6)	–	0.368

CI, confidence intervals; OR, odds ratio; and SD, standard deviation

^a^Among 51 patients experiencing sepsis response, defined by SOFA decrease of ≥ 25% on day 7

No differences regarding the primary outcome were detected in any of the 8 pre-planned subgroup analyses. However, treatment impacted differently patients with extreme disease severity (within the highest quartile of SOFA) compared to those with lower SOFA scores, as well as patients enrolled in the general ward, compared to those in the ICU (Fig. 2B).

### Secondary outcomes

There were no significant differences between treatment arms in 90-day mortality (OR 1.31 [95%CI 0.57–3.05]; *P* = 0.672) (Table 2). Subgroup analysis suggested differential treatment response among patients with and without extremely severe disease, with clarithromycin favoring better outcomes among patients with SOFA score 12 or more (Additional file 1: Figure S2).

Early sepsis response by day 3 (OR 0.68 [95%CI 0.31–1.47]; *P* = 0.430) and sepsis response by day 7 did not differ (OR 0.69 [95%CI 0.33–1.47]; *P* = 0.445). Twenty-three patients in the clarithromycin group and 28 patients in the placebo group had sepsis response by day 7; in seven (30.4%) and 19 (67.9%), respectively, sepsis recurred (OR 0.21 [95%CI 0.06–0.68]; *P* = 0.012) (Table 2). In Poisson regression model, the incident rate of new septic episodes in the clarithromycin arm was lower than in the placebo arm (incident rate ratio [IRR] 0.44 [95% CI 0.19–0.99]; *P* = 0.048), and in Cox-regression analysis, survival free from new sepsis episode was significantly prolonged, following clarithromycin treatment (Additional file 1: Figure S3).

The type and pathogen of the first recurrent septic episode among patients with sepsis response by day 7 are shown in Additional file 1: Table S11 and did not differ between the two study arms. The distribution of recurrent septic sites, compared to the original infections, is presented in Additional file 1: Table S12 and mainly concerned cases of pneumonia.

### Impact of clarithromycin treatment on the host immune response

Treatment with clarithromycin was associated with an increase in non-classical monocytes on day 10 (Additional file 1: Figure S4). Counts of classical monocytes, T cells, B cells and NK cells remained unaltered. A significant increase in mHLA-DR expression was also observed among clarithromycin-treated patients on day 10, compared to placebo (Additional file 1: Figure S5).

Similarly, among those with sepsis response on day 7, patients in the clarithromycin arm presented higher levels of HLA-DR on day 10 (Additional file 1: Figure S6).

Gene set enrichment analysis identified a cholesterol homeostasis gene set upregulated in the clarithromycin-treated group, relative to placebo (Additional file 1: Results and Figures S7–9).

### Safety

At least one serious adverse event (SAE) was reported for most of the participants. SAEs were equally distributed among the groups of treatment (Table 3). Eight cases of acute kidney injury were reported as SAEs: seven in the clarithromycin arm and one in the placebo arm. Nil case was reported as associated with the study drug. With the exception of one case in each group, the other cases of acute kidney injury presented several days after the stop of the study drug. The investigators associated all eight cases either with sepsis progression or with the administration of other drugs (Additional file 1: Table S13). The two groups of treatment did not differ in the incidence of non-serious adverse events (Additional file 1: Table S14). In nil patient, the study drug was discontinued [20].

**Table 3:** Serious treatment-emergent adverse events (sTEAEs)

sTEAE, *n* (%)	Clarithromycin (*n* = 55)	Placebo (*n* = 55)	*P* value
At least one sTEAE	50 (90.9)	50 (90.9)	1.000
Infections and infestations	31 (56.4)	33 (60.0)	0.847
Acute kidney injury	7 (12.7)	1 (1.8)	0.060
Disseminated intravascular coagulation	1 (1.8)	0 (0.0)	1.00
Arterial ischemia	7 (12.7)	5 (9.1)	0.761
Cardiac disorders	7 (12.7)	5 (9.1)	0.776
Non-ST elevation myocardial infarct	2 (3.6)	1 (1.8)	0.999
Ventricular tachycardia	2 (3.6)	2 (3.6)	1.00
Pulmonary edema	3 (5.5)	2 (3.6)	1.00
Vascular disorders	8 (14.5)	6 (10.9)	1.00
Arterial ischemia	7 (12.7)	5 (9.1)	0.761
Venous thromboembolism	0 (0.0)	1 (1.8)	1.00
Hemoptysis	1 (1.8)	0 (0.0)	1.00
Hemorrhagic complications	3 (5.5)	0 (0.0)	0.243
Surgical site bleeding with hemorrhagic shock	2 (3.6)	0 (0.0)	0.495
Μetabolic and nutrition disorders	2 (3.6)	0 (0.0)	0.495
Myxedema coma	1 (1.8)	0 (0.0)	1.00
Hypoglycemia	1 (1.8)	0 (0.0)	1.00
Thoracic, pulmonary or mediastinal disorders	3 (5.5)	2 (3.6)	1.00
Airway obstruction (tracheotomy plugs)	3 (5.5)	2 (3.6)	1.00
Pneumothorax	1 (1.8)	1 (1.8)	1.00
Neurological disorders	1 (1.8)	3 (5.5)	0.618
Brain edema	0 (0.0)	1 (1.8)	1.00
Hypoxic encephalopathy	0 (0.0)	1 (1.8)	1.00
Seizures	1 (1.8)	1 (1.8)	1.00
Surgery complications	4 (7.3)	1 (1.8)	0.363
Retinal fissure detachment	0 (0.0)	1 (1.8)	1.00
Nephrostomy	1 (1.8)	0 (0.0)	1.00
Intra-abdominal retention of surgical compress	1 (1.8)	0 (0.0)	1.00
Ileal perforation	1 (1.8)	0 (0.0)	1.00
Surgical wound dehiscence	1 (1.8)	0 (0.0)	1.00
Mediastinal abscess drainage	1 (1.8)	0 (0.0)	1.00

Percentages may not add up to 100% since some patients have experienced more than one serious adverse event

## Discussion

In this randomized clinical trial, adjunctive clarithromycin treatment did not affect 28-day survival among patients with sepsis and MODS. Clarithromycin treatment was associated with fewer sepsis recurrences among patients with original sepsis response and findings compatible with modulation of the immune response toward return to homeostasis. However, due to the limited study size secondary outcomes were not adjusted for multiple comparisons and subgroup analyses showing treatment benefit need to be interpreted with caution. Overall study mortality was high. This was anticipated since the baseline SOFA score was high (median 10 in the placebo group; median 11 in the clarithromycin group).

The finding on sepsis recurrence is in agreement with the secondary benefit of clarithromycin treatment in patients with CAP. In this RCT, patients were randomized to β-lactam monotherapy or to combination of β-lactams and clarithromycin. Fewer patients receiving combination therapy were readmitted to hospital by day 30, mostly due to recurrence of pneumonia [21].

Subgroup analysis suggested 90-day survival benefit among extremely severe patients with SOFA score 12 or more. However, such a benefit was missing from patients with SOFA score less than 12 and these patients may be even harmed. However, the limited size of the study population makes this post hoc survival benefit inconclusive. The incidence of acute kidney was greater in the clarithromycin arm, although no association with the study drug was documented.

The above-described clinical benefit in the clarithromycin group may be linked with the modulation of the host immune response toward recovery from sepsis-induced immunosuppression. This is reflected by the increase in mHLA-DR expression and expansion of non-classical monocytes. Low mHLA-DR expression is a hallmark of sepsis-induced immune suppression, associated with secondary infections and mortality, while recovery is a surrogate of improved outcomes [22] and has been used to monitor immunotherapy efficacy [23, 24]. This is in line with previous observations from our group in VAP; clarithromycin treatment was associated with better ex vivo function of PBMCs, decreased IL-10/ TNFα ratio, improved antigen presentation and greater apoptosis of monocytes, consistent with immune restoration [20].

Although non-referring to the entire study population, transcriptomic data indicated that genes involved in cholesterol biosynthesis were altered in clarithromycin-treated patients, relative to placebo, at study day 5. Other data also indicate the presence of an axis between cholesterol synthesis, antigen presentation and monocyte reprogramming toward a trained immunity phenotype [25–27]. Whether this axis is modulated by clarithromycin remains to be demonstrated.

Our study had four main limitations: (a) the limited sample size to detect secondary outcomes with sufficient power and to account for multiple testing and differences among subgroups; (b) the probability for type II error. Although the anticipated 25% difference in the primary outcome used for power calculation was ambitious, the authors expected that the prognostic enrichment in MODS patients (where clarithromycin had shown maximum benefit) would translate into the same treatment effect, shown in both previous RCTs [17, 18]; (c) the high infection rate, exceeding 50%, by extremely or pan-drug-resistant isolates, particularly *Acinetobacter baumannii,* making direct comparisons with other European ICUs complicated. This may have also prevented survival benefit from being demonstrated, while lower sepsis recurrence in this high-resistance setting may be considered as a relevant clarithromycin treatment outcome; and (d) the delay in study enrollment reaching median of 4 days after sepsis onset.

## Conclusions

Among patients with sepsis and MODS, clarithromycin did not affect all-cause 28-day mortality; sepsis recurrence among patients with sepsis response by day 7 was decreased. Reprogramming of genes involved in cholesterol biosynthesis pathway in circulatory immune cells may underlie this process.

## Supplementary Information

**Additional file 1.** Contains Supplementary Methods and Results.

**Additional file 2.** Contains the complete Study Protocol versions 1 and 2, a detailed description of amendments between protocol versions and the Statistical Analysis Plan.

## Data Availability

The sequence libraries generated in this study are publicly available through the National Center for Biotechnology Information (NCBI) gene expression omnibus (GEO) under the accession number GSE196117. Requests for de-identified and protected health information (PHI)-stripped patient data can be made to the corresponding author with specific data needs, analysis and dissemination plans. Previous Institutional Review Board (IRB)/Independent Ethics Committee (IEC) approval will be required, if applicable. Dates will be time-shifted to eliminate PHI, as needed. Requests for supporting documents, such as statistical analysis plan, complete trial protocol and subsequent protocol amendments, can also be addressed to the corresponding author with specific needs, analysis and dissemination plans. All the above requests will be reviewed by the study sponsor for release, upon publication. Contact: egiamarel@med.uoa.gr.

## Abbreviations

APACHE: Acute Pathophysiology and Chronic Health Evaluation
ARDS: Acute respiratory distress syndrome
CAP: Community-acquired pneumonia
CCI: Charlson comorbidity index
HAP: Hospital-acquired pneumonia
HCAP: Healthcare-associated pneumonia
ICU: Intensive care unit
IRR: Incident rate ratio
mHLA-DR: Monocyte human leukocyte antigen-DR
MODS: Multiple organ dysfunction syndrome
OR: Odds ratio
RCT: Randomized controlled trial
RNA: Ribonucleic acid
SOFA: Sequential Organ Failure Assessment
VAP: Ventilator-associated pneumonia
