# Supplementary material for: Effect of intravenous clarithromycin in patients with sepsis, respiratory and multiple organ dysfunction syndrome: a randomized clinical trial
Source: Crit Care. 2022 Jun 18;26:183. doi: 10.1186/s13054-022-04055-4 (PMC9206755; doi:10.1186/s13054-022-04055-4)
Supplement: Supplementary file 1 — Additional file 1. Contains Supplementary Methods and Results. [file 13054_2022_4055_MOESM1_ESM.docx]

**Additional File 1**

**EFFECT OF INTRAVENOUS CLARITHROMYCIN IN PATIENTS WITH SEPSIS, RESPIRATORY AND MULTIPLE ORGAN DYSFUNCTION SYNDROME: A RANDOMIZED CLINICAL TRIAL**

**Running Title: Clarithromycin as immune modulator (INCLASS trial)**

Eleni Karakike, MD, Brendon P. Scicluna, PhD, Maria Roumpoutsou, MD^*^,

Ioannis Mitrou, MD^*^, Niki Karampela, MD, Athanasios Karageorgos, MSc,

Konstantinos Psaroulis, MD, PhD, Eleni Massa, MD, Achillefs Pitsoulis, MD,

Panagiotis Chaloulis, MD, Evanthia Pappa, MD, Irene T. Schrijver, MD, PhD,

Frantzeska Frantzeskaki, MD, PhD, Malvina Lada, MD, PhD,

Nicolas Dauby, MD, PhD, David De Bels, MD, PhD^✝^, Ioannis Floros, MD, PhD,

Souzana Anisoglou, MD, PhD, Eleni Antoniadou, MD, PhD, Maria Patrani, MD, PhD, Glykeria Vlachogianni, MD, PhD, Eleni Mouloudi, MD, PhD,

Anastasia Antoniadou, MD, PhD, David Grimaldi, MD, PhD, Thierry Roger, PhD,

W. Joost Wiersinga, MD, PhD, Iraklis Tsangaris, MD, PhD,

Evangelos J. Giamarellos-Bourboulis, MD, PhD

^*^equal contribution

^1^4^th^ Department of Internal Medicine, National and Kapodistrian University of Athens, Greece;

^2^Center for Experimental Molecular Medicine, Division of Infectious Diseases, Amsterdam University Medical Centers, Academic Medical Center, University of Amsterdam, Amsterdam, the Netherlands;

^3^Department of Applied Biomedical Science, Faculty of Health Sciences, Mater Dei hospital, University of Malta, Msida, Malta;

^4^Centre for Molecular Medicine and Biobanking, University of Malta, Msida, Malta.

^5^Intensive Care Unit, Korgialeneio Benakeio General Hospital, Athens, Greece;

^6^Intensive Care Unit, Aghios Dimitrios General Hospital, Thessaloniki, Greece;

^7^Intensive Care Unit, Hippokration General Hospital, Thessaloniki Greece;

^8^Intensive Care Unit, G. Gennimatas General Hospital, Thessaloniki, Greece;

^9^Intensive Care Unit, Theageneion General Hospital, Thessaloniki, Greece;

^10^Intensive Care Unit, Laiko General Hospital, Athens, Greece;

^11^ Infectious Diseases Service, Department of Medicine, Lausanne University Hospital and University of Lausanne, Lausanne, Switzerland;

^12^2^nd^ Department of Critical Care Medicine, National and Kapodistrian University of Athens, Greece;

^13^2^nd^ Department of Internal Medicine, Sismanogleion General Hospital, Athens, Greece;

^14^Department of Infectious Diseases, Centre Hospitalier Universitaire Saint-Pierre, Université Libre de Bruxelles (ULB), Belgium;

^15^Institute for Medical Immunology, Université Libre de Bruxelles (ULB)

^16^Department of Intensive Care, Centre Hospitalier Universitaire Brugmann, Brussels, Belgium;

^17^Department of Intensive Care, CUB-Erasme, Université Libre de Bruxelles (ULB), Brussels, Belgium

**Corresponding Author**

Evangelos J. Giamarellos-Bourboulis, MD, PhD

4^th^ Department of Internal Medicine, ATTIKON University General Hospital

1 Rimini Street 124 62 Athens, Greece

Tel: +30 210 58 31 994/ Fax: +30 210 53 26 446

E-mail: [egiamarel@med.uoa.gr](mailto:egiamarel@med.uoa.gr)

**SUPPLEMENTARY METHODS**

**Participating sites**

All sites complied with Surviving Sepsis Campaign guidelines^1^ and with local antimicrobial recommendations,^2-4^ as assessed during feasibility visits.

**Detailed Exclusion Criteria**

Patients meeting ANY of the criteria below could not be enrolled in the study:

- Denial for informed consent
- Age inferior to 18 years
- Pregnancy (confirmed by blood or urinary pregnancy test) or lactation for female patients of reproductive age.
- Unwillingness to receive contraception during and seven days after the administration of the study drug.
- HIV infection (with known CD4 cell count ≤ 200/mm^3^)
- Solid organ, or bone marrow transplantation
- Corticosteroid oral or intravenous intake greater than 0.4 mg/kg of equivalent prednisone daily over the last 15 days; anti-cytokine biological agents (e.g. anti-TNFα), anti-lymphocyte immunoglobulins, Mycofenolate Mofetil, Tacrolimus (FK506) and m-TOR inhibitors (any dose of the above within the past 3 months); Chemotherapy within the past 3 months, Leflunomide intake within the past 2 year, Rituximab within the last year; Methotrexate, Azathioprine, Cyclosporine, Cyclophosphamide (any dose of the above within the last 3 months). Splenectomy, known primary immunodeficiencies. Hydroxyurea, anti-Vascular Endothelial Growth Factor (VEGF), anti-Epithelial Growth Factor Receptor (EGFR), anti-Growth Factor Her-2 and Interferon a, b and γ intake are not considered as exclusion criteria.
- Known active neoplasms or other medical conditions unrelated to sepsis (any of the two), that are compromising short-term survival (1 month)
- Neutropenia <1000/mm^3^
- Known allergy to macrolides
- Previous participation in the study
- Administration of a macrolide for the current infectious episode

**Precisions and rationale on inclusion and exclusion criteria**

Inclusion infections were selected to meet previous evidence of clarithromycin benefit in sepsis (Ventilator-associated pneumonia, Gram-negative infections).^5,6^ Healthcare-associated infections, likely to be associated with Gram-negative pathogens, were preferred over Community-acquired pneumonia, to avoid any potential antimicrobial role of clarithromycin and to prioritize its immune-modulatory effects. The respiratory dysfunction criterion (PaO_2_/FiO_2_<200) was compatible with previously reported benefit in Acute Respiratory Dysfunction Syndrome (ARDS), while non-respiratory sequential organ failure assessment (SOFA) score >3 was selected to reflect multiple organ dysfunction syndrome (MODS) and to account for the subgroup of patients that showed survival benefit in previous sepsis trials.^5,6^

Regarding contraception, it was recommended during the consent process to either opt for abstinence from intercourse, or chemical or mechanical contraception during the 7 days following drug administration.

**Definitions**

Sepsis and Septic shock were defined per Sepsis-3 criteria.^7^ Pre-existing (to sepsis onset) organ failures were assessed during screening. More precisely, eligible patients with renal failure, requiring renal replacement therapy (CRRT) at screening, were attributed a renal SOFA score of 3 or 4, if diuresis was not conserved (<500 ml and <200 ml/24h respectively) or a score according to the worst known creatinine level.

Definitions for the main infections were as following:^8,9^

Hospital-acquired pneumonia (HAP) was defined by the presence of a new or progressive radiographic lung infiltrate in a non-intubated patient hospitalized for more than 48 hours who presents with at least two of the following clinical features:

- Core temperature equal or greater than 38°C
- Total white blood cell count more than 12,000/mm^3^
- Rales or bronchial breath sounds on physical examination
- Purulent sputum
- More than 20 breaths/minute
- Serum procalcitonin more than 0.25 ng/ml
- Gram stain of tracheobronchial secretions or bronchoalveolar lavage fluid indicating the predominance of Gram-negative bacilli

Health-care associated pneumonia (HCAP) was defined by the presence of a new or progressive radiographic lung infiltrate in a non-intubated patient who has at least one of the following risk factors for HCAP:

- Hospitalization the last 90 days
- Residency in a long-term care facility
- Under regular hemodialysis

AND who presents with at least two of the following clinical features:

- Core temperature equal or greater than 38°C
- Total white blood cell count more than 12,000/mm^3^
- Rales or bronchial breath sounds on physical examination
- Purulent sputum
- More than 20 breaths/minute
- Serum procalcitonin more than 0.25 ng/ml
- Gram stain of tracheobronchial secretions or bronchoalveolar lavage fluid indicating the predominance of Gram-negative bacilli

Ventilator-associated pneumonia (VAP) was defined by the presence of a new or progressive radiographic lung infiltrate in a patient who is under mechanical ventilation for at least 48 hours AND who presents with at least two of the following clinical features:

- Core temperature equal or greater than 38°C
- Total white blood cell count more than 12,000/mm^3^
- Purulent tracheobronchial secretions
- Serum procalcitonin more than 0.25 ng/ml
- Gram stain of tracheobronchial secretions or bronchoalveolar lavage fluid indicating the predominance of Gram-negative bacilli

Primary Gram-negative bacteremia (bloodstream infection-BSI) was defined as the isolation of at least one Gram-negative microorganism from a blood culture of a peripheral vein of a patient that is not related to infection of a central line and who presents with ALL the following features:

- Core temperature equal or greater than 38°C or total white blood cell count more than 12,000/mm^3^
- Thorough clinical and radiological investigation has failed to identify the primary infection site

Intra-abdominal infection (IAI) was defined as the presence of ALL the following features^9^:

- Core temperature equal or greater than 38°C or total white blood cell count more than 12,000/mm^3^
- Radiological findings from abdominal ultrasound or abdominal computed tomography or magnetic resonance imaging consistent with one IAI or peri-operative confirmation of an IAI.

Other infections identified during follow-up (secondary infections) were defined by conventional criteria.^9, 10^ Only clinically relevant, systematic infections (HAP, VAP, IAI, primary bacteremia, Catheter-related bloodstream infection-CRBSI, acute bacterial skin and soft structures infection-ABSSSI, acute pyelonephritis, catheter-associated urinary tract infection-CA-UTI with systemic symptoms, invasive mycosis), were considered as secondary infections, while localized infections (ventilator-associated tracheo-bronchitis, upper respiratory tract infections, cystitis, oral thrush, herpetic stomatitis) where not. Secondary infections were considered as (a) new if a new infectious site was identified, unrelated to the initial infection that led to enrollment, (b) recurrent, if occurring at the same site as the initial infection that led to enrollment AND if the initial infection was previously considered resolved by the attending physician. Additional pathogens identified during follow-up that were related to a non-resolved underlying (initial) infection were not considered as new infections.

Acute Respiratory Distress Syndrome (ARDS) was defined by the Berlin criteria.^11^

Empirical antimicrobial treatment, defined as the treatment administered during the first 48 hours from infection onset was considered as adequate when the pathogen(s) was subsequently identified and susceptible to at least one of the administered antimicrobial agents (by documented antimicrobial susceptibility testing or by reasonably expected susceptibility-e.g. metronidazole for clostridioides difficile), and when frequency and dosing regimen were optimal for the site of infection identified, according to local antimicrobial guidelines.^3,4^ Adequacy of antimicrobial regimen was assessed by investigators and verified by a medical monitor with training in infectious diseases, blinded to the study arm.

Sepsis response, as secondary outcome, is defined as following:

- Early sepsis response is defined by an at least 25% decrease of day 1 SOFA score on day 3 [Δ_SOFAday3🡪1_ ≥25%, where Δ_SOFAday3🡪1_ = (SOFA_day3_-SOFA_day1_)*100/SOFA_day1_]
- Sepsis response is defined by an at least 25% decrease of day 1 SOFA score on day 7 [Δ_SOFAday7🡪1_ ≥25%, where Δ_SOFAday7🡪1_ = (SOFA_day7_-SOFA_day1_)*100/SOFA_day1_].

This 25% decrease of the SOFA score from baseline has been suggested elsewhere.^12^

**Amendment History**

During the study, a substantial amendment of the study was submitted both in Greece and Belgium regulatory authorities, in order to inform on the change of one Principal Investigator (Dr M. Patrani replacing Dr K. Mandragos at Korgialeneio- Benakeio Hospital, Athens, Greece), to comply with the newest General Data Protection Regulation (GDPR), but also to include (as minor change) the assessment of Quality of Life at the end of the 90-day follow-up, as part of a cost-utility analysis. The current amended Study Protocol Version 2 (05/11/2018) and ICF Version 2.1 (GR, 05/02/2019), were approved by the National Organization for Medicines of Greece on the 26/06/2019 (notification) and the National Ethics Committee of Greece on the 21/06/2019 (Ref Number 62985/2019), as well as the Federal Agency Medicines and Health Products of Belgium on the 20/05/2019 (notification) and the Central Ethics Committee of Erasme Hospital (Brussels, Belgium), on 11/06/2019 (Ref Number A2019/087). Study registration was with EudraCT (2017-001056-55) and Clinicaltrials.gov (NCT03345992)

**Data Measurements and Procedures**

Patients were followed-up daily until day 28 or hospital discharge (whichever came first). Clinical data were captured in a Case Report Form (CRF); source data verification was performed by trained clinical research associates. Day 1 was considered as the day of drug administration. Day 1 data were collected from 8h00 the previous day until the minute of study drug infusion start. Day 2 started from the infusion of the study drug on day 1 until 7:59 of day 3. All the other days were calculated as a period of 23 h 59 min starting at 8:00 am local time and ending at 07:59 on the next day.

The worst laboratory and vital parameters of the day were recorded in the CRF. Noradrenaline daily dose was calculated based the maximum daily rate administered (provided that it was administered for at least 2 hours). For patients requiring CRRT, a renal SOFA score of 3 or 4 was attributed during CRRT, if diuresis was not conserved (<500 ml and <200 ml/24h respectively) and/or a score according to the worst known creatinine level off CRRT, otherwise. In case of missing parameters required to calculate daily SOFA score, the last laboratory observation was carried forward solely for the purposes of SOFA calculation. If non-evaluable due to sedation, neurological SOFA score was calculated by the estimated Glasgow Coma Scale (GSC), based on the prior known GCS before sedation. For respiratory SOFA, if blood gas analysis was not performed- not considered useful by the attending physicians, paO2 was extracted by the SaO2, as previously described.^13^ SOFA score was considered as 24 on the day of death and thereafter, while SOFA score in case of discharge remained the same as the last day of hospital stay.

Missing values in all other cases were treated as such, with the number and percentage of patients provided.

**Cell population analysis** **by flow cytometry**

Blood cell populations and monocyte human leukocyte antigen (mHLA)-DR expression on days 1 (before), 5 and 10 (after randomization) were obtained by flow cytometry analyses. One hundred microliters of EDTA-anticoagulated whole blood were added to custom-made and IM phenotyping basic**^®^** DURAClone tubes (Beckman Coulter, Indianapolis, IN USA), containing antibodies (clone/fluorochrome) directed against human CD11b (Bear1/PE-Cy7), CD3 (UCHT1/APC-AF700), CD14 (RMO52/APC-AF750), CD15 (80H5/Pacific Blue), CD16 (3G8/ECD), CD19 (J3-119/APC-AF700), CD33 (D3HL60.251/APC), CD45 (J33/Krome Orange), CD56 (NKH-1/APC-AF700), CD124 (G077F6/PE) and HLA-DR (Immu-357/FITC). After 20 minutes, 900 μL of 1 x BD FACS™ lysing solution (BD Biosciences, San Jose, CA) were added. Tubes were vortexed and frozen at -80°C. Samples were thawed, washed with PBS containing 0.5% BSA (A7906, Sigma-Aldrich, Saint-Louis, MI) and 0.02% sodium azide (71289, Sigma-Aldrich) and acquired in a single day using an Attune NxT Flow Cytometer (Thermo Fisher Scientific, Waltham, MA, USA). Debris, doublets and CD45 negative cells were excluded from analysis by manual gating using FlowJo™ (v10.6.2, Ashland, OR). Unsupervised clustering was performed using FlowSOM applied to biexponential transformed and normalized expression levels of CD11b, CD14, CD15, CD16, CD33, CD45, CD124, HLA-DR and the lineage markers (CD3, CD19, CD56). The populations were analyzed as absolute counts and as percentage of total leukocytes. HLA-DR molecules on CD14^+^CD45^+^ cells were expressed as mean fluorescent intensity (MFI).

**Transcriptomic analysis**

Sampling was performed on days 1 (before) and 5 (after randomization), for all consenting patients, using PAXgene blood RNA tubes (BD Biosciences). A separate optional informed consent form was required for Belgium. At the time of the study Statistical Analysis Plan elaboration (e-Appendix 2), only a subset of sample sequencing could be covered by the study funding. Thus, only patients with extreme phenotypes (i.e. the upper quartile of SOFA score distribution) were included in the current analysis. Samples from 7 age-, sex-, and Charlson comorbidity index-matched control subjects without sepsis, enrolled within the prospective observational registry of the Hellenic Sepsis Study Group (55 study sites across Greece under the respective Ethics Committee approval) were used to assess differential gene expression with INCLASS patients.

**RNA isolation**

Total RNA was isolated from PAXgene blood tubes using PaxGene Blood miRNA kits, according to the manufacturer’s instructions (Qiagen). RNA quality was assessed by bioanalysis (Agilent), with all samples having RNA integrity numbers > 6.0. Total RNA concentrations were determined by Qubit® 2.0 Fluorometer (Life Technologies, Carlsbad, CA, USA).

**RNA sequencing and bioinformatics**

RNA-sequencing libraries were prepared from 200ng total RNA using KAPA RNA Hyperprep with RiboErase (Roche) library kits. Libraries were sequenced using the Illumina HiSeq4000 instrument (Illumina), to generate single reads (50bp). The sequencing depth was approximately 40 million reads per sample. Sequence read quality was assessed by means of the FastQC method (v0.11.5; <http://www.bioinformatics.babraham.ac.uk/projects/fastqc/>). Trimmomatic version 0.36^14^ was used to trim Illumina adapters and poor-quality bases (trimmomatic parameters: leading=3, trailing=3, sliding window=4:15, minimum length=40). The remaining high-quality reads were used to align against the Genome Reference Consortium human genome build 38 (GRCh38).^15^ Mapping was performed by HISAT2 version 2.1.0^16^ with parameters as default. Count data were generated by means of the HTSeq method.^17^ After filtering transcripts with <10 counts, the remaining transcripts were further analyzed using the DESeq2 method.^18^ Statistically significant differences were defined by Benjamini & Hochberg adjusted probabilities < 0.05 and absolute fold expression ≤−1.5 or ≥1.5). Biological pathway analysis was performed by Gene Set Enrichment analysis (GSEA), specifying the C1 Hallmarks database.^19^ An adjusted p < 0.05 was used to control false-discovery-rates and demarcated significantly enriched gene sets. Sequence libraries are publicly available through the National Center for Biotechnology Information (NCBI) gene expression omnibus (GEO) under the accession number GSE196117.

**Additional Statistical analyses**

Primary and secondary clinical outcomes were assessed on an intention-to-treat basis, according to the predefined statistical analysis plan (e-Appendix 2). Categorical variables were expressed as counts and percentages. Continuous variables with normal distribution, as assessed by the Kolmogorov-Smirnov test, were presented with their mean and standard deviation (SD); those with skewed distribution were presented with their median and quartiles (Q1-Q3).

The following subgroup analyses were planned with regards to the primary outcome and 90-day mortality: per country; per ward of enrollment (ICU or general ward); per protocol (according to whether the study intervention was complete -all 4 doses received); per presence of septic shock (already present in the protocol); per highest SOFA score (defined as higher or lower than the upper SOFA quartile of the population); per presence of ARDS; per initial infectious site; per adequacy of empirical antimicrobial treatment; and per Gram-negative vs non-Gram negative pathogen, among infections with identified pathogens. Interactions between subgroups and arm of treatment were assessed with Breslow-Day test and confirmed with logistic regression for the primary outcome and 90-day mortality.

Missing data were assessed by the Little’s test of Missing Completely at Random (MCAR) and were not imputed.

**SUPPLEMENTARY RESULTS**

**RESULTS OF TRANSCRIPTOMIC ANALYSIS**

Twenty patients with extremely severe disease (i.e. the upper quartile of SOFA score distribution, Methods, Additional file 1) were included in the transcriptomic analysis. After final quality assessment, 18 patients from day 1 (10 patients in the clarithromycin and 8 patients in the placebo arm) and 15 patients from day 5 (8 patients in the clarithromycin and 7 patients in the placebo arm) were analyzed; seven healthy subjects matched for gender, age and Charlson comorbidity index (CCI) were used as control subjects. Principal component analysis of the 31,710 expressed transcripts revealed clear partitioning between patients of the INCLASS study and control subjects (Figure S7, Additional file 1). Differential expression analysis of INCLASS patient samples compared to control subjects, including age and sex as covariates, identified 6,951 and 4,960 significant transcripts (adjusted p<0.01, fold expression <-1.2 or >1.2), respectively on days 1 and 5 (Figures S8A and 8B, Additional file 1). Differential expression analysis of day 1 and 5 samples in clarithromycin relative to placebo-treated patients revealed upregulation of *ALOX15* (a protein-coding gene involved in lipid metabolism) observed in the clarithromycin group on day 1, and downregulation of Aldehyde Dehydrogenase 1 Family Member L2 (*ALDH1L2)* and ChaC Glutathione Specific Gamma-Glutamylcyclotransferase 1 (*CHAC1*, both protein-coding genes involved in folate metabolism and cell maturation/ differentiation/ oxidative balance respectively) in the clarithromycin group on day 5 (Figures S8C and S8D). Gene set enrichment analysis of day 5 samples identified a cholesterol homeostasis gene set upregulated in the clarithromycin-treated group, relative to placebo (Figure S9A). In targeted gene analysis, genes that include Mevalonate Kinase (*MVK*), Squalene Epoxidase (*SQLE*), Sterol-C5-Desaturase (*SC5D*), Mevalonate Diphosphate Decarboxylase (*MVD*) and StAR Related Lipid Transfer Domain Containing 4 (*STARD4*) were significantly altered in clarithromycin-, relative to placebo-treated patients at study day 5 (Figure S9B).

**Table S1. Additional patient characteristics at baseline**

|  | **Clarithromycin (n=55)** | **Placebo (n=55)** | **Total (n=110)** |
| --- | --- | --- | --- |
| Comorbidities, n (%) |  |  |  |
| Type 2 diabetes mellitus | 14 (25.5) | 21 (38.2) | 35 (31.8) |
| Chronic heart failure | 12 (21.8) | 17 (30.9) | 29 (26.4) |
| Chronic renal disease | 12 (21.8) | 6 (10.9) | 18 (16.4) |
| Coronary heart disease | 11 (20.0) | 11 (20.0) | 22 (20.0) |
| Cerebrovascular disease | 12 (21.8) | 11 (20.0) | 23 (20.9) |
| Chronic pulmonary disease | 14 (25.5) | 26 (47.3) | 40 (36.4) |
| Dementia | 11 (20.0) | 7 (12.7) | 18 (16.4) |
| Malignancy | 9 (16.4) | 8 (14.5) | 17 (15.5) |
| Obesity (BMI ≥30 kg/m^2^) | 16 (29.1) | 21 (38.2) | 37 (33.6) |
| Residency in LTCF | 6 (10.9) | 6 (10.9) | 12 (10.9) |
| Admission diagnosis, n (%) |  |  |  |
| Infection/ sepsis | 29 (52.7) | 28 (50.9) | 57 (51.8) |
| Medical, non-infectious | 10 (18.2) | 13 (23.6) | 23 (20.9) |
| Surgery | 10 (18.2) | 9 (16.3) | 19 (17.3) |
| Trauma | 6 (10.9) | 5 (9.1) | 11 (10.0) |
| Time to enrollment (days) |  |  |  |
| From sepsis onset | 4 (1-6) | 4 (1-6) | 4 (1-6) |
| Enrollment within 48h, n (%) | 22 (40.0) | 20 (36.4) | 42 (38.2) |
| Enrollment >48h later, n (%) | 33 (60.0) | 35 (63.6) | 68 (61.8) |
| From meeting inclusion criteria | 2 (1-4) | 2 (1-4) | 2 (1-4) |
| From informed consent | 0 (0-1) | 0 (0-1) | 0 (0-1) |
| From hospital admission | 9 (4-18) | 9 (4-17) | 9 (4-17) |
| From ICU admission^a^ | 4 (2-12) | 5 (2-9) | 4 (2-9) |
| Vital signs, median (Q1-Q3) |  |  |  |
| Glasgow Coma Scale | 13 (10-15) | 12 (8-14) | 13 (9-15) |
| Heart rate (bpm) | 100 (77-110) | 103 (80-120) | 101 (80-114) |
| Mean Arterial Pressure (mmHg) | 68.3 (59.7-82.0) | 66.3 (55.3-76.7) | 67 (60-78) |
| Respiratory rate (per min) | 22 (18-28) | 20 (18-25) | 21 (18-26) |
| Temperature (°C) | 37.9 (36.4-38.5) | 38.0 (37.4-38.8) | 38.0 (37.0-38.6) |
| Urine output (ml/ 24h) | 2460 (1605-3500) | 2100 (1300-3430) | 2300 (1350-3480) |
| Laboratory parameters, median (Q1-Q3) |  |  |  |
| Hemoglobin, (g/dl) | 9.4 (8.4-11.3) | 9.9 (8.7-11.0) | 9.7 (8.5-11.0) |
| AST, (IU/l) | 42 (26-75) | 42 (22-91) | 42 (24-79) |
| ALT, (IU/l) | 29 (16-52) | 29 (13-104) | 29 (15-66) |
| Bilirubin, (mg/dl) | 0.7 (0.4-1.3) | 0.8 (0.5-1.9) | 0.7 (0.5-1.5) |
| PT, (sec) | 14.1 (12.3-16.0) | 14.3 (12.9-16.2) | 14.2 (12.7-16.1) |
| aPTT, (sec) | 35.4 (31.5-41.5) | 38.1 (31.6-48.3) | 36.3 (31.6-44.0) |
| Fibrinogen, (mg/dl) | 433 (362-628) | 564 (354-732) | 460 (359-702) |
| Treatment on day 1, n (%) |  |  |  |
| Antimicrobials |  |  |  |
| Carbapenems | 25 (45.5) | 20 (36.4) | 45 (40.9) |
| Novel β-lactamase inihibitors | 12 (21.9) | 14 (15.4) | 26 (13.2) |
| Piperacilllin/tazobactam | 14 (25.5) | 17 (30.9) | 31 (28.2) |
| Colisitn | 22 (40.0) | 29 (52.7) | 51 (46.4) |
| Aminoglycosides | 6 (10.9) | 6 (10.9) | 12 (10.9) |
| Cefepime | 2 (3.6) | 4 (7.3) | 6 (5.5) |
| Glycopeptides | 13 (23.6) | 12 (21.8) | 25 (22.7) |
| Linezolid | 15 (27.3) | 10 (18.2) | 25 (22.7) |
| Daptomycin | 2 (3.6) | 3 (5.5) | 5 (4.5) |
| Antifungals | 22 (40.0) | 21 (38.2) | 43 (39.1) |
| Combinations of antimicrobials |  |  |  |
| Colistin combinations^b^ |  |  |  |
| Colistin and carbapenems | 11 (20.0) | 11 (20.0) | 22 (20.0) |
| Colistin and novel β-lactamase inihibitors | 6 (10.9) | 5 (9.1) | 11 (10.0) |
| Colistin and/or other^c^ | 5 (9.1) | 13 (23.6) | 18 (16.4) |
| Number of antimicrobials (excluding antifungals) |  |  |  |
| Up to two | 35 (63.6) | 32 (58.2) | 67 (60.9) |
| More than two | 20 (36.4) | 23 (41.8) | 43 (39.1) |
| Number of antimicrobials (including antifungals) |  |  |  |
| Up to two | 26 (47.3) | 22 (40.0) | 48 (43.6) |
| More than two | 29 (52.7) | 33 (60.0) | 62 (56.4) |
| Gram-positive activity |  |  |  |
| No | 27 (49.1) | 29 (52.7) | 56 (50.9) |
| Yes | 28 (50.9) | 26 (47.3) | 54 (49.1) |
| Dobutamine | 2 (3.6) | 3 (5.5) | 5 (4.5) |
| Vasopressin | 4 (7.3) | 4 (7.3) | 8 (7.3) |
| Epinephrine | 1 (1.8) | 1 (1.8) | 2 (1.8) |
| Corticosteroids (hydrocortisone) | 19 (34.5) | 29 (52.7) | 48 (43.6) |
| Blood product transfusion | 6 (10.9) | 8 (14.5) | 14 (12.7) |
| Crystalloid Fluids (ml) | 1200 (500-2200) | 1000 (0-1920) | 1059 (386-2000) |
| Crystalloid Fluids (ml/kg) | 14 (8.3-32.8) | 11.9 (0.0-19.2) | 12.5 (3.5-24.3) |

^a^ Among 88 patients (45 in the clarithromycin and 43 in the placebo group) enrolled in the ICU

^b^ Among 51 patients treated with colistin

^c^ Other combinations for colistin referred to tigecycline, β-lactamase inihibitors, aminoglycosides, 3^rd^ generation cephalosporin or combinations of those agents

SI conversion factors: To convert hemoglobin to g/l, multiply by 10; AST and ALT to μkat/L, multiply by 0.0167; bilirubin to μmol/L, multiply by 17.104; fibrinogen to g/l, multiply by 0.01.

Abbreviations: ALT: alanine aminotransferase; aPTT: activated partial thromboplastin time; AST: aspartate aminotransferase; BMI: body mass index; ICU: intensive care unit; LTCF: Long-term care facility; PT: prothrombin time

**Table S2. Characteristics of baseline infections**

|  | **Clarithromycin (n=55)** | **Placebo (n=55)** | **Total (n=110)** |
| --- | --- | --- | --- |
| Associated bacteremia, n (%) | 19 (34.5) | 23 (41.8) | 42 (38.2) |
| Pathogen identified, n (%) |  |  |  |
| At least 1 pathogen | 33 (60.0) | 45 (81.8) | 78 (70.9) |
| 2 pathogens | 15 (27.3) | 17 (30.9) | 32 (29.1) |
| ≥3 pathogens | 6 (10.9) | 8 (14.5) | 14 (12.7) |
| Resistance pattern^a^ |  |  |  |
| None | 7 (21.2) | 9 (20.0) | 16 (20.5) |
| Multi Drug-Resistant | 10 (30.3) | 9 (20.0) | 19 (24.4) |
| Extremely Drug-Resistant | 15 (45.5) | 22 (48.9) | 37 (47.4) |
| Pan Drug-Resistant | 1 (3.0) | 5 (11.1) | 6 (7.7) |
| Pathogen type^a^, n (%) |  |  |  |
| *Acinetobacter baumannii* | 11 (33.3) | 22 (48.9) | 33 (42.3) |
| *Klebsiella pneumoniae* | 7 (21.2) | 13 (28.9) | 20 (25.6) |
| *Pseudomonas aeruginosa* | 5 (15.2) | 7 (15.6) | 12 (15.4) |
| *Escherichia coli* | 7 (21.2) | 3 (6.7) | 10 (12.8) |
| *Candida spp* | 4 (12.1) | 3 (6.7) | 7 (9.0) |
| *Enterobacter spp* | 4 (12.1) | 2 (4.4) | 6 (7.7) |
| *Enterococcus spp* | 7 (21.2) | 5 (11.1) | 12 (15.4) |
| *Staphylococcus aureus* | 3 (9.1) | 4 (8.9) | 7 (9.0) |
| Other | 6 (18.2) | 6 (13.3) | 12 (15.4) |
| Adequate empirical antimicrobial treatment)^a^, n (%) | 23 (69.7) | 30 (66.7) | 53 (67.9) |
| Source control^b^, n (%) | 11 (21.8) | 9 (16.3) | 20 (18.2) |

^a^ Assessed among 78 infections with identified pathogen (resistance pattern was defined by the most resistant pathogen of the initial infection)

^b^ Source control was required for 22 infections and performed in 20 of them (2 patients were considered inoperable)

**Table S3. Patient enrollment by study site**

|  | Principal Investigator | Address | N enrolled |
| --- | --- | --- | --- |
| 2^nd^ Department of Critical Care Medicine, Attikon University Hospital | Prof A. Armaganidis | 1, Rimini str 12462, Athens, Greece | 31 |
| 4^th^ Department of Internal Medicine, Attikon University Hospital | Prof A. Antoniadou | 1, Rimini str 12462, Athens, Greece | 20 |
| Intensive Care Unit, Agios Dimitrios General Hospital | Dr. G. Vlachogianni | 2 Elenis Zografou street, 54634, Thessaloniki, Greece | 10 |
| Intensive Care Unit, Ippokratio General Hospital | Dr E. Mouloudi | 49 Konstantinoupoleos street, 54642, Thessaloniki, Greece | 10 |
| Intensive Care Unit, Korgialenio-Benakio General Hospital | Dr M. Patrani | 1 Athanasaki street, 11526, Athens, Greece | 9 |
| Intensive Care Unit, G. Gennimatas General hospital | Dr E. Antoniadou | 41 Ethnikis Aminas street, 54635, Thessaloniki, Greece | 8 |
| Intensive Care Unit, Theagenio Cancer Hospital | Dr. S. Anisoglou | 2 Simeonidi street, 54639 Thessaloniki, Greece | 7 |
| Intensive Care Unit, Laiko General Hospital | Dr. I. Floros | 1 Agiou Thoma street, 11527, Athens, Greece | 6 |
| Department of Critical Care CUB Hopital Erasme | Prof D. Grimaldi | 808, route de Lennik, 1070, Brussels, Belgium | 5 |
| Intensive Care Unit, Centre Hospitalier Universitaire, Brugmann- Site Horta | Prof D. De Bels | 4, Place A. Van Gehuchten, 1020, Brussels, Belgium | 1 |
| Intensive Care Unit, Centre Hospitalier Universitaire, Brugmann- Site Brien | Prof D. De Bels | 36, Avenue du Foyer Schaerbeekois, 1030, Brussels, Belgium | 0 |
| Intensive Care Unit, Centre Hospitalier Universitaire, Saint-Pierre | Prof S. De Wit | 322, rue Haute, 1000, Brussels, Belgium | 1 |

**Table S4. Post-hoc regression analysis for variables associated with 28-day mortality**

|  | **Survivors (n=58)** | **Non-survivors (n=52)** | **Unadjusted OR (95%CI)** | **P-value** | **Adjusted OR (95%CI)** | **P-value** |
| --- | --- | --- | --- | --- | --- | --- |
| Clarithromycin treatment | 28 (48.3) | 27 (51.9) | 1.18 (0.55-2.45) | 0.849 | 1.03 (0.35-3.06) | 0.959 |
| Charlson Comorbidity Index | 4 (2-6) | 7 (5-8) | 1.34 (1.14-1.58) | **3.36x10^-4^** | 1.52 (1.23-1.88) | **1.12x10^-4^** |
| SOFA score on day 1 | 10 (9-12) | 11 (9-13) | 1.12 (0.95-1.32) | 0.161 | 1.16 (0.94-1.43) | 0.166 |
| Adequacy of empirical antimicrobial treatment | 31 (53.4) | 22 (42.3) | 0.66 (0.25-1.70) | 0.386 | 0.43 (0.14-1.36) | 0.108 |

Abbreviations SOFA: sequential organ failure assessment

**Table S5. Post-hoc regression analysis for variables associated with 28-day mortality, accounting for time delay from sepsis onset to enrollment.**

|  | **Survivors (n=58)** | **Non-survivors (n=52)** | **Unadjusted OR (95%CI)** | **P-value** | **Adjusted OR (95%CI)** | **P-value** |
| --- | --- | --- | --- | --- | --- | --- |
| Clarithromycin | 28 (48.3) | 27 (51.9) | 1.18 (0.58-2.45) | 0.849 | 0.94 (0.31-2.89) | 0.920 |
| Charlson Comorbidity Index | 4 (2-6) | 7 (5-8) | 1.34 (1.14-1.58) | **3.36x10^-4^** | 1.52 (1.23-1.88) | **1.12x10^-4^** |
| SOFA score on day 1 | 10 (9-12) | 11 (9-13) | 1.12 (0.95-1.32) | 0.161 | 1.17 (0.94-1.46) | 0.153 |
| Appropriate empirical antimicrobial treatment based on guidelines | 31 (53.4) | 22 (42.3) | 0.66 (0.25-1.70) | 0.386 | 0.41 (0.18-1.35) | 0.145 |
| Enrollment within 48h from sepsis onset | 23 (39.7) | 19 (36.5) | 0.88 (0.41-1.89) | 0.737 | 0.42 (0.14-1.29) | 0.133 |

Abbreviations SOFA: sequential organ failure assessment

**Table S6. Post-hoc regression analysis for variables associated with 28-day mortality, exploring different empirical antimicrobial treatment combinations (more than two antimicrobials -excluding antifungals)**

|  | **Survivors (n=58)** | **Non-survivors (n=52)** | **Unadjusted OR (95%CI)** | **P-value** | **Adjusted OR (95%CI)** | **P-value** |
| --- | --- | --- | --- | --- | --- | --- |
| Clarithromycin | 28 (48.3) | 27 (51.9) | 1.16 (0.55-2.45) | 0.849 | 1.39 (0.61-3.17) | 0.431 |
| Charlson Comorbidity Index | 4 (2-6) | 7 (5-8) | 1.34 (1.14-1.58) | **3.36x10^-4^** | 1.34 (1.143-1.58) | **3.36x10^-4^** |
| SOFA score on day 1 | 10 (9-12) | 11 (9-13) | 1.12 (0.95-1.32) | 0.161 | 1.08 (0.90-1.29) | 0.418 |
| ≥2 antimicrobials (excluding antifungals) | 21 (36.2) | 22 (42.3) | 1.29 (0.60-2.78) | 0.513 | 1.73 (0.74-4.07) | 0.208 |

Abbreviations SOFA: sequential organ failure assessment

**Table S7. Post-hoc regression analysis for variables associated with 28-day mortality, exploring different empirical antimicrobial treatment combinations (more than two antimicrobials including antifungals)**

|  | **Survivors (n=58)** | **Non-survivors (n=52)** | **Unadjusted OR (95%CI)** | **P-value** | **Adjusted OR (95%CI)** | **P-value** |
| --- | --- | --- | --- | --- | --- | --- |
| Clarithromycin | 28 (48.3) | 27 (51.9) | 1.18 (0.55-2.45) | 0.849 | 1.42 (0.62-3.26) | 0.406 |
| Charlson Comorbidity Index | 4 (2-6) | 7 (5-8) | 1.348 (1.14-1.58) | **3.36x10^-4^** | 1.36 (1.15-1.61) | **2.77x10^-4^** |
| SOFA score on day 1 | 10 (9-12) | 11 (9-13) | 1.128 (0.95-1.32) | 0.161 | 1.08 (0.90-1.29) | 0.403 |
| ≥2 antimicrobials (including antifungals) | 29 (50.0) | 33 (63.5) | 1.74 (0.81-3.73) | 0.157 | 2.01 (0.87-4.62) | 0.102 |

Abbreviations SOFA: sequential organ failure assessment

**Table S8. Post-hoc regression analysis for variables associated with 28-day mortality, exploring different empirical antimicrobial treatment combinations (Gram-positive coverage)**

|  | **Survivors (n=58)** | **Non-survivors (n=52)** | **Unadjusted OR (95%CI)** | **P-value** | **Adjusted OR (95%CI)** | **P-value** |
| --- | --- | --- | --- | --- | --- | --- |
| Clarithromycin | 28 (48.3) | 27 (51.9) | 1.18 (0.55-2.45) | 0.849 | 1.38 (0.61-3.14) | 0.444 |
| Charlson Comorbidity Index | 4 (2-6) | 7 (5-8) | 1.34 (1.14-1.58) | **3.36x10^-4^** | 1.34 (1.14-1.58) | **3.36x10^-4^** |
| SOFA score on day 1 | 10 (9-12) | 11 (9-13) | 1.12 (0.95-1.32) | 0.161 | 1.10 (0.92-1.31) | 0.284 |
| Gram-positive coverage | 31 (53.4) | 23 (44.2) | 0.69 (0.33-1.47) | 0.335 | 0.69 (0.31-1.57) | 0.380 |

Abbreviations SOFA: sequential organ failure assessment

**Table S10. Post-hoc regression analysis for variables associated with 28-day mortality, exploring different empirical antimicrobial treatment combinations (colistin combinations)**

|  | **Survivors (n=58)** | **Non-survivors (n=52)** | **Unadjusted OR (95%CI)** | **P-value** | **Adjusted OR (95%CI)** | **P-value** |
| --- | --- | --- | --- | --- | --- | --- |
| Clarithromycin | 28 (48.3) | 27 (51.9) | 1.18 (0.55-2.45) | 0.849 | 1.44 (0.41-5.02) | 0.581 |
| Charlson Comorbidity Index | 4 (2-6) | 7 (5-8) | 1.34 (1.14-1.58) | **3.36x10^-4^** | 1.43 (1.11-1.85) | **0.006** |
| SOFA score on day 1 | 10 (9-12) | 11 (9-13) | 1.12 (0.95-1.32) | 0.161 | 1.12 (0.87-1.44) | 0.381 |
| Intake of colistin | 26 (44.9) | 25 (48.1) | 1.14 (0.54-2.42) | 0.733 | 0.85 (0.15-4.67) | 0.851 |

Abbreviations SOFA: sequential organ failure assessment

**Table S11. Type and pathogen of first sepsis episode recurrence by day 28 or discharge, among patients with sepsis response by day 7**

| **Site** | **Pathogen, n (%)** | **Clarithromycin (n=7)** | **Placebo (n=19)** | **P-value** | **Recurrence (n=13)** |
| --- | --- | --- | --- | --- | --- |
| Pneumonia (n=10) | All | 4 (57.1) | 6 (31.6) | 0.369 | 9 (69.2) |
|  | *Acinetobacter baumannii* | 0 (0.0) | 1 (5.3) | 1.00 |  |
|  | *Klebiella pneumoniae* | 0 (0.0) | 1 (5.3) | 1.00 |  |
|  | *Pseudomonas aeruginosa* | 0 (0.0) | 2 (10.5) | 1.00 |  |
|  | *Staphylococcus aureus* | 0 (0.0) | 1 (5.3) | 1.00 |  |
|  | Other^a^ | 2 (28.6) | 0 (0.0) | 0.065 |  |
|  | Non-identified | 2 (28.6) | 1 (5.3) | 0.167 |  |
| Intra-abdominal infection (n=4) | All | 2 (28.6) | 2 (10.5) | 0.546 | 3 (75.0) |
|  | *Acinetobacter baumannii* | 0 (0.0) | 1 (5.3) | 1.00 |  |
|  | *Pseudomonas aeruginosa* | 1 (14.3) | 0 (0.0) | 0.269 |  |
|  | Non-identified | 1 (14.3) | 1 (5.3) | 0.473 |  |
| Primary Gram (-) bacteremia (n=6) | All | 1 (14.3) | 5 (26.3) | 0.494 | 1 (7.7) |
|  | *Acinetobacter baumannii* | 0 (0.0) | 2 (10.5) | 1.00 |  |
|  | *Klebiella pneumoniae* | 1 (14.3) | 1 (5.3) | 0.473 |  |
|  | *Pseudomonas aeruginosa* | 0 (0.0) | 1 (5.3) | 1.00 |  |
|  | *Providentia stuartii* | 0 (0.0) | 1 (5.3) | 1.00 |  |
| CRBSI (n=2) | All | 0 (0.0) | 2 (10.5) | 1.00 | 0 (0.0) |
|  | *Acinetobacter baumannii* | 0 (0.0) | 1 (5.3) | 1.00 |  |
|  | *Staphylococcus aureus* | 0 (0.0) | 1 (5.3) | 1.00 |  |
| Primary fungemia (n=2) | All | 0 (0.0) | 2 (10.5) | 1.00 | 0 (0.0) |
|  | *Candida parapsilosis* | 0 (0.0) | 1 (5.3) | 1.00 |  |
|  | *Trichosporon asahii* | 0 (0.0) | 1 (5.3) | 1.00 |  |
| UTI (n=1) | *Morganella morgannii* | 0 (0.0) | 1 (5.3) | 1.00 | 0 (0.0) |
| ABSSSI (n=1) | *Serratia marcescens* | 0 (0.0) | 1 (5.3) | 1.00 | 0 (0.0) |
| All (n=26) |  | 7 (100) | 19 (100) |  |  |
|  | *Acinetobacter baumannii* | 0 (0.0) | 5 (26.3) | 0.278 |  |
|  | *Klebiella pneumoniae* | 1 (14.3) | 2 (10.5) | 1.00 |  |
|  | *Pseudomonas aeruginosa* | 1 (14.3) | 3 (15.8) | 1.00 |  |
|  | *Staphylococcus aureus* | 0 (0.0) | 2 (10.5) | 1.00 |  |
|  | Fungi | 0 (0.0) | 2 (10.5) | 1.00 |  |
|  | Other^b^ | 2 (28.6) | 3 (15.8) | 0.586 |  |
|  | Non-identified | 3 (42.9) | 2 (10.5) | 0.101 |  |

^a^*Legionella non-pneumophila* and *Serratia marcescens*

^b^Including *Legionella non-pneumophila*, *Serratia marcescens*, *Providentia stuartii*, *Morganella morgannii*

Abbreviations ABSSSI: acute bacterial skin and soft structure infections; CRBSI: catheter-related bloodstream infections; UTI: urinary tract infections

**Table S12. Sites of first episode sepsis recurrence, compared to initial site of infection, among patients with sepsis response by day 7**

|  |  | **Original sepsis focus, n (%)** | | |  |
| --- | --- | --- | --- | --- | --- |
|  |  | **Pneumonia**  **(n=22)** | **Intra-abdominal infection (n=3)** | **Primary Gram (-) bacteremia (n=1)** | **P-value** |
| **First**  **Sepsis**  **Recurrence**  **Focus** | Pneumonia (n=10) | 9 (40.9) | 1 (33.3) | 0 (0.0) | 0.073 |
|  | Intra-abdominal infection (n=4) | 2 (9.0) | 2 (66.7) | 0 (0.0) |  |
|  | Primary Gram-negative bacteremia (n=6) | 6 (27.3) | 0 (0.0) | 0 (0.0) |  |
|  | CRBSI (n=2) | 1 (4.6) | 0 (0.0) | 1 (100.0) |  |
|  | Primary fungemia (n=2) | 2 (9.0) | 0 (0.0) | 0 (0.0) |  |
|  | UTI (n=1) | 1 (4.6) | 0 (0.0) | 0 (0.0) |  |
|  | ABSSSI (n=1) | 1 (4.6) | 0 (0.0) | 0 (0.0) |  |

Abbreviations ABSSSI: acute bacterial skin and soft structure infections; CRBSI: catheter-related bloodstream infections; UTI: urinary tract infections

**Table S13. Description of the eight patients who presented with Acute Kidney Injury**

| **AKI case #** | **Treatment arm** | **Baseline creatinine (mg/dl)** | **Creatinine on AKI onset**  **(mg/dl)** | **Creatinine peak (mg/dl)** | **Days from enrollment** | **Cause of AKI** |
| --- | --- | --- | --- | --- | --- | --- |
| 1 | Clarithromycin | 1.40 | 1.80 | 3.40 | 13 | Vancomycin-associated |
| 2 | Placebo | 1.10 | 1.20 | 2.00 | 4 | Progression into shock/MODS |
| 3 | Clarithromycin | 0.80 | 1.20 | 1.20 | 3 | Progression into shock/MODS |
| 4 | Clarithromycin | 0.60 | 1.20 | 1.60 | 16 | Prerenal AKI due to diarrhea |
| 5 | Clarithromycin | 2.90 | 3.30 | 3.40 | 7 | Contrast-induced tubulopathy |
| 6 | Clarithromycin | 1.90 | 1.56 | 1.56 | 5 | Post-operative AKI |
| 7 | Clarithromycin | 2.32 | 4.93 | 4.93 | 6 | Pre-existing CRD; need for CRRT |
| 8 | Clarithromycin | 0.83 | 1.20 | 1.60 | 13 | New shock onset |

Abbreviations AKI: acute kidney injury; CRD: chronic renal disease; CRRT: continuous renal replacement therapy; MODS: multiple organ dysfunction syndrome; SAE: serious adverse event

**Table S14. Non-serious adverse events**

|  | **Clarithromycin (n=55)** | **Placebo (n=55)** | **P-value** |
| --- | --- | --- | --- |
| Patients with at least one AE, n (%) | 48 (87.3) | 48 (87.3) | 1.00 |
| Blood and lymphatic system disorders, n (%) | 23 (41.8) | 19 (34.5) | 0.556 |
| Psychiatric disorders, n (%) | 7 (12.7) | 10 (18.2) | 0.599 |
| Neurological disorders, n (%) | 6 (10.9) | 4 (7.3) | 0.742 |
| Cardiac arrythmias, n (%) | 14 (25.5) | 14 (25.5) | 1.00 |
| Vascular disorders, n (%) | 16 (29.1) | 14 (25.5) | 0.831 |
| Respiratory, thoracic or mediastinal disorders, n (%) | 3 (5.5) | 3 (5.5) | 1.00 |
| Gastro-intestinal disorders, n (%) | 26 (47.3) | 29 (52.7) | 0.703 |
| Transminases increased, n (%) | 27 (49.1) | 24 (43.6) | 0.702 |
| Skin (allergic) and subcutaneous tissue (decubitus ulcer) disorders, n (%) | 12 (21.8) | 12 (21.8) | 1.00 |
| Musculo-skeletal disorders (myopathy), n (%) | 8 (14.5) | 14 (25.5) | 0.233 |
| Metabolism and nutrition disorders, n (%) | 33 (60.0) | 36 (65.5) | 0.694 |

Percentages may not add up to 100% since patients have experienced more than one non-serious adverse event.

**
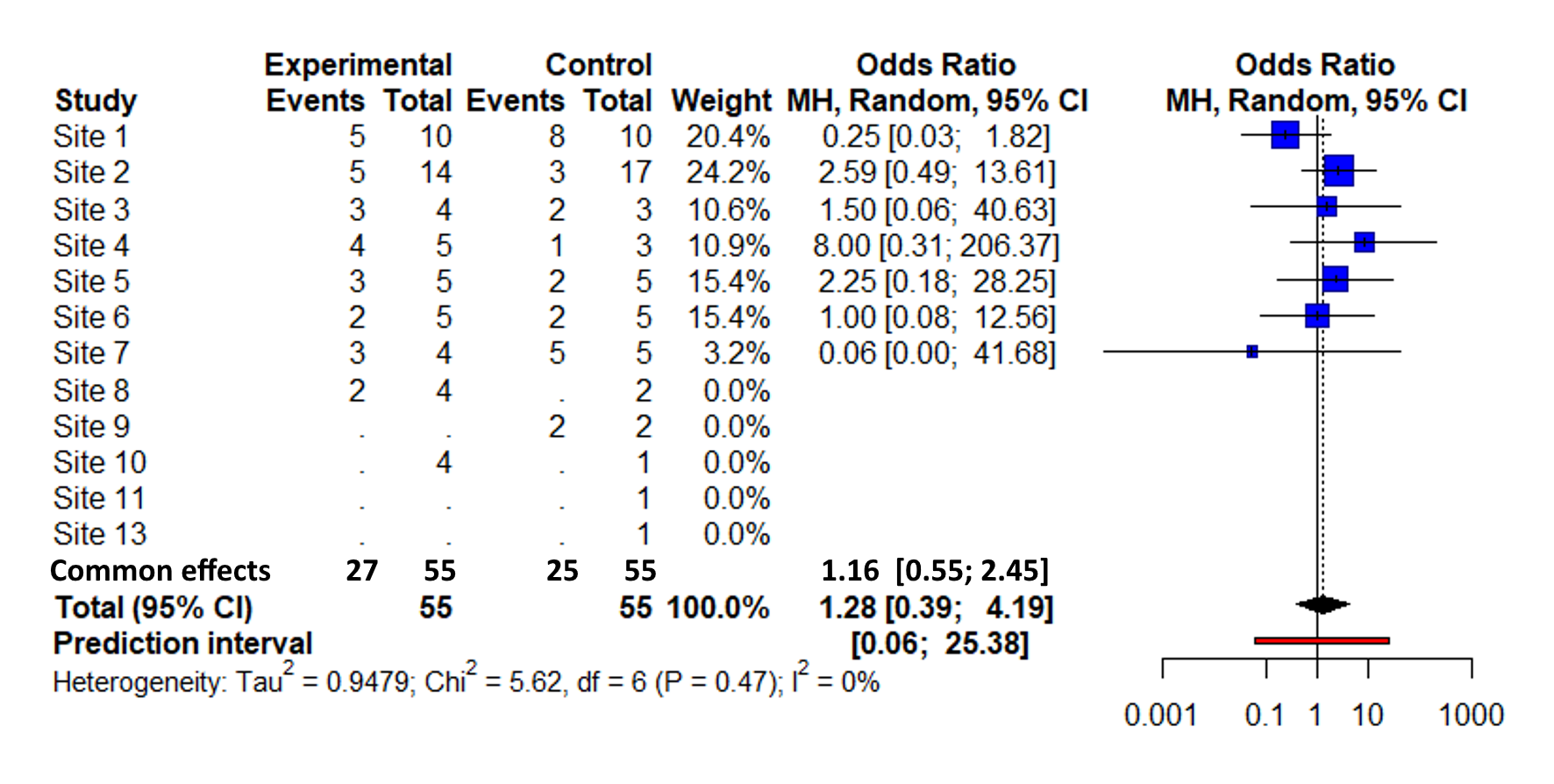
**

**Figure S1. Forest plot of study arm effect on 28-day mortality, stratified by study site.**

**
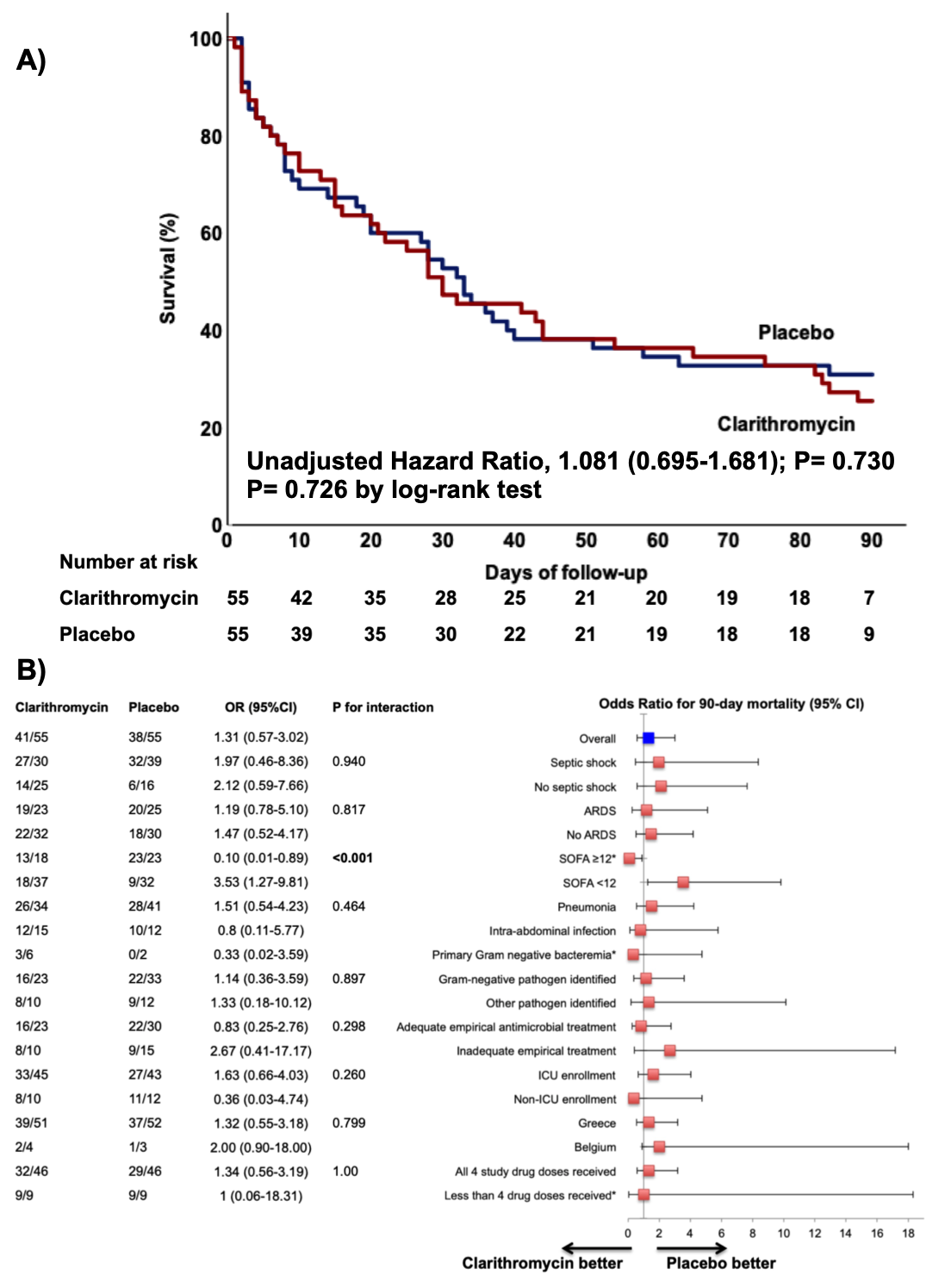
**

**Figure S2. 90-day survival among trial participants**

A) 90-day survival analysis by Kaplan-Meier curves among patients with sepsis and multiple organ dysfunction syndrome, treated with Clarithromycin or Placebo. Hazard Ratio is provided by Cox-regression analysis B) Risk of death within 90-days in pre-specified subgroups among patients treated with clarithromycin or placebo. *P*-values for interactions between treatment arm and subgroup are provided by the Breslow-Day test.

*Calculated using the Firth correction

Abbreviations ARDS: acute respiratory distress syndrome; CI: confidence intervals; ICU: intensive care unit; SOFA: sequential organ failure assessment

**
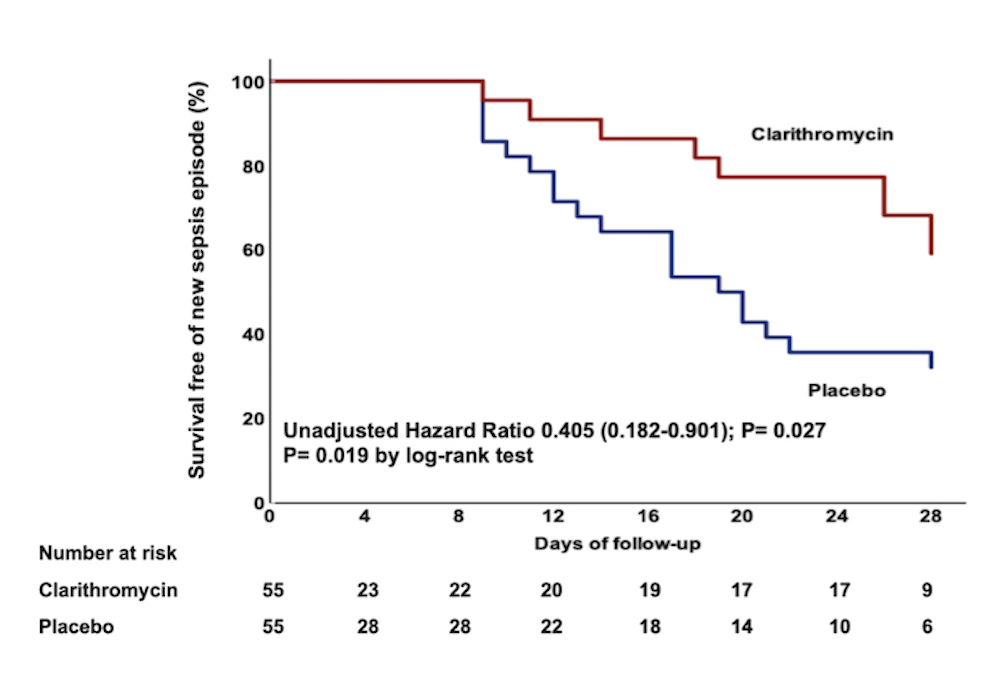
**

**Figure S3. Survival-free time from new sepsis episode among clarithromycin or placebo-treated patients.** Hazard Ratio is provided by Cox-regression analysis.

**
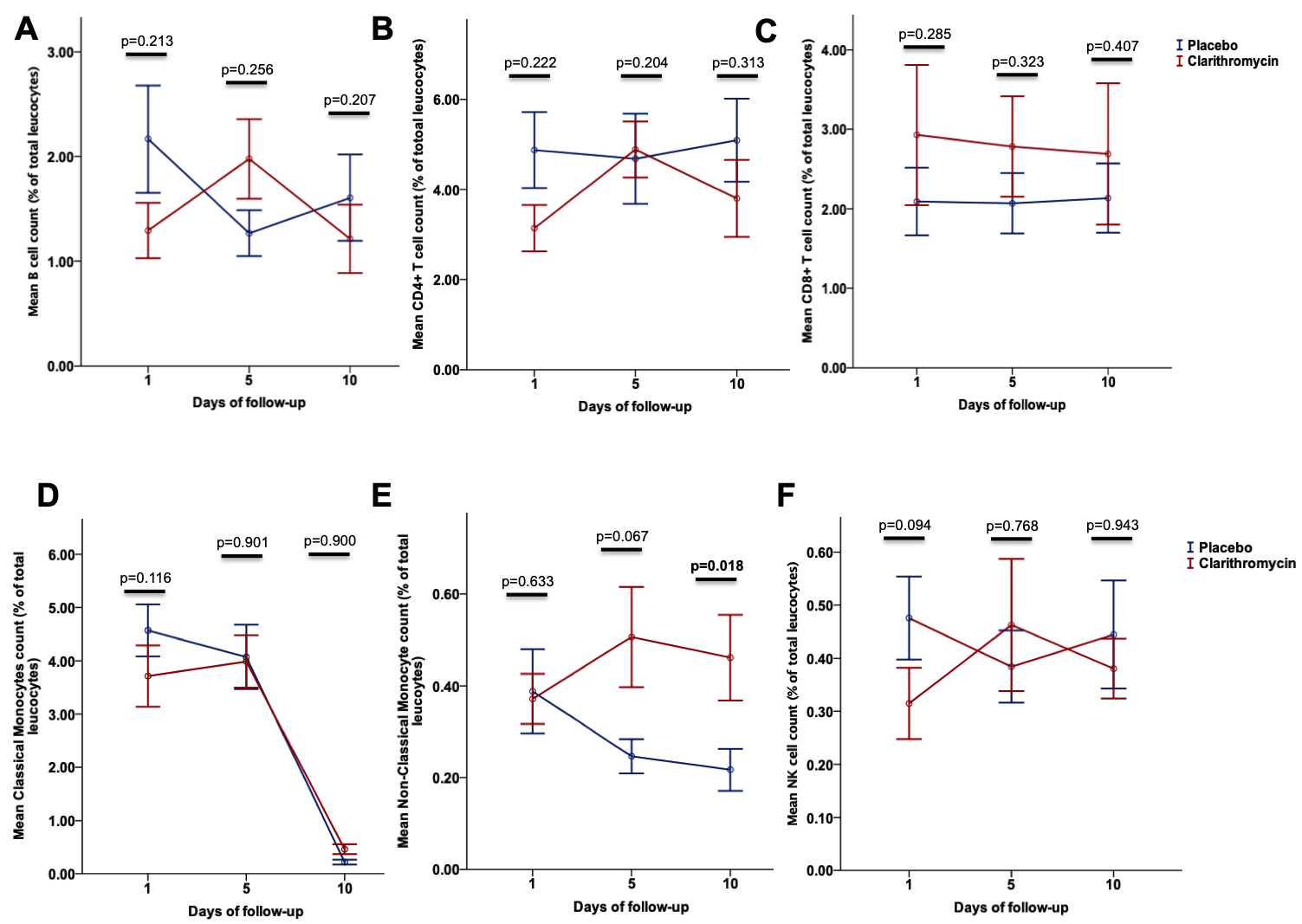
**

**Figure S4. Cell population analysis among clarithromycin and placebo-treated patients.** Results are presented for days 1 (baseline), 5 and 10 after enrollment.

A) B cells, B) CD4^+^ T cells, C) CD8^+^ T cells, D) classical monocytes, E) non-classical monocytes and F) natural killer cells expressed as the percentage of total leukocytes. Lines represent mean values with respective standard error (SE). A total of n= 42 (21 clarithromycin and 21 placebo-treated), 45 (22 clarithromycin and 23 placebo-treated) and 33 (16 clarithromycin and 17 placebo-treated) patient samples contributed to the analysis of each of days 1, 5 and 10, respectively. P values for comparisons by the Mann-Whitney test are provided.

**
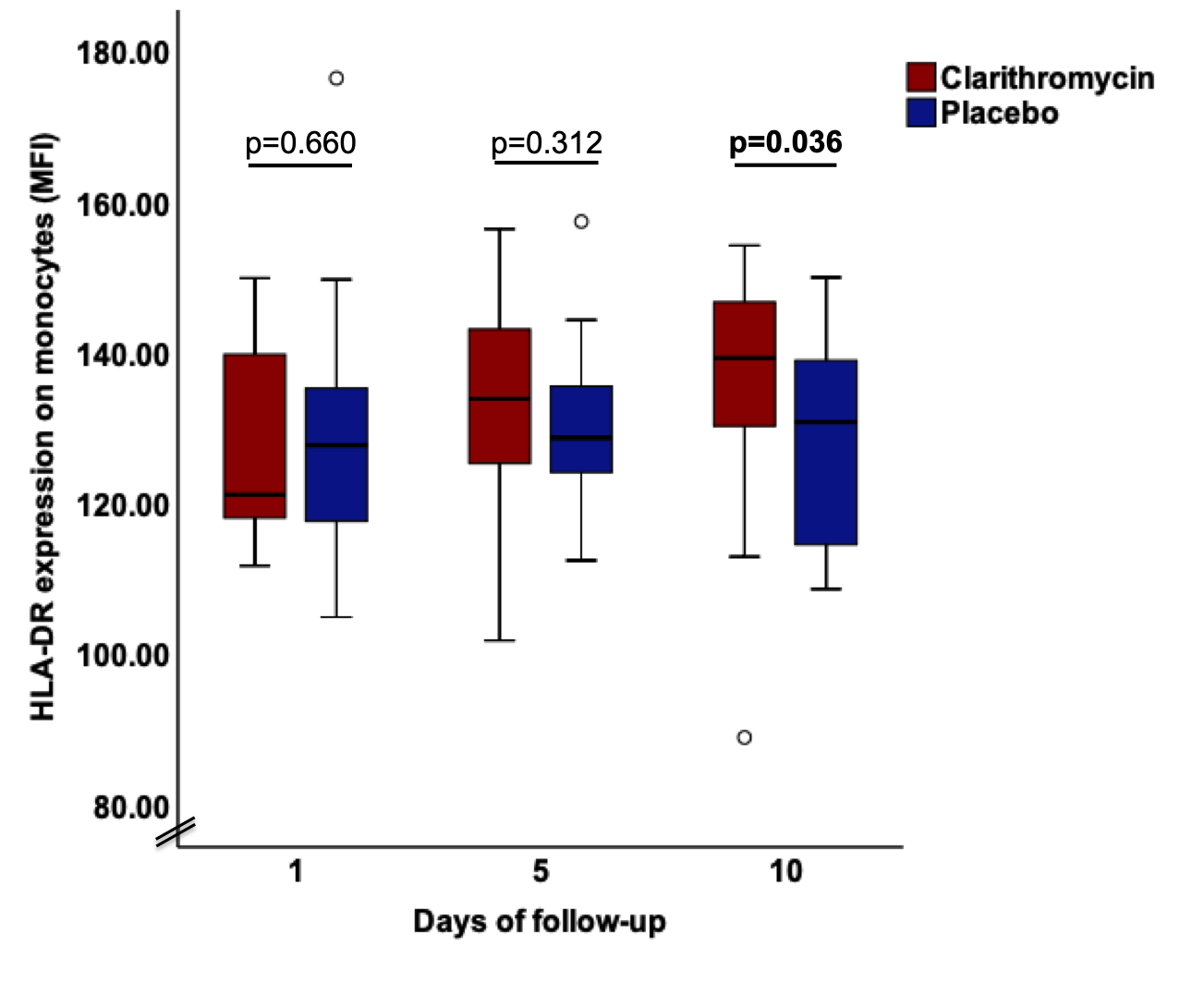
**

**Figure S5. HLA-DR expression on monocytes among clarithromycin and placebo-treated patients.** Results are presented as mean fluorescent intensity (MFI) for days 1 (baseline), 5 and 10 after enrolment.

Boxes represent median values with higher and lower quartiles, and whiskers depict maximum and minimum values. A total of n= 49 (25 clarithromycin and 24 placebo-treated), 46 (22 clarithromycin and 24 placebo-treated) and 35 (17 clarithromycin and 18 placebo-treated) patient samples contributed on days 1, 5 and 10, respectively. P-values for comparisons by the Mann-Whitney test are provided

**
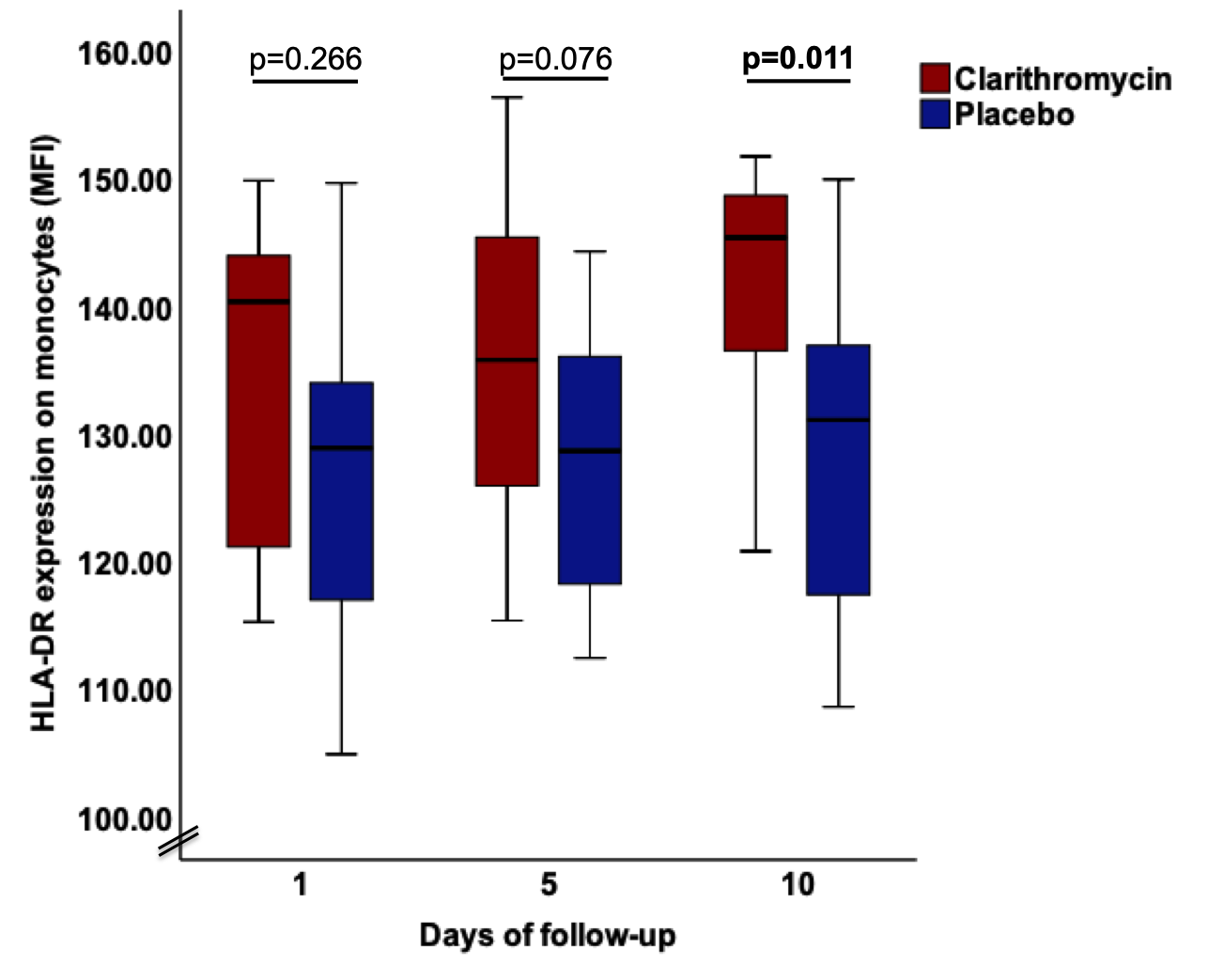
**

**Figure S6. HLA-DR expression on monocytes among clarithromycin and placebo-treated patients with sepsis response by day 7 after enrollment.** Results are expressed as mean fluorescent intensity (MFI) and presented for days 1 (baseline), 5 and 10 after enrolment.

Boxes represent median values with higher and lower quartiles, and whiskers depict maximum and minimum values. A total of n= 23 (9 clarithromycin and 14 placebo-treated), 27 (11 clarithromycin and 16 placebo-treated) and 30 (11 clarithromycin and 19 placebo-treated) patient samples contributed on days 1, 5 and 10, respectively. P-values for comparisons by the Mann-Whitney test are provided

**
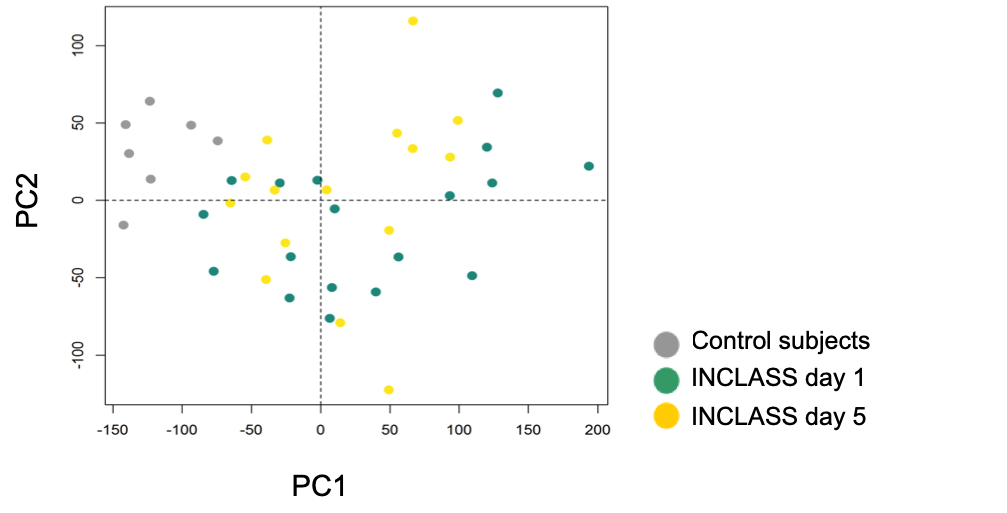
**

**Figure S7. Principal component (PC) analysis** of the expressed transcripts (n=31,710) among patients enrolled in the INCLASS study (n=18) and healthy age-, sex- and Charlson’s Comorbidity Index-matched control subjects (n=7).

**
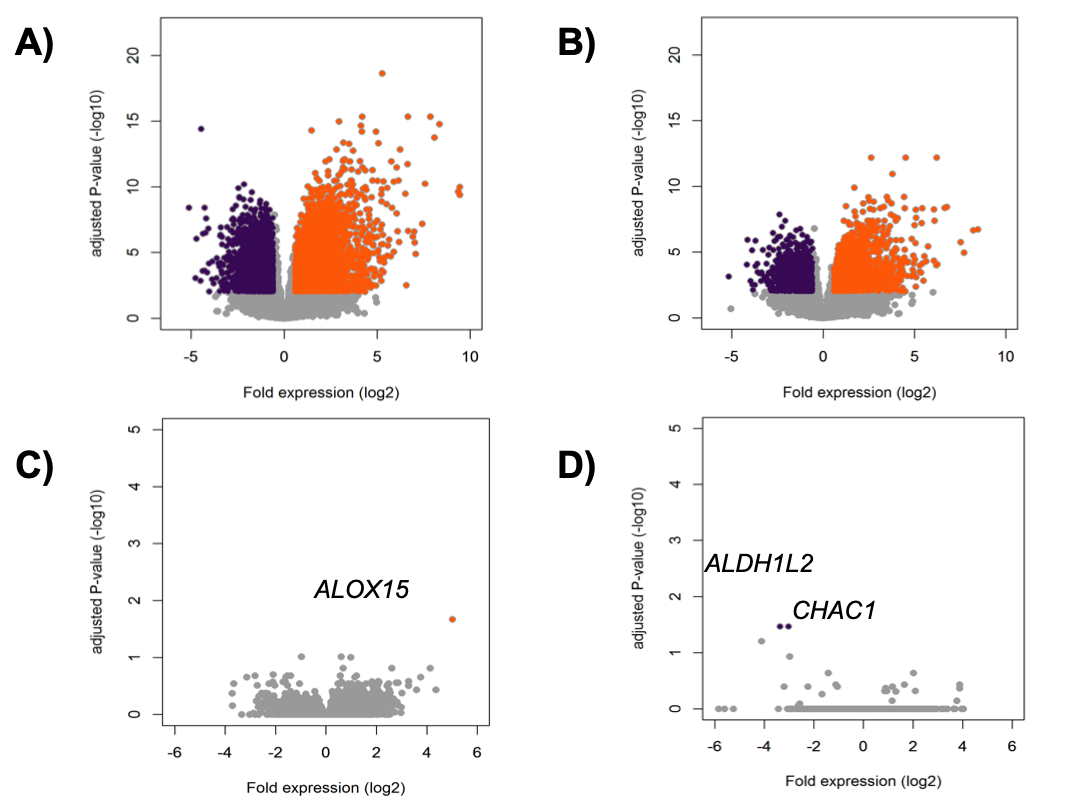
**

**Figure S8. Transcriptomic profiling of leukocytes.** Panels A and B are Volcano plot representations (integrating log2 fold change and multiple-comparison adjusted p values) of genome- wide alterations in RNA expression of INCLASS patient leukocytes obtained on days 1 and 5 respectively relative to control leukocytes. Panels C and D indicate the most important up-regulated and down-regulated genes on days 1 and 5 respectively relative to placebo-treated patients’ leukocytes. Upregulated genes represent absolute fold expression >1.2 and are adjusted for *P*<0.01; downregulated genes represent absolute fold expression <1.2 and are adjusted for *P*<0.01.

**
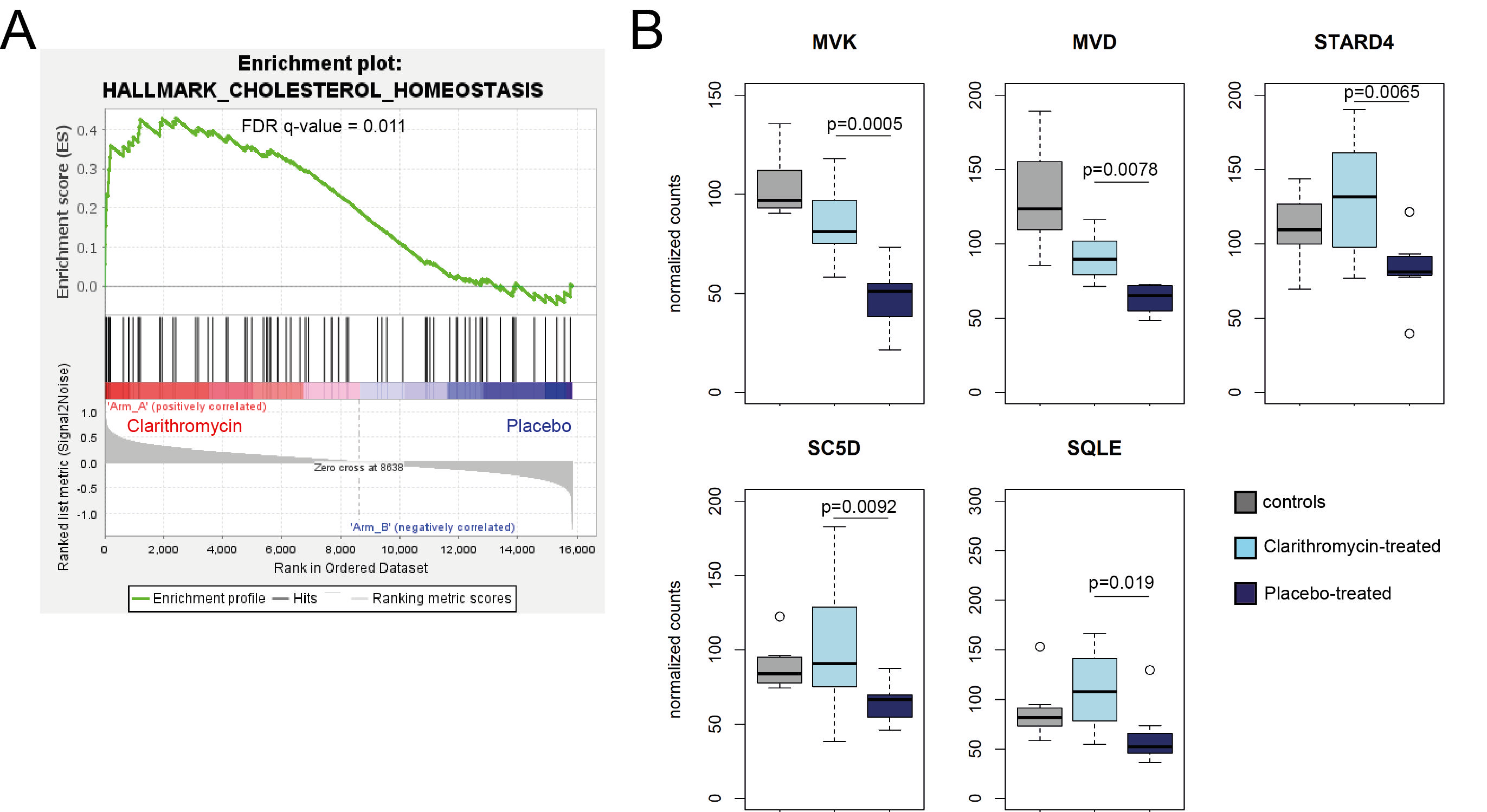
**

**Figure S9. Cholesterol biosynthesis pathways** **among patients treated with clarithromycin or placebo.** A) Gene Set Enrichment Analysis (GSEA) of clarithromycin (red colour) minus placebo (blue colour) arm, on day 5 of follow-up. An adjusted *P*< 0.05 was used to control false-discovery-rate and demarcated significantly enriched gene sets.

B) Box-plots of normalized counts of the main genes involved in cholesterol biosynthesis pathway (*MVK, SC5D, MVD, STARD4* and *SQLE*) among healthy control subjects (grey color), clarithromycin-treated patients (light blue color) and placebo-treated patients (dark blue color) on day 5 of follow-up. Boxes represent median, upper and lower quartiles of DESeq2 normalized counts of transcripts, while maximum and minimum values are depicted as whiskers. *P* values of comparisons between clarithromycin and placebo-treated patients are shown.

Abbreviations *MVK:* Mevalonate Kinase; *SQLE:* Squalene Epoxidase; *SC5D:* Sterol-C5-Desaturase; *MVD:* Mevalonate Diphosphate Decarboxylase; *STARD4:* StAR Related Lipid Transfer Domain Containing 4.

**References:**

1. Rhodes A, Evans LE, Alhazzani W, Levy MM, Antonelli M, Ferrer R, et al. Surviving sepsis campaign: international guidelines for management of sepsis and septic shock: 2016. Crit Care Med. 2017;45:486-552.
2. Ελληνική Ομάδα Μελέτης της Σήψης. Ενημερωτικό Δελτίο για τη Σήψη. Αθήνα 2017. <http://sepsis.gr/dmsepsis/wp-content/uploads/2019/04/ΕΝΗΜΕΡΩΤΙΚΟ-ΔΕΛΤΙΟ.pdf>. Accessed 04 Feb 2022
3. Ελληνική Εταιρεία Χημειοθεραπείας. Η Ορθολογική Επιλογή Αντιμικροβιακής Θεραπείας για το Νοσηλευόμενο Ασθενή. Αθήνα 2017. <https://eex.org.gr/wp-content/uploads/2019/07/guide-2017-eex.pdf>. Accessed 04 Feb 2022
4. Belgian Antibiotic Policy Coordination Committee. Recommandations de traitements anti-infectieux en milieu hospitalier, élaborées par la SBIMC. 2017. <https://organesdeconcertation.sante.belgique.be/sites/default/files/documents/bapcoc_guidelineshospi_2017_sbimc-bvikm_fr_v1.pdf>. Accessed 04 Feb 2022
5. Giamarellos-Bourboulis EJ, Mylona V, Antonopoulou A, Tsangaris I, Koutelidakis I, Marioli A, et al. Effect of clarithromycin in patients with suspected Gram-negative sepsis: results of a randomized controlled trial. J Antimicrob Chemother. 2014;69:1111-8.
6. Giamarellos-Bourboulis EJ, Pechère JC, Routsi C, Plachouras D, Kollias S, Raftogiannis M, et al. Effect of clarithromycin in patients with sepsis and ventilator-associated pneumonia. Clin Infect Dis. 2008;46:1157-64.
7. Singer M, Deutschman CS, Seymour CW, Shankar-Hari M, Annane D, Bauer M, et al. The Third International Consensus Definitions for Sepsis and Septic Shock (Sepsis-3). JAMA. 2016;315:801-810.
8. Kalil AC, Metersky ML, Klompas M, Muscedere J, Sweeney DA, Palmer LB, et al. Management of adults with hospital-acquired and ventilator-associated pneumonia: 2016 clinical practice guidelines by the infectious diseases society of America and the American thoracic society. Clin Infect Dis. 2016;63:e61-e111.
9. Calandra T, Cohen J. International sepsis forum definition of infection in the ICU consensus conference. The international sepsis forum consensus conference on definitions of infection in the intensive care unit. Crit Care Med. 2005;33:1538-48.
10. Bennet JE, Dolin R, Blaser MJ. Mandell, Douglas and Bennett's Principles and practice of infectious diseases.  Philadelphia, PA: Elsevier/Saunders; 2015.
11. ARDS Definition Task Force, Ranieri VM, Rubenfeld GD, Thompson BT, Ferguson ND, Caldwell E, Fan E, et al. Acute respiratory distress syndrome: the Berlin Definition. JAMA. 2012;307:2526-33.
12. Karakike E, Kyriazopoulou E, Tsangaris I, Routsi C, Vincent JL, Giamarellos-Bourboulis EJ. The early change of SOFA score as a prognostic marker of 28-day sepsis mortality: analysis through a derivation and a validation cohort. Crit Care. 2019;23:387.
13. Severinghaus JW. Simple, accurate equations for human blood O2 dissociation computations. J Appl Physiol Respir Environ Exerc Physiol 1979; 46:599-602.
14. Bolger AM, Lohse M, Usadel B. Trimmomatic: a flexible trimmer for Illumina sequence data. Bioinformatics. 2014;30:2114-2120.
15. Harrow J, Denoeud F, Frankish A, Reymond A, Chen CK, Chrast J, et al. GENCODE: producing a reference annotation for ENCODE. Genome Biol. 2006;7 (S4): 1-9.
16. Kim D, Langmead B, Salzberg SL. HISAT: a fast spliced aligner with low memory requirements. Nat Methods. 2015;12:357-360.
17. Anders S, Pyl PT, Huber W. HTSeq--a Python framework to work with high-throughput sequencing data. Bioinformatics. 2015;31:166-169.
18. Love MI, Huber W, Anders S. Moderated estimation of fold change and dispersion for RNA-seq data with DESeq2. Genome Biol. 2014;15:550.
19. Subramanian A, Tamayo P, Mootha VK, Mukherjee S, Ebert BL, Gillette MA, et al. Gene set enrichment analysis: A knowledge-based approach for interpreting genome-wide expression profiles. Proc Natl Acad Sci U S A. 2005;102:15545-15550.
