# Supplementary material for: Effect of intravenous clarithromycin in patients with sepsis, respiratory and multiple organ dysfunction syndrome: a randomized clinical trial
Source: Crit Care. 2022 Jun 18;26:183. doi: 10.1186/s13054-022-04055-4 (PMC9206755; doi:10.1186/s13054-022-04055-4)
Supplement: Supplementary file 2 — Additional file 2. Contains the complete Study Protocol versions 1 and 2, a detailed description of amendments between protocol versions and the Statistical Analysis Plan. [file 13054_2022_4055_MOESM2_ESM.docx]

**Additional file 2**

**EFFECT OF INTRAVENOUS CLARITHROMYCIN IN PATIENTS WITH SEPSIS, RESPIRATORY AND MULTIPLE ORGAN DYSFUNCTION SYNDROME: A RANDOMIZED CLINICAL TRIAL**

**Running Title: Clarithromycin as immune modulator (INCLASS trial)**

Eleni Karakike, MD, Brendon P. Scicluna, PhD, Maria Roumpoutsou, MD^*^,

Ioannis Mitrou, MD^*^, Niki Karampela, MD, Athanasios Karageorgos, MSc,

Konstantinos Psaroulis, MD, PhD, Eleni Massa, MD, Achillefs Pitsoulis, MD,

Panagiotis Chaloulis, MD, Evanthia Pappa, MD, Irene T. Schrijver, MD, PhD,

Frantzeska Frantzeskaki, MD, PhD, Malvina Lada, MD, PhD,

Nicolas Dauby, MD, PhD, David De Bels, MD, PhD, Ioannis Floros, MD, PhD,

Souzana Anisoglou, MD, PhD, Eleni Antoniadou, MD, PhD, Maria Patrani, MD, PhD, Glykeria Vlachogianni, MD, PhD, Eleni Mouloudi, MD, PhD,

Anastasia Antoniadou, MD, PhD, David Grimaldi, MD, PhD, Thierry Roger, PhD,

W. Joost Wiersinga, MD, PhD, Iraklis Tsangaris, MD, PhD,

Evangelos J. Giamarellos-Bourboulis, MD, PhD

^*^equal contribution

**TABLE OF CONTENTS**

1. Protocol version 1 (Original version) 2

2. Protocol version 2 (Final version) 30

3. Details of amendments to the original protocol 66

4. Statistical Analysis Plan 78

# 1. Protocol version 1 (Original version)

**A DOUBLE-BLIND, RANDOMIZED, PLACEBO-CONTROLLED CLINICAL STUDY OF THE EFFICACY OF INTRAVENOUS CLARITHROMYCIN AS ADJUNCTIVE TREATMENT IN PATIENTS WITH SEPSIS AND RESPIRATORY AND MULTIPLE ORGAN DYSFUNCTION SYNDROME**

**Running title: INtravenous CLArithromycin in Sepsis and Multiple Organ Dysfunction Syndrome (INCLASS study)**

**CLINICAL STUDY PROTOCOL**

**Authors:**

**Eleni Karakike, MD and Evangelos J. Giamarellos-Bourboulis, MD, PhD**

**HELLENIC INSTITUTE FOR THE STUDY OF SEPSIS, GREECE**

**Protocol version:** 1

**Protocol date:** 05 January 2017

**EudraCT number:** 2017-001056-55

**ATHENS 2017**

**TABLE OF CONTENTS**

|  | Page |
| --- | --- |
| **Disclosure of Principal Investigator** | **3** |
| **Abbreviations** | **4** |
| **Study Synopsis** | **5** |
| **Introduction** | **8** |
| **Aim of the study** | **10** |
| **Study design** | **10** |
| **Type of study and study sites** | **10** |
| **Inclusion criteria** | **10** |
| **Exclusion criteria** | **11** |
| **Definitions** | **11** |
| **Study drug preparation and administration** | **13** |
| **Study visits** | **14** |
| **Laboratory analysis** | **19** |
| **Study endpoints** | **19** |
| **Study power calculation** | **20** |
| **Statistical analysis** | **21** |
| **Duration of the study** | **21** |
| **Adverse events** | **21** |
| **References** | **24** |
| **Appendix I** | **26** |
| **Appendix II** | **27** |
| **Appendix III** | **28** |

**DISCLOSURE OF PRINCIPAL INVESTIGATOR**

**Protocol Study Title:** A DOUBLE-BLIND, RANDOMIZED, PLACEBO-CONTROLLED CLINICAL STUDY OF THE EFFICACY OF INTRAVENOUS CLARITHROMYCIN AS ADJUNCTIVE TREATMENT IN PATIENTS WITH SEPSIS AND RESPIRATORY AND MULTIPLE ORGAN DYSFUNCTION SYNDROME.

The herein protocol became known to myself by the Study Sponsor. I understand that the protocol remains as yet unpublished; I certify that all disclosed information to myself for this protocol will remain strictly confidential.

The Principal Investigator,

Print Name

Signature Date

**ABBREVIATIONS**

APACHE: acute physiology and chronic health evaluation

ARDS: acute respiratory distress syndrome

CCI: Charlson Comorbidity Index

HAP: hospital-acquired pneumonia

HCAP: health care-associated pneumonia

VAP: ventilator-associated pneumonia

HIV: human immunodeficiency virus

IAI: intra-abdominal infection

BSI: primary Gram-negative bacteremia

CD4: T4 lymphocytes

COPD: chronic obstructive pulmonary disease

EDTA: ethylenediamine actic acid

FiO_2_: fraction of inspired oxygen

G: grammar

ICU: intensive care unit

IL: interleukin

MODS: multiple organ dysfunction syndrome

OR: odds ratio

PaO_2_: partial arterial oxygen pressure

RCT: randomized clinical trial

SOFA: sequential organ failure assessment

TNFα: tumour necrosis factor-alpha

AE: adverse event

SAE: serious adverse event

PBMCs: Peripheral Blood Mononuclear Cells

**STUDY SYNOPSIS**

| **Background** | High mortality associated with sepsis and MODS calls for alternative, individualized therapies in selected patients that might benefit form specific interventions. Role of macrolides as potential immunomodulatory treatment in sepsis is promising, but unclear. Subgroup analysis of previous large-scale clinical trials on patients with ventilator-associated pneumonia or gram-negative sepsis, showed that addition of clarithromycin to standard antibiotic therapy conferred a significant survival benefit in the subgroup of patients with respiratory dysfunction and MODS, but this effect has never been investigated through randomized controlled trials in an entire population suffering from these entities. |
| --- | --- |
| **Aim** | The study is aiming to assess the efficacy of intravenous treatment of clarithromycin in the reduction of 28-day mortality among patients with sepsis and respiratory dysfunction. Secondary aims are the effect on 90-day mortality, sepsis resolution, and recurrence, mortality in the subgroup of patients with septic shock and finally, impact on biomarkers of sepsis-induced immunosuppression |
| **Design** | Multicenter, interventional, double blind, randomized, placebo-controlled phase IV study |
| **Inclusion criteria** | 1. Adult patients (≥18 years) 2. Patients of both genders 3. Informed consent form signed by patient or by first-degree relative in case of patient unable to consent 4. Negative (blood or urinary) pregnancy test for female patients of reproductive age 5. Willingness to receive contraception during and seven days after the administration of the study drug. 6. Presence of one or more of the following infections: hospital-acquired pneumonia (HAP), health-care associated pneumonia (HCAP), ventilator-associated pneumonia (VAP), primary Gram-negative bacteremia and intra-abdominal infections. Definitions for these infections are given below. 7. Presence of sepsis as defined by the Sepsis-3 classification criteria^3^ 8. Respiratory dysfunction defined as one PaO_2_/FiO_2_ ratio below 200 9. Total SOFA points for organ dysfunctions other than the respiratory function more than 3. |
| **Exclusion criteria** | 1. Denial for informed consent 2. Age inferior to 18 years 3. Pregnancy (confirmed by blood or urinary pregnancy test) or lactation for female patients of reproductive age. 4. Unwillingness to receive contraception during and seven days after the administration of the study drug. 5. HIV infection (with known CD4 cell count ≤ 200/mm^3^) 6. Solid organ, or bone marrow transplantation 7. Corticosteroid oral or intravenous intake greater than 0.4 mg/kg of equivalent prednisone daily over the last 15 days 8. Known active neoplasms compromising short-term survival (1 month) 9. Neutropenia <1000/mm^3^ 10. Known allergy to macrolides 11. Previous participation in the study 12. Administration of a macrolide for the current infection |
| **Study groups** | Blinded 1:1 allocation to one of the following:   - *Placebo*; patients receive water for injection at a volume of 20ml diluted to a final volume of 250 ml dextrose in water 5%. This is infused once daily within 1 hour for four consecutive days. - *Active drug*; patients receive 1g of clarithromycin dissolved into 20 ml water for injection and then diluted to a final volume of 250 ml dextrose in water 5%. This is infused once daily within 1 hour for four consecutive days as previously described.   All patients will also receive standard therapy for sepsis, at the discretion of their attending physicians. |
| **Primary study endpoint** | To assess the impact of intravenously administered clartithromycin as adjunctive treatment to standard antibiotic therapy compared to placebo on all-cause 28-day mortality. |
| **Secondary study endpoints** | To assess the effect of clarithromycin treatment compared to placebo treatment on the following:   - 28-day mortality in the subgroup of patients with septic shock - All-cause 90-day mortality - Early sepsis response, defined by an at least 25% decrease of day 1 SOFA score on day 3 - Sepsis response; this is defined by an at least 25% decrease of day 1 SOFA score on day 7 - New sepsis episode until day 28. A new sepsis episode is noted in any patient who experiences at least 25% decrease of day 1 SOFA score on day 7 and who has further increase of day 7 total SOFA by at least 2 points, consequent to infection - Time until new sepsis episode. A new sepsis episode is noted in any patient who experiences at least 25% decrease of day 1 SOFA score on day 7 and who has further increase of day 7 total SOFA by at least 2 points, consequent to infection - Biomarkers of sepsis-induced immunosuppression through transcriptome, metabolome, microbiome and cell population analysis - Real cost of hospitalization (interventions, medication) until hospital discharge |
| **Sample size calculation** | This is done for the primary study endpoint. We are planning a study of independent cases and controls with 1 control per case. Prior data indicate that 28-day mortality among placebo-treated patients with sepsis and respiratory dysfunction was 55% and that this was decreased to 30% in patients receiving clarithromycin. We need to study 55 subjects into each group to be able to reject the null hypothesis that the failure rates for both groups are equal with probability (power) 0.8. The Type I error probability associated with this test of this null hypothesis is 0.10. We will use a continuity-corrected chi-squared statistic or Fisher’s exact test to evaluate this null hypothesis. |

**INTRODUCTION**

Sepsis is a condition with actually rising incidence, estimated around 19 cases per 1000 hospitalizations per year in academic hospitals in USA^1^ and similar trends in Europe^2^. It is associated with unacceptably high early (in-hospital) mortality of 40-50%^3^. Current guidelines promote best practice by early recognition and management with timely antibiotic administration, fluids, vasopressors and early identification/ control of infection source^4^. However, in spite of adherence to more intensive and costly protocols of early goal-directed therapy, no further decrease in mortality is achieved^5^. Syndromic approaches on sepsis and therapies targeting immune modulation are under evaluation and failures may partly be due to incomplete understanding of underlying pathophysiological mechanisms and immunological phases (pro and anti-inflammatory) of sepsis^6^.

Macrolides, such as clarithromycin or azithromycin have been shown beneficial in reducing COPD exacerbations (OR 0.55; 95% CI 0.39-0.77; p<0.001) partly through anti-inflammatory properties^7^. Besides, when added to a beta-lactam regimen in community-acquired pneumonia, macrolide therapy was associated with reduced mortality risk (OR 0.67; 95% CI 0.61-0.73; p<0.001) in a recent meta-analysis of observational studies including 42942 patients^8^. An immunomodulatory effect of macrolides, beyond their antimicrobial action may explain these findings.

Clarithromycin as adjunctive treatment to standard antibiotic therapy has been used by our group in two previous large-scale multi-center RCTs conducted in Greece after approval from the Greek regulatory authorities. The first RCT studied 200 patients with sepsis due to VAP and it was conducted between 2004 and 2005 ( [www.clinicaltrials.gov](http://www.clinicaltrials.gov) NCT00297674). Identified pathogens mainly included gram-negative bacteria i.e. *Acinetobacter baumanii* and *Pseudomonas aeruginosa* that do not belong to the usual antimicrobial spectrum of macrolides. Although 28-day mortality in both arms was similar, patients assigned to the clarithromycin arm experienced earlier weaning from mechanical ventilation and more rapid resolution of VAP, compared to the placebo arm. In the subgroup of patients with septic shock and multiple organ dysfunction syndrome (MODS), probability of sepsis-related death was significantly lower (OR 3.78 vs 19; *p* = 0.043). Serious adverse events (SAEs) occurred in 3 (3%) clarithromycin-treated patients, with no clear causative link with the study drug in 2 out of 3 cases^9^. Furthermore, hospitalization costs associated with clarithromycin use were significantly reduced (by 7000 euros/ patient) compared to the placebo arm^10^.

The second multi-center RCT compared the efficacy of clarithromycin versus placebo added to standard antibiotic therapy in patients with gram-negative sepsis caused by acute pyelonephritis, intra-abdominal infections and primary gram-negative bacteremia; 600 patients were included between 2007 and 2011 ([www.clincialtrials.gov](http://www.clincialtrials.gov) NCT01223690). Overall mortality at 28 days in both arms did not differ, but probability of death due to septic shock and MODS was shown once more lower in the clarithromycin-treated group (OR 3.58 vs 6.21; p = 0.036). Interestingly, survival benefit from clarithromycin was even greater in those patients suffering from adult respiratory distress syndrome (ARDS). SAEs were described in 2 (0.7%) patients treated with clarithromycin, while its use was associated with saving of a median of 1000 euros/ hospitalized patient^11^. No QT space prolongation or any arrhythmia was observed in both aforementioned trials.

Immunomodulatory effects of clarithromycin in sepsis are not yet elucidated. The analysis of circulating monocytes and of circulating cytokines of patients participating in the first RCT showed that treatment with clarithromycin was associated with a decline in IL-10/ TNFα ratio, greater apoptosis of monocytes, enhanced antigen presentation capacity of monocytes, as well as improved capacity of monocytes for cytokine production suggesting an effect consistent with reversal of sepsis-induced immunosuppression^12^. These findings in conjunction with evidence generated from in vitro and animal experiments suggest modulation of the immune response as the mechanism of action of clarithromycin^13, 14^.

In both RCTs, clarithromycin was administered intravenously at a dose of 1g as continuous one-hour intravenous infusion for three or four days. The drug was safe, well tolerated and cost-effective. However, benefit from treatment in both RCTs was shown only after sub-group analysis of the sub-group of patients with respiratory dysfunction and not when analysis comprised the entire study population. As a consequence, in order to consolidate the benefit of clarithromycin among patients with sepsis and respiratory dysfunction, an RCT is needed in a patient population with sepsis and respiratory dysfunction. This RCT should also comprise a study population with infections likely to be caused by Gram-negative bacteria that do not belong to the antimicrobial spectrum of clarithromycin.

**AIM OF THE STUDY**

The study is aiming to prove the efficacy of intravenous treatment of clarithromycin in the reduction of 28-day mortality among patients with sepsis and respiratory dysfunction. Secondary aims are the effect on overall 90-day mortality, sepsis resolution and recurrence, 28-day mortality in the subgroup of patients with septic shock and, finally, impact on genomic, metabolomic and microbiome profile, especially biomarkers of sepsis-induced immunosuppression.

**STUDY DESIGN**

***Type of study and study sites***

This is a double- blind, randomized, placebo-controlled clinical study that will be conducted in patients admitted in departments of Intensive Care Medicine (ICU) and Internal Medicine in Greece and Belgium. This is a phase IV RCT and the study drug will be provided by the Sponsor. The study protocol will be submitted for approval to the Institutional Review Board and subsequently to the Regulatory Authorities of each country; these are the National Ethics Committee and the National Organization of Medicine of Greece or the Federal Agency of Drugs and Medicinal Products of Belgium. After study approval and before enrolment of the first patient, the study will be registered at the website [www.clinicaltrials.gov](http://www.clinicaltrials.gov).

***Inclusion criteria***

ALL following criteria should be met for the inclusion of a patient in the study:

- Adult patients (≥18 years)
- Patients of both genders
- Informed consent form signed by patient or by first-degree relative in case of patient unable to consent
- Negative (blood or urinary) pregnancy test for female patients of reproductive age
- Willingness to receive contraception during and seven days after the administration of the study drug.
- Presence of one or more of the following infections: hospital-acquired pneumonia (HAP), health-care associated pneumonia (HCAP), ventilator-associated pneumonia (VAP), primary Gram-negative bacteremia and intra-abdominal infections. Definitions for these infections are given below.
- Presence of sepsis as defined by the Sepsis-3 classification criteria^3^ (cfr definitions section below)
- Respiratory dysfunction defined as one PaO_2_/FiO_2_ ratio inferior to 200, independently of the PEEP level.
- Total SOFA points for organ dysfunctions other than the respiratory function more than 3

***Exclusion criteria***

Patients who meet ANY of the exclusion criteria below cannot be enrolled in the study:

- Denial for informed consent
- Age inferior to 18 years
- Pregnancy (confirmed by blood or urinary pregnancy test) or lactation for female patients of reproductive age.
- Unwillingness to receive contraception during and seven days after the administration of the study drug.
- HIV infection (with known CD4 cell count ≤ 200/mm^3^)
- Solid organ, or bone marrow transplantation
- Corticosteroid oral or intravenous intake greater than 0.4 mg/kg of equivalent prednisone daily over the last 15 days
- Known active neoplasms compromising short-term survival (1 month)
- Neutropenia <1000/mm^3^
- Known allergy to macrolides
- Previous participation in the study
- Administration of a macrolide for the current infection

***Definitions***

Sepsis is defined by the following criteria, based on sepsis-3 classification^3^:

- Total SOFA score of 2 or more points for patients who are admitted with infection at the emergency department (see APPENDIX I) or
- Increase of admission SOFA score by 2 or more points consequent to infection, for patients already hospitalized (see APPENDIX I)

Hospital-acquired pneumonia (HAP) is defined by the presence of a new or progressive radiographic lung infiltrate in a non-intubated patient hospitalized for more than 48 hours who presents with at least two of the following clinical features:

- Core temperature equal or greater than 38°C
- Total white blood cell count more than 12,000/mm^3^
- Rales or bronchial breath sounds on physical examination
- Purulent sputum
- More than 20 breaths/minute
- Serum procalcitonin more than 0.25 ng/ml
- Gram stain of tracheobronchial secretions or bronchoalveolar lavage fluid indicating the predominance of Gram-negative bacilli

Health-care associated pneumonia (HCAP) is defined by the presence of a new or progressive radiographic lung infiltrate in a non-intubated patient who has at least one of the following risk factors for HCAP^15^:

- Hospitalization the last 90 days
- Residency in a long-term care facility
- Under regular hemodialysis

AND who presents with at least two of the following clinical features:

- Core temperature equal or greater than 38°C
- Total white blood cell count more than 12,000/mm^3^
- Rales or bronchial breath sounds on physical examination
- Purulent sputum
- More than 20 breaths/minute
- Serum procalcitonin more than 0.25 ng/ml
- Gram stain of tracheobronchial secretions or bronchoalveolar lavage fluid indicating the predominance of Gram-negative bacilli^15^

Ventilator-associated pneumonia (VAP) is defined by the presence of a new or progressive radiographic lung infiltrate in a patient who is under mechanical ventilation for at least 48 hours AND who presents with at least two of the following clinical features:

- Core temperature equal or greater than 38°C
- Total white blood cell count more than 12,000/mm^3^
- Purulent tracheobronchial secretions
- Serum procalcitonin more than 0.25 ng/ml
- Gram stain of tracheobronchial secretions or bronchoalveolar lavage fluid indicating the predominance of Gram-negative bacilli^15^

Primary Gram-negative bacteremia (BSI) is defined as the isolation of at least one Gram-negative microorganism from a blood culture of a peripheral vein of a patient that is not related to infection of a central line and who presents with ALL the following features^16^:

- Core temperature equal or greater than 38°C or total white blood cell count more than 12,000/mm^3^
- Thorough clinical and radiological investigation has failed to identify the primary infection site

Intra-abdominal infection (IAI) is defined as the presence of ALL the following features^16^:

- Core temperature equal or greater than 38°C or total white blood cell count more than 12,000/mm^3^
- Radiological findings from abdominal ultrasound or abdominal computed tomography consistent with one IAI.

***Study drug preparation and administration***

A separate allocation sequence will be generated for each study site, following a 1:1 design. An allocation sequence will be generated as sealed envelope from a statistician with 1:1 randomization per study site. The envelope is unsealed by an unblinded investigator (or the pharmacist), who is preparing the study drug (placebo or active drug). The preparations will be visually similar and allow blinded administration. Clarithromycin is provided in a form of vial with 500mg of amorphous powder. Two vials are dissolved in 10ml of water for injection each. The prepared 20ml solution is then further diluted to a final volume of 250 ml dextrose in water 5% that is directly connected to the infusion device that leading to a catheter already inserted in a central or peripheral vein. Placebo will consist in 20 ml of water for injection, diluted to a final volume of 250 ml dextrose in water 5% that is directly connected to the infusion device leading to a catheter already inserted in a central or peripheral vein. According to the generated allocation sequence, patients of each study site can be randomly assigned to one of the following two groups:

- *Placebo*; patients receive water for injection at a volume of 20ml diluted to a final volume of 250 ml dextrose in water 5%. This is infused once daily within 1 hour for four consecutive days. All patients allocated to the placebo group will also receive standard therapy at the discretion of their attending physicians.
- *Active drug*; patients receive 1g of clarithromycin dissolved into 20 ml water for injection and then diluted to a final volume of 250 ml dextrose in water 5%. This is infused once daily within 1 hour for four consecutive days as previously described^9, 11^. All patients allocated to the active drug group will also receive standard therapy at the discretion of their attending physicians.

***Study visits***

*Screening visit*

When a patient meets ALL inclusion criteria and NONE of the exclusion criteria, he/she can be enrolled in the study. The criteria are judged based on data available from the patient’s file. Female patients of reproductive age should be screened with a urinary pregnancy test. QT prolongation or prior arrhythmia is not considered as an exclusion criterion for the study, ^9,11^ but may be considered in the decision making of the principal investigator. The same patient cannot be enrolled twice in this study.

*Patient follow-up*

Follow-up of every patient will be done daily until day 28 or hospital discharge (whatever comes first).

Visit 1 is on day 1. Procedures of this day include:

- Recording of demographics, medical history, co-morbidities (Charlson’s Comorbidity Index), ^17^ SOFA score (see APPENDIX I); Acute Physiology and Chronic Health Evaluation (APACHE) II score (see APPENDIX II), available blood cell count, biochemistry, coagulation time, urine output, blood gas analysis, recording of suspected infection site, available radiological findings, relevant for the current infection microbiology and antimicrobial susceptibility testing if available, administered antimicrobials, other administered drugs and need for source infection control either by percutaneous interventions or by any operation.
- Sampling of 25 ml of venous blood; 3ml is collected into one PAXgene tube or a tube with RNA*later*®; 7ml is collected into one pyrogen-free tube; and 15ml is collected into one EDTA-coated tube
- Collection of stool culture or rectal swab
- Administration of the study drug
- Evaluation of potential adverse events

Visit 2 is on day 2. Procedures of this day include:

- Recording of SOFA score (see APPENDIX I); available blood cell count, biochemistry, coagulation times, urine output, blood gas analysis, available radiological findings, relevant for the current infection microbiology and antimicrobial susceptibility testing if available, administered antimicrobials, other administered drugs and need for source infection control either by percutaneous interventions or by any operation.
- Survival status
- Administration of the study drug
- Evaluation of potential adverse events

Visit 3 is on day 3. Procedures of this day include:

- Recording of SOFA score (see APPENDIX I); available blood cell count, biochemistry, coagulation times, urine output, blood gas analysis, available radiological findings, relevant for the current infection microbiology and antimicrobial susceptibility testing if available, administered antimicrobials, other administered drugs and need for source infection control either by percutaneous interventions or by any operation.
- Evaluation of early sepsis response, defined by at least 25% decrease of visit 1 SOFA on visit 3
- Survival status
- Administration of the study drug
- Evaluation of potential adverse events

Visit 4 is on day 4. Procedures of this day include:

- Recording of SOFA score (see APPENDIX I); available blood cell count, biochemistry, coagulation times, urine output, blood gas analysis, recording of suspected or proven infection site, available radiological findings, relevant for the current infection microbiology and antimicrobial susceptibility testing if available, administered antimicrobials, other administered drugs and need for source infection control either by percutaneous interventions or by any operation.
- Survival status
- Administration of the study drug
- Evaluation of potential adverse events

Visit 5 is on day 5. Procedures of this day include:

- Recording of SOFA score (see APPENDIX I); available blood cell count, biochemistry, coagulation times, urine output, blood gas analysis, available radiological findings, relevant microbiology and antimicrobial susceptibility testing if available, administered antimicrobials, other administered drugs and need for source infection control either by percutaneous interventions or by any operation. Given the previous data from phase IV trials, serial ECGs are not required for this protocol.
- Survival status
- Sampling of 25 ml of venous blood; 3ml is collected into one PAXgene tube or a tube with RNA*later*®; 7ml is collected into one pyrogen-free tube; and 15ml is collected into one EDTA-coated tube
- Collection of stool or rectal swab.
- Evaluation of resolution of the infection and potential recurrence of infection according to the judgment of the attending physician
- Evaluation of potential adverse events

Visit 6 is on day 6. Procedures of this day include:

- Recording of SOFA score (see APPENDIX I); available blood cell count, biochemistry, coagulation times, urine output, blood gas analysis, available radiological findings, clinically relevant microbiology and antimicrobial susceptibility testing, administered antimicrobials, other administered drugs and need for source infection control either by percutaneous interventions or by any operation.
- Survival status
- Evaluation of resolution of the infection and potential recurrence of infection according to the judgment of the attending physician
- Evaluation of potential adverse events

Visit 7 is on day 7. Procedures of this day include:

- Recording of SOFA score (see APPENDIX I); available blood cell count, biochemistry, coagulation times, urine output, blood gas analysis, available radiological findings, clinically relevant microbiology and antimicrobial susceptibility testing, administered antimicrobials, other administered drugs and need for source infection control either by percutaneous interventions or by any operation.
- Survival status
- Evaluation of sepsis response, defined by at least 25% decrease of SOFA score of visit 1
- Evaluation of resolution of the infection and potential recurrence of infection according to the judgment of the attending physician
- Evaluation of potential adverse events

Visit 8 is on day 8. Procedures of this day include:

- Recording of SOFA score (see APPENDIX I); available blood cell count, biochemistry, coagulation times, urine output, blood gas analysis, available radiological findings, clinically relevant microbiology and antimicrobial susceptibility testing, administered antimicrobials, other administered drugs and need for source infection control either by percutaneous interventions or by any operation.
- Survival status
- Evaluation of resolution of the infection and potential recurrence of infection according to the judgment of the attending physician
- Evaluation of potential adverse events

Visit 9 is on day 9. Procedures of this day include:

- Recording of SOFA score (see APPENDIX I); available blood cell count, biochemistry, coagulation times, urine output, blood gas analysis, available radiological findings, clinically relevant microbiology and antimicrobial susceptibility testing, administered antimicrobials, other administered drugs and need for source infection control either by percutaneous interventions or by any operation.
- Survival status
- Evaluation of resolution of the infection and potential recurrence of infection according to the judgment of the attending physician
- Evaluation of potential adverse events

Visit 10 is on day 10. Procedures of this day include:

- Recording of SOFA score (see APPENDIX I); available blood cell count, biochemistry, coagulation times, urine output, blood gases, available radiological findings, clinically relevant microbiology and antimicrobial susceptibility testing, administered antimicrobials, other administered drugs and need for source infection control either by percutaneous interventions or by any operation.
- Survival status
- Evaluation of resolution of the infection and potential recurrence of infection according to the attending physician
- Sampling of 20 ml of venous blood; 5ml is collected into one pyrogen-free tube; and 15ml is collected into one EDTA-coated tube
- Evaluation of potential adverse events

Visits 11-28 are on days 11-28 respectively. They include

- Recording of SOFA score if available
- Administered antimicrobials, other administered drugs and need for source infection control either by percutaneous interventions or by any operation.
- Survival status
- Evaluation of resolution of the infection and potential infection recurrence according to the judgment of the attending physician
- Evaluation of potential adverse events

Last visit: A phone call follow-up will be done on day 90 to assess survival status.

All information will be recorded on a specific paper Case Report Form (CRF).

The procedure to follow at each study visit is shown in APPENDIX III.

**Laboratory analysis**

Collected samples will be transported to the central lab that is the Laboratory of Immunology and Infectious Diseases of the 4^th^ Department of Internal Medicine, ATTIKON University General Hospital, Athens, Greece. Analysis will be done as follows:

- PAXgene tubes or tubes with RNA*later*® for full transcriptomics
- EDTA tubes for isolation of peripheral blood mononuclear cells and further cytokine stimulation, as well as flow cytometry.
- Serum for metobolomic analysis and serum markers

**STUDY ENDPOINTS**

**Primary study endpoint**

To assess the impact of intravenously administered clartithromycin as adjunctive treatment to standard antibiotic therapy compared to placebo on all-cause 28-day mortality.

**Secondary study endpoints**

To assess the effect of clarithromycin treatment compared to placebo treatment on the following:

- 28-day mortality in the subgroup of patients with septic shock
- All-cause 90-day mortality
- Early sepsis response, defined by an at least 25% decrease of day 1 SOFA score on day 3
- Sepsis resolution; this is defined by an at least 25% decrease of day 1 SOFA score on day 7
- New sepsis episode until day 28. A new sepsis episode is defined as a further increase of day 7 SOFA score by at least 2 points consequent to infection, in a patient who has experienced previous sepsis resolution (at least 25% decrease of day 1 SOFA score on day 7)
- Time until new sepsis episode. A new sepsis episode is noted in any patient who experiences any more than 25% decrease of day 1 SOFA score on day 7 and who has further increase of day 7 total SOFA by at least 2 points, consequent to infection
- Biomarkers of sepsis-induced immunosuppression, through metabolome, transcriptome, microbiome and cell population analysis
- Real cost at hospital discharge

**POWER STUDY CALCULATION**

This is done for the primary study endpoint. We are planning a study of independent cases and controls with 1 control per case. Prior data indicate that the mortality among placebo-treated patients with sepsis and respiratory dysfunction was 55% and that this was decreased to 30%^11^. We need to study 55 subjects into each group to be able to reject the null hypothesis that the failure rates for both groups are equal with probability (power) 0.8. The Type I error probability associated with this test of this null hypothesis is 0.10. We will use a continuity-corrected chi-squared statistic or Fisher’s exact test to evaluate this null hypothesis.

**STATISTICAL ANALYSIS**

Qualitative endpoints will be analyzed by the Fisher’s exact test or continuity corrected Chi- square test, as appropriate. Logistic regression models will be used to evaluate variables associated with 28-day mortality. The effect on sepsis-induced immunosuppression will be a composite endpoint and it will result from the bioanalysis of gene expression, circulating cytokines and stimulated cytokines of patients. More precisely, bioanalysis of gene expression is anticipated to provide pathways modulated by treatment. These pathways will be validated by measurement of representative cytokines in cell supernatants. Comparisons of over-time changes will be done between the two groups of treatment.

For the cost analysis, cost of each intervention (drugs, hotel stay, radiology, laboratory tests) will be counted and compared between groups, with non-parametric Mann-Whitney test, as previously described^10^. A p - value lower than 0.05 will be considered statistically significant, unless stated otherwise.

**DURATION OF THE STUDY**

The duration of the study is estimated to two years after approval by the Greek and Belgian regulatory authorities.

**ADVERSE EVENTS**

Adverse events (AEs) and Serious Adverse Events (SAEs) will be collected from baseline until the last patient’s last evaluation. An adverse event is any undesirable medical occurrence in a subject receiving a pharmaceutical product and does not necessarily have a causal link with this treatment. The time relationship is established if the AE occurs during therapeutic treatment and until 5 half-lives after treatment discontinuation. The adverse event may be a sign, a symptom, or an abnormal laboratory finding.

*Serious adverse events* must be reported to the Greek and Belgian agencies of pharmacovigilance for marketed products (EOF and AFMPS- within 7 days for life-threatening situations and within 15 days for serious adverse events of occurrence. The Principal Investigators are held to report SAEs to the sponsor within 24h after having the information.

If an adverse event meets any of the following criteria, it is considered as a Serious Adverse Event (SAE):

- Life-threatening situation The subject was at risk of death at the time of the adverse event. It does not refer to the hypothetical risk of death if the AE was more severe or had come to progress.
- Hospitalization
- Persistent or significant disability/ incapacity Any AE having an outcome that is associated with a substantial disruption of the ability to carry out normal life functions, including the ability to work. This is not intended to include transient interruption of daily activities.
- Important medical events that may not result in death, be life-threatening, or require hospitalization, may be considered as SAE when, based upon appropriate medical judgment, they may jeopardize the subject and may require medical or surgical intervention to prevent one of the outcomes listed above, i.e. death, a life-threatening adverse event/experience, inpatient hospitalization or prolongation of existing hospitalization, a persistent or significant disability/incapacity, (or a congenital abnormality/birth defect). Examples of such medical events include allergic bronchospasm requiring intensive treatment in an emergency room or at home, blood dyscrasias or convulsions that do not result in inpatient hospitalization, or the development of drug dependency or drug abuse.
- Pregnancy
- Spontaneous and elective abortions experienced by study subject

Since death is a study endpoint, deaths will not be reported as SAEs.

*A non-serious adverse event* is any untoward medical occurrence in a subject receiving a pharmaceutical product, and that does not necessarily have a causal link with this treatment. A non-serious adverse event is one that does not meet the previous definition of a SAE and must be reported to the EOF and AFMPS according to local laws for marketed products. The severity of the non-serious adverse events will be graded using the following definitions:

- *Mild* - the adverse event is transient and well tolerated by the patient
- *Moderate* – The adverse events causes discomfort and affects the usual activities of the patient.
- *Severe* – The adverse events affects the usual activities of the patient to an important degree and may cause disability or be life-threatening.

*Relationship with the drug*

The investigator will use the following definitions to assess the relationship of the adverse events with the study drug:

- Probably Related: The adverse event has a strong time relationship with the drug or relapses if re-induced, and another aetiology is improbable or clearly less probable.
- Possibly Related: The adverse event has a strong time relationship to the drug and an alternative aetiology is as probable or less probable.
- Probably not Related: The adverse event has a slight or no time relationship to the drug and/or there is a more probable alternative aetiology.
- Unrelated: The adverse event is due to an underlying or concomitant disease or to another pharmaceutical product and is not related to the drug (no time relationship and a much more probable alternative aetiology).

If an investigator’s opinion of possibly related, probably not related or not related to study drug is given, an alternate etiology must be provided by the investigator. Please note that a severe adverse event/experience is not necessarily serious, as the term severe is a measure of intensity while a serious adverse event is determined based on the aforementioned regulatory criteria.

**REFERENCES**

1. Kadri SS, Rhee C, Strich JR, Morales MK, Hohmann S, Menchaca J, et al. Estimating ten-year trends in septic shock incidence and mortality in United States academic medical centers using clinical data*.* *Chest* 2016
2. SepNet Critical Care Trials G. Incidence of severe sepsis and septic shock in German intensive care units: the prospective, multicentre INSEP study*.* *Intensive Care Med* 2016; 42: 1980-9.
3. Singer M, Deutschman CS, Seymour CW, Shankar-Hari M, Annane D, Bauer M, et al. The third international consensus definitions for sepsis and septic shock (Sepsis-3)*.* *JAMA* 2016; 315: 801-10.
4. Dellinger RP, Levy MM, Rhodes A, Annane D, Gerlach H, Opal SM, et al. Surviving Sepsis Campaign Guidelines Committee including The Pediatric S. Surviving Sepsis Campaign: international guidelines for management of severe sepsis and septic shock, 2012*.* *Intensive Care Med* 2013;39:165-228.
5. Mouncey PR, Osborn TM, Power GS, Harrison DA, Sadique MZ, Grieve RD, et al. Trial of early, goal-directed resuscitation for septic shock*.* *N Engl J Med* 2015; 372: 1301-11.
6. Ranieri VM, Thompson BT, Barie PS, Dhainaut JF, Douglas IS, Finfer S, et al. Drotrecogin alfa (activated) in adults with septic shock*.* *N Engl J Med* 2012; 366: 2055-64.
7. Herath SC, Poole P. Prophylactic antibiotic therapy for chronic obstructive pulmonary disease (COPD)*.* *Cochrane Database Syst Rev* 2013: CD009764.
8. Nie W, Li B, Xiu Q. Beta-lactam/macrolide dual therapy versus beta-lactam monotherapy for the treatment of community-acquired pneumonia in adults: a systematic review and meta-analysis*.* *J Antimicrob Chemother* 2014; 69: 1441-6.
9. Giamarellos-Bourboulis EJ, Pechère JC, Routsi C, Plachouras D, Kollias S, Raftogiannis M, et al. Effect of clarithromycin in patients with sepsis and ventilator-associated pneumonia*.* *Clin Infect Dis* 2008; 46: 1157-64.
10. Tsaganos T, Raftogiannis M, Pratikaki M, Christodoulou S, Kotanidou A, Papadomichelakis E, et al. Clarithromycin leads to long-term survival and cost benefit in ventilator-associated pneumonia and sepsis*.* *Antimicrob Agents Chemother* 2016; 60: 3640-6.
11. Giamarellos-Bourboulis EJ, Mylona V, Antonopoulou A, Tsangaris I, Koutelidakis I, Marioli A, et al. Effect of clarithromycin in patients with suspected Gram-negative sepsis: results of a randomized controlled trial*.* *J Antimicrob Chemother* 2014; 69: 1111-8.
12. Spyridaki A, Raftogiannis M, Antonopoulou A, Tsaganos T, Routsi C, Baziaka F, et al. Effect of clarithromycin in inflammatory markers of patients with ventilator-associated pneumonia and sepsis caused by Gram-negative bacteria: results from a randomized clinical study*.* *Antimicrob Agents Chemother* 2012; 56: 3819-25.
13. Schultz MJ, Speelman P, Hack CE, Buurman WA, van Deventer SJ, van der Poll T. Intravenous infusion of erythromycin inhibits CXC chemokine production, but augments neutrophil degranulation in whole blood stimulated with Streptococcus pneumoniae*.* *J Antimicrob Chemother* 2000; 46: 235-40.
14. Kanoh S, Rubin BK. Mechanisms of action and clinical application of macrolides as immunomodulatory medications*.* *Clin Microbiol Rev* 2010; 23: 590-615.
15. Kalil AC, Metersky ML, Klompas M, Muscedere J, Sweeney DA, Palmer LB, et al. Management of adults with hospital-acquired and ventilator-associated pneumonia: 2016 clinical practice guidelines by the Infectious Diseases Society of America and the American Thoracic Society*.* *Clin Infect Dis* 2016; 63: e61-e111.
16. Calandra T, Cohen J. The international sepsis forum consensus conference on definitions of infection in the intensive care unit*.* *Crit Care Med* 2005; 33: 1538-48.
17. Charslon ME, Pompei P, Ales KL, MacKenzie CR. A new method of classifying prognostic comorbidity in longitudinal studies: development and validation. *J Chronic Dis* 1987; 40: 373-383

**APPENDIX I** The SOFA score^3^

| **Variable** | **0 points** | **1 point** | **2 points** | **3 points** | **4 points** |
| --- | --- | --- | --- | --- | --- |
| PaO_2_/FiO_2_(mmHg) | ≥400 | <400 | <300 | <200 | <100 |
| Platelets (per mm^3^) | ≥150 | <150 | <100 | <50 | <20 |
| Hypotension | MAP≥ 70 mmHg | MAP<70 mmHg | Dobutamine whatever dose | Adrenaline ≤0.1* or  Noradrenaline≤ 0.1* | Adrenaline>0.1* or  Noradrenaline >0.1* |
| Glasgow Coma Scale | 15 | 13-14 | 10-12 | 6-9 | <6 |
| Bilirubin (mg/dl) | <1.2 | 1.2-1.9 | 2.0-5.9 | 6.0-11.9 | ≥12 |
| Creatinine (mg/dl) or Urine output | <1.2 | 1.2-1.9 | 2.0-3.4 | 35-4.9 or <500ml/day | ≥5.0 or  <200ml/day |

*μg/kg/min

Each variable is scored between 0 and 4. The SOFA score is the sum of the score of each variable

**APPENDIX II Calculation of the APACHE (acute physiology and chronic health evaluation) II score.**

| **Physiological Variable** | **4 points** | **3 points** | **2 points** | **1 point** | **0** | **1 point** | **2 points** | **3 points** | **4 points** |
| --- | --- | --- | --- | --- | --- | --- | --- | --- | --- |
| Temperature | >41 | 39-40,9 |  | 38,5-38,9 | 36-38,4 | 34-35,9 | 32-33,9 | 30-31,9 | ≤29,9 |
| Mean arterial pressure (mmHg) | ≥160 | 130-159 | 110-129 |  | 70-109 |  | 50-69 |  | ≤49 |
| Heart rate | ≥180 | 140-179 | 110-139 |  | 70-109 |  | 55-69 | 40-54 | ≤39 |
| Respiratory rate | ≥50 | 35-49 |  | 25-34 | 12-24 | 0-12 | 6-9 |  | ≤5 |
| Oxygenation   1. FiO_2_≥0,5 record A-aDO_2_ 2. FiO2<0,5 record PO_2_ | ≥500 | 350-349 | 200-349 |  | <200  >70 | 61-70 |  | 55-60 | <55 |
| Arterial pH | ≥7,7 | 7,6-7,69 |  | 7,5-7,59 | 7,33-7,49 |  | 7,26-7,32 | 7,15-7,24 | ≤7,14 |
| Serum HCO_3_ (mEq/l) | ≥52 | 41-51,9 |  | 32-40,9 | 22-31,9 |  | 18-21,9 | 15-17,9 | <15 |
| Serum sodium (mEq/l) | ≥180 | 160-179 | 155-159 | 150-154 | 130-149 |  | 120-129 | 111-119 | ≤110 |
| Serum potassium (mEq/l) | ≥7 | 6-6,9 |  | 5,5-5,9 | 3,5-5,4 | 3-3,4 | 2,5-2,9 |  | <2,5 |
| Serum creatinine (mg/dl) | ≥3,5 | 2-3,4 | 1,5-1,9 |  | 0,6-1,4 |  | <0,6 |  |  |
| Haematocrit (%) | ≥60 |  | 50-59,9 | 46-49,9 | 30-45,9 |  | 20-29,9 |  | <20 |
| White blood cell count (total/mm^3^) | ≥40 |  | 20-39,9 | 15-19,9 | 3-14,9 |  | 1-2,9 |  | <1 |
| Glascow coma scale (GCS) | Score 15- current GCS | | | | | | | | |
| Add age points as follow:  Add chronic health points | ≤44years 0 points, 45-54 years 2 points, 55-64 years 3 points, 65-74 years 5 points, ≥75 years 6 points | | | | | | | | |

**APPENDIX III** Procedures on each study visit (until day 28 or until hospital discharge)

|  | **Study visits** | | | | | | | | | | | | | | | | | | | | | | | | | | | | |  |
| --- | --- | --- | --- | --- | --- | --- | --- | --- | --- | --- | --- | --- | --- | --- | --- | --- | --- | --- | --- | --- | --- | --- | --- | --- | --- | --- | --- | --- | --- | --- |
|  | **1** | **2** | **3** | **4** | **5** | **6** | **7** | **8** | **9** | **10** | **11** | **12** | **13** | **14** | **15** | **16** | **17** | **18** | **19** | **20** | **21** | **22** | **23** | **24** | **25** | **26** | **27** | **28** | **Last** | |
| Study drug | x | x | x | x |  |  |  |  |  |  |  |  |  |  |  |  |  |  |  |  |  |  |  |  |  |  |  |  |  | |
| SOFA score* | x | x | x | x | x | x | x | x | x | x | x | x | x | x | x | x | x | x | x | x | x | x | x | x | x | x | x | x |  | |
| Survival |  | x | x | x | x | x | x | x | x | x | x | x | x | x | x | x | x | x | x | x | x | x | x | x | x | x | x | x | x | |
| CCI | x |  |  |  |  |  |  |  |  |  |  |  |  |  |  |  |  |  |  |  |  |  |  |  |  |  |  |  |  | |
| APACHE score | x |  |  |  |  |  |  |  |  |  |  |  |  |  |  |  |  |  |  |  |  |  |  |  |  |  |  |  |  | |
| Vital signs** | x | x | x | x | x | x | x | x | x | x | x | x | x | x | x | x | x | x | x | x | x | x | x | x | x | x | x | x |  | |
| Concomitant drugs/ procedures | x | x | x | x | x | x | x | x | x | x | x | x | x | x | x | x | x | x | x | x | x | x | x | x | x | x | x | x |  | |
| Infectious site identification | x |  |  | x |  |  |  |  |  |  |  |  |  |  |  |  |  |  |  |  |  |  |  |  |  |  |  |  |  | |
| Resolution/ recurrence of infection** |  |  |  |  |  |  | x | x | x | x | x | x | x | x | x | x | x | x | x | x | x | x | x | x | x | x | x | x |  | |
| AE monitoring | x | x | x | x | x | x | x | x | x | x |  |  |  |  |  |  |  |  |  |  |  |  |  |  |  |  |  |  |  | |
| Microbiology** | x | x | x | x | x | x | x | x | x | x | x | x | x | x | x | x | x | x | x | x | x | x | x | x | x | x | x | x |  | |
| Pregnancy test*** | x |  |  |  |  |  |  |  |  |  |  |  |  |  |  |  |  |  |  |  |  |  |  |  |  |  |  |  |  | |
| PAX Gene | x |  |  |  | x |  |  |  |  |  |  |  |  |  |  |  |  |  |  |  |  |  |  |  |  |  |  |  |  | |
| Serum metabolomics | x |  |  |  | x |  |  |  |  | x |  |  |  |  |  |  |  |  |  |  |  |  |  |  |  |  |  |  |  | |
| PBMCs isolation | x |  |  |  | x |  |  |  |  | x |  |  |  |  |  |  |  |  |  |  |  |  |  |  |  |  |  |  |  | |
| Stool sample | x |  |  |  | x |  |  |  |  |  |  |  |  |  |  |  |  |  |  |  |  |  |  |  |  |  |  |  |  | |

- * SOFA score: if available according to usual clinical practice. On days where SOFA components are not available (e.g. no blood analysis programed by the physician or discharged patient), the last known value will be recorded.
- ** If available and justified by clinical context ***For female patients of reproductive age

# 2. Protocol version 2 (Final version)

**A DOUBLE-BLIND, RANDOMIZED, PLACEBO-CONTROLLED CLINICAL STUDY OF THE EFFICACY OF INTRAVENOUS CLARITHROMYCIN AS ADJUNCTIVE TREATMENT IN PATIENTS WITH SEPSIS AND RESPIRATORY AND MULTIPLE ORGAN DYSFUNCTION SYNDROME**

**Running title: INtravenous CLArithromycin in Sepsis and Multiple Organ Dysfunction Syndrome (INCLASS study)**

**CLINICAL STUDY PROTOCOL**

**Authors:**

**Eleni Karakike, MD and Evangelos J. Giamarellos-Bourboulis, MD, PhD**

**Sponsor and CRO:**

**HELLENIC INSTITUTE FOR THE STUDY OF SEPSIS**

**88, Michalakopoulou str., 11528 Athens, GREECE**

**Protocol version:** 2

**Protocol date:** 05 November 2018

**EudraCT number:** 2017-001056-55

**ATHENS 2018**

**TABLE OF CONTENTS**

|  | Page |
| --- | --- |
| **Disclosure of Principal Investigator** | **3** |
| **Abbreviations** | **4** |
| **Study Synopsis** | **5** |
| **Introduction** | **8** |
| **Aim of the study** | **10** |
| **Study design** | **10** |
| **Type of study and study sites** | **10** |
| **Inclusion criteria** | **11** |
| **Exclusion criteria** | **12** |
| **Definitions** | **12** |
| **Study drug preparation and administration** | **14** |
| **Study visits** | **15** |
| **Laboratory analysis** | **20** |
| **Cost analysis** | **21** |
| **Cost-Effectiveness analysis** | **22** |
| **Study endpoints** | **22** |
| **Study power calculation** | **23** |
| **Statistical analysis** | **23** |
| **Duration of the study** | **24** |
| **Adverse events** | **24** |
| **Quality control and assurance** | **27** |
| **Ethical considerations** | **27** |
| **Protocol adherence and amendments** | **28** |
| **References** | **29** |
| **Appendix I** | **31** |
| **Appendix II** | **32** |
| **Appendix III** | **33** |
| **Appendix IV** | **34** |
| **Appendix V** | **36** |

**DISCLOSURE OF PRINCIPAL INVESTIGATOR**

**Protocol Study Title:** A DOUBLE-BLIND, RANDOMIZED, PLACEBO-CONTROLLED CLINICAL STUDY OF THE EFFICACY OF INTRAVENOUS CLARITHROMYCIN AS ADJUNCTIVE TREATMENT IN PATIENTS WITH SEPSIS AND RESPIRATORY AND MULTIPLE ORGAN DYSFUNCTION SYNDROME.

The herein protocol became known to myself by the Study Sponsor. I understand that the protocol remains as yet unpublished; I certify that all disclosed information to myself for this protocol will remain strictly confidential.

The Principal Investigator,

Print Name

Signature Date

**ABBREVIATIONS**

(S)AE: (serious) adverse event

APACHE: acute physiology and chronic health evaluation

ARDS: acute respiratory distress syndrome

BSI: primary Gram-negative bacteremia

CCI: Charlson comorbidity index

CD4: T4 lymphocytes

COPD: chronic obstructive pulmonary disease

CRF: case report form

DRGs: diagnosis related groups

EDTA: ethylenediamine actic acid

EGFR: epithelial growth factor receptor

EIA: enzyme immunoassay

G: grammar

HAP: hospital-acquired pneumonia

HCAP: health care-associated pneumonia

HIV: human immunodeficiency virus

HPLC: high-performance liquid chromatography

IAI: intra-abdominal infection

ICER: incremental cost-effectiveness ratio

ICF: informed consent form

ICU: intensive care unit

IL: interleukin

MODS: multiple organ dysfunction syndrome

OR: odds ratio

PaO_2_/ FiO_2_: partial arterial oxygen pressure per fraction of inspired oxygen

PBMCs: peripheral blood mononuclear cells

QALY: quality-adjusted life-year

RCT: randomized clinical trial

SOFA: sequential organ failure assessment

SUSAR: suspected, unexpected serious adverse reaction

TNFα: tumor necrosis factor-alpha

VAP: ventilator-associated pneumonia

VEGF: Vascular endothelial growth factor **STUDY SYNOPSIS**

| **Background** | High mortality associated with sepsis and MODS calls for alternative, individualized therapies in selected patients that might benefit form specific interventions. Role of macrolides as potential immunomodulatory treatment in sepsis is promising, but unclear. Subgroup analysis of previous large-scale clinical trials on patients with ventilator-associated pneumonia or gram-negative sepsis, showed that addition of clarithromycin to standard antibiotic therapy conferred a significant survival benefit in the subgroup of patients with respiratory dysfunction and MODS, but this effect has never been investigated through randomized controlled trials in an entire population suffering from these entities. |
| --- | --- |
| **Aim** | The study is aiming to assess the efficacy of intravenous treatment of clarithromycin in the reduction of 28-day mortality among patients with sepsis and respiratory dysfunction. Secondary aims are the effect on 90-day mortality, sepsis resolution, and recurrence, mortality in the subgroup of patients with septic shock and finally, impact on biomarkers of sepsis-induced immunosuppression |
| **Design** | Multicenter, interventional, double blind, randomized, placebo-controlled phase IV study |
| **Inclusion criteria** | 1. Adult patients (≥18 years) 2. Patients of both genders 3. Informed consent form signed by patient or by first-degree relative in case of patient unable to consent 4. Negative (blood or urinary) pregnancy test for female patients of reproductive age 5. Willingness to receive contraception during and seven days after the administration of the study drug. 6. Presence of one or more of the following infections: hospital-acquired pneumonia (HAP), health-care associated pneumonia (HCAP), ventilator-associated pneumonia (VAP), primary Gram-negative bacteremia and intra-abdominal infections. Definitions for these infections are given below. 7. Presence of sepsis as defined by the Sepsis-3 classification criteria^3^ 8. Respiratory dysfunction defined as one PaO_2_/FiO_2_ ratio below 200 9. Total SOFA points for organ dysfunctions other than the respiratory function more than 3. |
| **Exclusion criteria** | 1. Denial for informed consent 2. Age inferior to 18 years 3. Pregnancy (confirmed by blood or urinary pregnancy test) or lactation for female patients of reproductive age. 4. Unwillingness to receive contraception during and seven days after the administration of the study drug. 5. HIV infection (with known CD4 cell count ≤ 200/mm^3^) 6. Solid organ, or bone marrow transplantation 7. Corticosteroid oral or intravenous intake greater than 0.4 mg/kg of equivalent prednisone daily over the last 15 days, or other immunosuppressive therapy 8. Known active neoplasms or other conditions unrelated to sepsis, that are compromising short-term survival (1 month) 9. Neutropenia <1000/mm^3^ 10. Known allergy to macrolides 11. Previous participation in the study 12. Administration of a macrolide for the current infection |
| **Study groups** | Blinded 1:1 allocation to one of the following:   - *Placebo*; patients receive water for injection at a volume of 20ml diluted to a final volume of 250 ml dextrose in water 5%. This is infused once daily within 1 hour for four consecutive days. - *Active drug*; patients receive 1g of clarithromycin dissolved into 20 ml water for injection and then diluted to a final volume of 250 ml dextrose in water 5%. This is infused once daily within 1 hour for four consecutive days as previously described.   All patients will also receive standard therapy for sepsis, at the discretion of their attending physicians. |
| **Primary study endpoint** | To assess the impact of intravenously administered clarithromycin as adjunctive treatment to standard antibiotic therapy compared to placebo on all-cause 28-day mortality. |
| **Secondary study endpoints** | To assess the effect of clarithromycin treatment compared to placebo treatment on the following:   - 28-day mortality in the subgroup of patients with septic shock - All-cause 90-day mortality - Early sepsis response, defined by an at least 25% decrease of day 1 SOFA score on day 3 - Sepsis response; this is defined by an at least 25% decrease of day 1 SOFA score on day 7 - New sepsis episode until day 28. A new sepsis episode is noted in any patient who experiences at least 25% decrease of day 1 SOFA score on day 7 and who has further increase of day 7 total SOFA by at least 2 points, consequent to infection - Time until new sepsis episode. A new sepsis episode is noted in any patient who experiences at least 25% decrease of day 1 SOFA score on day 7 and who has further increase of day 7 total SOFA by at least 2 points, consequent to infection - Biomarkers of sepsis-induced immunosuppression through genome, transcriptome, metabolome, microbiome and cell population analysis - Cost-effectiveness analysis of clarithromycin against placebo in the study population |
| **Sample size calculation** | This is done for the primary study endpoint. We are planning a study of independent cases and controls with 1 control per case. Prior data indicate that 28-day mortality among placebo-treated patients with sepsis and respiratory dysfunction was 55% and that this was decreased to 30% in patients receiving clarithromycin. We need to study 55 subjects into each group to be able to reject the null hypothesis that the failure rates for both groups are equal with probability (power) 0.8. The Type I error probability associated with this test of this null hypothesis is 0.10. We will use a continuity-corrected chi-squared statistic or Fisher’s exact test to evaluate this null hypothesis. |

**INTRODUCTION**

Sepsis is a condition with actually rising incidence, estimated around 19 cases per 1000 hospitalizations per year in academic hospitals in USA^1^ and similar trends in Europe^2^. It is associated with unacceptably high early (in-hospital) mortality of 40-50%^3^. Current guidelines promote best practice by early recognition and management with timely antibiotic administration, fluids, vasopressors and early identification/ control of infection source^4^. However, in spite of adherence to more intensive and costly protocols of early goal-directed therapy, no further decrease in mortality is achieved^5^. Syndromic approaches on sepsis and therapies targeting immune modulation are under evaluation and failures may partly be due to incomplete understanding of underlying pathophysiological mechanisms and immunological phases (pro and anti-inflammatory) of sepsis^6^.

Macrolides, such as clarithromycin or azithromycin have been shown beneficial in reducing COPD exacerbations (OR 0.55; 95% CI 0.39-0.77; p<0.001) partly through anti-inflammatory properties^7^. Besides, when added to a beta-lactam regimen in community-acquired pneumonia, macrolide therapy was associated with reduced mortality risk (OR 0.67; 95% CI 0.61-0.73; p<0.001) in a recent meta-analysis of observational studies including 42942 patients^8^. An immunomodulatory effect of macrolides, beyond their antimicrobial action may explain these findings.

Clarithromycin as adjunctive treatment to standard antibiotic therapy has been used by our group in two previous large-scale multi-center RCTs conducted in Greece after approval from the Greek regulatory authorities. The first RCT studied 200 patients with sepsis due to VAP and it was conducted between 2004 and 2005 ( [www.clinicaltrials.gov](http://www.clinicaltrials.gov) NCT00297674). Identified pathogens mainly included gram-negative bacteria i.e. *Acinetobacter baumanii* and *Pseudomonas aeruginosa* that do not belong to the usual antimicrobial spectrum of macrolides. Although 28-day mortality in both arms was similar, patients assigned to the clarithromycin arm experienced earlier weaning from mechanical ventilation and more rapid resolution of VAP, compared to the placebo arm. In the subgroup of patients with septic shock and multiple organ dysfunction syndrome (MODS), probability of sepsis-related death was significantly lower (OR 3.78 vs 19; *p* = 0.043). Serious adverse events (SAEs) occurred in 3 (3%) clarithromycin-treated patients, with no clear causative link with the study drug in 2 out of 3 cases^9^. Furthermore, hospitalization costs associated with clarithromycin use were significantly reduced (by 7000 euros/ patient that remained alive) compared to the placebo arm^10^.

The second multi-center RCT compared the efficacy of clarithromycin versus placebo added to standard antibiotic therapy in patients with gram-negative sepsis caused by acute pyelonephritis, intra-abdominal infections and primary gram-negative bacteremia; 600 patients were included between 2007 and 2011 ([www.clincialtrials.gov](http://www.clincialtrials.gov) NCT01223690). Overall mortality at 28 days in both arms did not differ, but probability of death due to septic shock and MODS was shown once more lower in the clarithromycin-treated group (OR 3.58 vs 6.21; p = 0.036). Interestingly, survival benefit from clarithromycin was even greater in those patients suffering from adult respiratory distress syndrome (ARDS). SAEs were described in 2 (0.7%) patients treated with clarithromycin, while its use was associated with saving of a median of 1000 euros/ hospitalized patient^11^. No QT space prolongation or any arrhythmia was observed in both aforementioned trials.

Immunomodulatory effects of clarithromycin in sepsis are not yet elucidated. The analysis of circulating monocytes and of circulating cytokines of patients participating in the first RCT showed that treatment with clarithromycin was associated with a decline in IL-10/ TNFα ratio, greater apoptosis of monocytes, enhanced antigen presentation capacity of monocytes, as well as improved capacity of monocytes for cytokine production suggesting an effect consistent with reversal of sepsis-induced immunosuppression^12^. These findings in conjunction with evidence generated from in vitro and animal experiments suggest modulation of the immune response as the mechanism of action of clarithromycin^13, 14^.

In both RCTs, clarithromycin was administered intravenously at a dose of 1g as continuous one-hour intravenous infusion for three or four days. The drug was safe, well tolerated and cost-effective. However, benefit from treatment in both RCTs was shown only after sub-group analysis of the sub-group of patients with respiratory dysfunction and not when analysis comprised the entire study population. As a consequence, in order to consolidate the benefit of clarithromycin among patients with sepsis and respiratory dysfunction, an RCT is needed in a patient population with sepsis and respiratory dysfunction. This RCT should also comprise a study population with infections likely to be caused by Gram-negative bacteria that do not belong to the antimicrobial spectrum of clarithromycin.

**AIM OF THE STUDY**

The study is aiming to prove the efficacy of intravenous treatment of clarithromycin in the reduction of 28-day mortality among patients with sepsis and respiratory dysfunction. Secondary aims are the effect on overall 90-day mortality, sepsis resolution and recurrence, 28-day mortality in the subgroup of patients with septic shock and impact on genomic, transcriptomic, metabolomic, microbiome and cell population profile, especially biomarkers of sepsis-induced immunosuppression. Finally, this study aims to perform a cost-effectiveness analysis of clarithromycin compared to placebo at 90 days.

**STUDY DESIGN**

***Type of study and study sites***

This is a double- blind, randomized, placebo-controlled clinical study that will be conducted in patients admitted in the following departments of Intensive Care Medicine (ICU) and Internal Medicine in Greece and Belgium (see Appendix I):

4^th^ Department of Internal Medicine, ATTIKON University Hospital, Athens, Greece

2^nd^ Department of Critical Care Medicine, ATTIKON University Hospital, Athens, Greece

Intensive Care Unit, THEAGENEION General Hospital, Thessaloniki, Greece

Intensive Care Unit, G. GENNIMATAS General Hospital, Thessaloniki, Greece

Intensive Care Unit, Ο AGHIOS DIMITRIOS General Hospital, Thessaloniki, Greece

Intensive Care Unit, HIPPOKRATION General Hospital, Thessaloniki, Greece

Intensive Care Unit, KORGIALENEIO BENAKEIO General Hospital, Athens, Greece

Intensive Care Unit, LAIKO General Hospital, Athens, Greece

2^nd^ Department of Internal Medicine, SISMANOGLEION General Hospital, Athens, Greece

Department of Intensive Care, ERASME University Hospital, Brussels, Belgium

Department of Intensive Care, Horta Site, BRUGMANN Hospital, Brussels, Belgium

Department of Intensive Care, Brien Site, BRUGMANN Hospital, Brussels, Belgium

Department of Intensive Care, SAINT-PIERRE Hospital, Brussels, Belgium

This is a phase IV RCT and the study drug will be provided by the Sponsor. The study protocol will be submitted for approval to the Institutional Review Board and subsequently to the Regulatory Authorities of each country; these are the National Ethics Committee and the National Organization of Medicine of Greece or the Federal Agency of Drugs and Medicinal Products of Belgium. After study approval and before enrolment of the first patient, the study will be registered at the website [www.clinicaltrials.gov](http://www.clinicaltrials.gov). Patients will be enrolled after written informed consent provided by themselves or by legal representatives (first-degree relatives) in case of patients unable to consent.

***Inclusion criteria***

ALL following criteria should be met for the inclusion of a patient in the study:

- Adult patients (≥18 years)
- Patients of both genders
- Informed consent form signed by patient or by first-degree relative in case of patient unable to consent
- Negative (blood or urinary) pregnancy test for female patients of reproductive age
- Willingness to receive contraception during and seven days after the administration of the study drug.
- Presence of one or more of the following infections: hospital-acquired pneumonia (HAP), health-care associated pneumonia (HCAP), ventilator-associated pneumonia (VAP), primary Gram-negative bacteremia or intra-abdominal infections. Definitions for these infections are given below.
- Presence of sepsis as defined by the Sepsis-3 classification criteria^3^ (see definitions section below)
- Respiratory dysfunction defined as one PaO_2_/FiO_2_ ratio inferior to 200, independently of the PEEP level.
- Total SOFA points for organ dysfunctions other than the respiratory function more than 3

***Exclusion criteria***

Patients who meet ANY of the exclusion criteria below cannot be enrolled in the study:

- Denial for informed consent
- Age inferior to 18 years
- Pregnancy (confirmed by blood or urinary pregnancy test) or lactation for female patients of reproductive age.
- Unwillingness to receive contraception during and seven days after the administration of the study drug.
- HIV infection (with known CD4 cell count ≤ 200/mm^3^)
- Solid organ, or bone marrow transplantation
- Corticosteroid oral or intravenous intake greater than 0.4 mg/kg of equivalent prednisone daily over the last 15 days; anti-cytokine biological agents (e.g. anti-TNFα), anti-lymphocyte immunoglobulins, Mycofenolate Mofetil, Tacrolimus (FK506) and m-TOR inhibitors (any dose of the above within the past 3 months); Chemotherapy within the past 3 months, Leflunomide intake within the past 2 year, Rituximab within the last year; Methotrexate, Azathioprine, Cyclosporine, Cyclophosphamide (any dose of the above within the last 3 months). Splenectomy, known primary immunodeficiencies. Hydroxyurea, anti-Vascular Endothelial Growth Factor (VEGF), anti-Epithelial Growth Factor Receptor (EGFR), anti-Growth Factor Her-2 and Interferon a, b and γ intake are not considered as exclusion criteria.
- Known active neoplasms or other medical conditions unrelated to sepsis (any of the two), that are compromising short-term survival (1 month)
- Neutropenia <1000/mm^3^
- Known allergy to macrolides
- Previous participation in the study
- Administration of a macrolide for the current infectious episode

***Definitions***

Sepsis is defined by the following criteria, based on sepsis-3 classification^3^:

- Total SOFA score of 2 or more points for patients who are admitted with infection at the emergency department (see APPENDIX II) or
- Increase of admission SOFA score by 2 or more points consequent to infection, for patients already hospitalized (see APPENDIX II)

Hospital-acquired pneumonia (HAP) is defined by the presence of a new or progressive radiographic lung infiltrate in a non-intubated patient hospitalized for more than 48 hours who presents with at least two of the following clinical features:

- Core temperature equal or greater than 38°C
- Total white blood cell count more than 12,000/mm^3^
- Rales or bronchial breath sounds on physical examination
- Purulent sputum
- More than 20 breaths/minute
- Serum procalcitonin more than 0.25 ng/ml
- Gram stain of tracheobronchial secretions or bronchoalveolar lavage fluid indicating the predominance of Gram-negative bacilli

Health-care associated pneumonia (HCAP) is defined by the presence of a new or progressive radiographic lung infiltrate in a non-intubated patient who has at least one of the following risk factors for HCAP^15^:

- Hospitalization the last 90 days
- Residency in a long-term care facility
- Under regular hemodialysis

AND who presents with at least two of the following clinical features:

- Core temperature equal or greater than 38°C
- Total white blood cell count more than 12,000/mm^3^
- Rales or bronchial breath sounds on physical examination
- Purulent sputum
- More than 20 breaths/minute
- Serum procalcitonin more than 0.25 ng/ml
- Gram stain of tracheobronchial secretions or bronchoalveolar lavage fluid indicating the predominance of Gram-negative bacilli^15^

Ventilator-associated pneumonia (VAP) is defined by the presence of a new or progressive radiographic lung infiltrate in a patient who is under mechanical ventilation for at least 48 hours AND who presents with at least two of the following clinical features:

- Core temperature equal or greater than 38°C
- Total white blood cell count more than 12,000/mm^3^
- Purulent tracheobronchial secretions
- Serum procalcitonin more than 0.25 ng/ml
- Gram stain of tracheobronchial secretions or bronchoalveolar lavage fluid indicating the predominance of Gram-negative bacilli^15^

Primary Gram-negative bacteremia (BSI) is defined as the isolation of at least one Gram-negative microorganism from a blood culture of a peripheral vein of a patient that is not related to infection of a central line and who presents with ALL the following features^16^:

- Core temperature equal or greater than 38°C or total white blood cell count more than 12,000/mm^3^
- Thorough clinical and radiological investigation has failed to identify the primary infection site

Intra-abdominal infection (IAI) is defined as the presence of ALL the following features^16^:

- Core temperature equal or greater than 38°C or total white blood cell count more than 12,000/mm^3^
- Radiological findings from abdominal ultrasound or abdominal computed tomography or magnetic resonance imaging consistent with one IAI or peri-operative confirmation of an IAI.

***Study drug preparation and administration***

A separate allocation sequence will be generated for each study site, following a 1:1 design. An allocation sequence will be generated as sealed envelope from a statistician with 1:1 randomization per study site. The envelope is unsealed by an unblinded investigator (or the pharmacist), who is preparing the study drug (placebo or active drug). The preparations will be visually similar and allow blinded administration. Clarithromycin is provided in a form of vial with 500mg of amorphous powder. Two vials are dissolved in 10ml of water for injection each. The prepared 20ml solution is then further diluted to a final volume of 250 ml dextrose in water 5% that is directly connected to the infusion device that leading to a catheter already inserted in a central or peripheral vein. Placebo will consist in 20 ml of water for injection, diluted to a final volume of 250 ml dextrose in water 5% that is directly connected to the infusion device leading to a catheter already inserted in a central or peripheral vein. According to the generated allocation sequence, patients of each study site can be randomly assigned to one of the following two groups:

- *Placebo*; patients receive water for injection at a volume of 20ml diluted to a final volume of 250 ml dextrose in water 5%. This is infused once daily within 1 hour for four consecutive days. All patients allocated to the placebo group will also receive standard therapy at the discretion of their attending physicians.
- *Active drug*; patients receive 1g of clarithromycin dissolved into 20 ml water for injection and then diluted to a final volume of 250 ml dextrose in water 5%. This is infused once daily within 1 hour for four consecutive days as previously described^9, 11^. All patients allocated to the active drug group will also receive standard therapy at the discretion of their attending physicians.

***Study visits***

*Screening visit*

When a patient meets ALL inclusion criteria and NONE of the exclusion criteria, he/she can be enrolled in the study. The criteria are judged based on data available from the patient’s file. Female patients of reproductive age should be screened with a urinary pregnancy test. QT prolongation or prior arrhythmia is not considered as an exclusion criterion for the study, ^9,11^ but may be considered in the decision making of the principal investigator. The same patient cannot be enrolled twice in this study.

*Patient follow-up*

Follow-up of every patient will be done daily until day 28 or hospital discharge (whatever comes first).

Visit 1 is on day 1. Procedures of this day include:

- Recording of demographics, medical history, co-morbidities (Charlson Comorbidity Index), ^17^ SOFA score (see APPENDIX II); Acute Physiology and Chronic Health Evaluation (APACHE) II score (see APPENDIX III), available blood cell count, biochemistry, coagulation time, urine output, blood gas analysis, recording of suspected infection site, available radiological findings, relevant for the current infection microbiology and antimicrobial susceptibility testing if available, administered antimicrobials, other administered drugs and need for source infection control either by percutaneous interventions or by any operation.
- Sampling of 35 ml of venous blood; 3ml is collected into one PAXgene tube or a tube with RNA*later*®; 9ml is collected into one pyrogen-free tube; and 24ml is collected into EDTA-coated tubes
- Collection of stool culture or rectal swab
- Administration of the study drug
- Evaluation of potential adverse events

Visit 2 is on day 2. Procedures of this day include:

- Recording of SOFA score (see APPENDIX II); available blood cell count, biochemistry, coagulation times, urine output, blood gas analysis, available radiological findings, relevant for the current infection microbiology and antimicrobial susceptibility testing if available, administered antimicrobials, other administered drugs and need for source infection control either by percutaneous interventions or by any operation.
- Survival status
- Administration of the study drug
- Evaluation of potential adverse events

Visit 3 is on day 3. Procedures of this day include:

- Recording of SOFA score (see APPENDIX II); available blood cell count, biochemistry, coagulation times, urine output, blood gas analysis, available radiological findings, relevant for the current infection microbiology and antimicrobial susceptibility testing if available, administered antimicrobials, other administered drugs and need for source infection control either by percutaneous interventions or by any operation.
- Evaluation of early sepsis response, defined by at least 25% decrease of visit 1 SOFA on visit 3
- Survival status
- Administration of the study drug
- Evaluation of potential adverse events

Visit 4 is on day 4. Procedures of this day include:

- Recording of SOFA score (see APPENDIX II); available blood cell count, biochemistry, coagulation times, urine output, blood gas analysis, recording of suspected or proven infection site, available radiological findings, relevant for the current infection microbiology and antimicrobial susceptibility testing if available, administered antimicrobials, other administered drugs and need for source infection control either by percutaneous interventions or by any operation.
- Survival status
- Administration of the study drug
- Evaluation of potential adverse events

Visit 5 is on day 5. Procedures of this day include:

- Recording of SOFA score (see APPENDIX II); available blood cell count, biochemistry, coagulation times, urine output, blood gas analysis, available radiological findings, relevant microbiology and antimicrobial susceptibility testing if available, administered antimicrobials, other administered drugs and need for source infection control either by percutaneous interventions or by any operation. Given the previous data from phase IV trials, serial ECGs are not required for this protocol.
- Survival status
- Sampling of 35 ml of venous blood; 3ml is collected into one PAXgene tube or a tube with RNA*later*®; 9ml is collected into one pyrogen-free tube; and 24ml is collected into EDTA-coated tubes
- Collection of stool or rectal swab.
- Evaluation of resolution of the infection and potential recurrence of infection according to the judgment of the attending physician
- Evaluation of potential adverse events

Visit 6 is on day 6. Procedures of this day include:

- Recording of SOFA score (see APPENDIX II); available blood cell count, biochemistry, coagulation times, urine output, blood gas analysis, available radiological findings, clinically relevant microbiology and antimicrobial susceptibility testing, administered antimicrobials, other administered drugs and need for source infection control either by percutaneous interventions or by any operation.
- Survival status
- Evaluation of resolution of the infection and potential recurrence of infection according to the judgment of the attending physician
- Evaluation of potential adverse events

Visit 7 is on day 7. Procedures of this day include:

- Recording of SOFA score (see APPENDIX II); available blood cell count, biochemistry, coagulation times, urine output, blood gas analysis, available radiological findings, clinically relevant microbiology and antimicrobial susceptibility testing, administered antimicrobials, other administered drugs and need for source infection control either by percutaneous interventions or by any operation.
- Survival status
- Evaluation of sepsis response, defined by at least 25% decrease of SOFA score of visit 1
- Evaluation of resolution of the infection and potential recurrence of infection according to the judgment of the attending physician
- Evaluation of potential adverse events

Visit 8 is on day 8. Procedures of this day include:

- Recording of SOFA score (see APPENDIX II); available blood cell count, biochemistry, coagulation times, urine output, blood gas analysis, available radiological findings, clinically relevant microbiology and antimicrobial susceptibility testing, administered antimicrobials, other administered drugs and need for source infection control either by percutaneous interventions or by any operation.
- Survival status
- Evaluation of resolution of the infection and potential recurrence of infection according to the judgment of the attending physician
- Evaluation of potential adverse events

Visit 9 is on day 9. Procedures of this day include:

- Recording of SOFA score (see APPENDIX II); available blood cell count, biochemistry, coagulation times, urine output, blood gas analysis, available radiological findings, clinically relevant microbiology and antimicrobial susceptibility testing, administered antimicrobials, other administered drugs and need for source infection control either by percutaneous interventions or by any operation.
- Survival status
- Evaluation of resolution of the infection and potential recurrence of infection according to the judgment of the attending physician
- Evaluation of potential adverse events

Visit 10 is on day 10. Procedures of this day include:

- Recording of SOFA score (see APPENDIX II); available blood cell count, biochemistry, coagulation times, urine output, blood gases, available radiological findings, clinically relevant microbiology and antimicrobial susceptibility testing, administered antimicrobials, other administered drugs and need for source infection control either by percutaneous interventions or by any operation.
- Survival status
- Evaluation of resolution of the infection and potential recurrence of infection according to the attending physician
- Sampling of 33 ml of venous blood; 9ml is collected into one pyrogen-free tube; and 24ml is collected into EDTA-coated tubes
- Evaluation of potential adverse events

Visits 11-28 are on days 11-28 respectively. They include

- Recording of SOFA score if available
- Administered antimicrobials, other administered drugs and need for source infection control either by percutaneous interventions or by any operation.
- Survival status
- Evaluation of resolution of the infection and potential infection recurrence according to the judgment of the attending physician
- Evaluation of potential adverse events

If the patient is discharged before day 28, a phone call will be performed on day 28 to assess survival status, need for new hospitalization or antibiotic therapy and potential adverse events. End-of-life decisions, if any, will be recorded.

Last visit: A phone call follow-up will be done on day 90 to assess survival status. If available, data concerning any new hospitalization, or antibiotic cure and potential adverse events will be collected during that phone call. Moreover, a self or proxy-assessment of health-related quality of life will be performed through the EQ-5D-3L^™^ questionnaire on the same visit (see APPENDIX IV) ^18^.

All information will be recorded on a specific paper Case Report Form (CRF).

The procedure to follow at each study visit is shown in APPENDIX V.

***Laboratory analysis***

Collected samples will be transported to the central lab that is the Laboratory of Immunology and Infectious Diseases of the 4^th^ Department of Internal Medicine, ATTIKON University General Hospital, Athens, Greece. Analysis will be done as follows:

- PAXgene tubes or tubes with RNA*later*® for will be stored in -80°C for full transcriptomics
- EDTA tubes will be used: partly for direct isolation of peripheral blood mononuclear cells and further cytokine stimulation, partly for flow cytometry, and partly for whole blood immune functional assay. The remaining blood in EDTA tubes will be centrifuged and plasma will be stored in -20°C for metabolomics, cytokine analysis with enzyme immunoassay (EIA) and antibiotic concentration measurement with high-performance liquid chromatography (HPLC) in patients receiving meropenem, tigecyclin or colistin; leucocyte buffy coat remaining after plasma aspiration with sterile pipette will be equally stored in -20°C for genome analysis.
- Serum isolated from pyrogen and anticoagulant-free tube after centrifugation will be stored in -20°C to be used for metobolomic analysis and serum markers.
- Stool or rectal swab will be stored in -80°C, to be later processed for 16S rRNA sequencing of the intestinal microbiome.

***Cost analysis***

The cost of hospitalization associated with each intervention will be calculated in two ways, both of them from a National Health Service and personal social services perspective:

1. According to the first way, cost per day will be defined by the sum of multiplications of each counted item or resource (including administered antimicrobials, other drugs, fluids, blood products, any invasive procedure for source control, mechanical ventilation, renal replacement therapy, radiological examinations, insulin administered for standard glycemic control, enteral or parenteral nutrition) with its price in Euros (€) and the addition of the nominal cost of daily stay for the ICU or general ward. The unit price for each counted item will derive from the National Health Service tariff data official pricelist. The following are excluded from the analysis as their price is considered negligible and/ or equally distributed among groups: vitamins, reconstitution/ dilution fluids, electrolytes, catheter insertions, perfusion devices, monitoring devices, consumables such as laboratory testing, cleaning packs, gloves, or masks, echocardiography performed in ICU for monitoring purposes, transport costs at discharge, cost of human resources such as salary of nursing or medical personnel. Counting of the items will be performed from enrollment until discharge or day 28, by investigators completely blind to the allocated treatment.
2. According to the second way, the total cost of the index hospitalization (where enrollment took place) will be extracted from administrative hospital and insurance records, which are based on Diagnosis Related Groups (DRGs), a combination of ICD-10 diagnosis and codes of medical acts. This is completed by any additional cost of medication or medical equipment plus any extra bed-days per medical or surgical ward or ICU stay, for medications and bed-days exceeding the estimated length of stay per DRG.

Direct cost of additional medical care (e.g. new hospitalizations) up to 90 days will be recorded, if available.

***Cost-Effectiveness analysis***

The EuroCoL EQ-5D-3L^™^ questionnaire at 90 days (proxy and telephone version) data will be used to convert patient responses into Quality-Adjusted Life-Years (QALYs), to define patient-level perception of health-related quality of life on a 0,1 scale. Deceased patients will be counted as a 0 on that scale. Quality of life will be compared with the cost of hospitalization.

**STUDY ENDPOINTS**

**Primary study endpoint**

To assess the impact of intravenously administered clarithromycin as adjunctive treatment to standard antibiotic therapy compared to placebo on all-cause 28-day mortality.

**Secondary study endpoints**

To assess the effect of clarithromycin treatment compared to placebo treatment on the following:

- 28-day mortality in the subgroup of patients with septic shock
- All-cause 90-day mortality
- Early sepsis response, defined by an at least 25% decrease of day 1 SOFA score on day 3
- Sepsis resolution; this is defined by an at least 25% decrease of day 1 SOFA score on day 7
- New sepsis episode until day 28. A new sepsis episode is defined as a further increase of SOFA score by at least 2 points consequent to infection, in a patient who has experienced previous sepsis resolution (at least 25% decrease of day 1 SOFA score on day 7)
- Time until new sepsis episode. A new sepsis episode is noted in any patient who experiences any more than 25% decrease of day 1 SOFA score on day 7 and who has further increase of SOFA by at least 2 points, consequent to infection
- Biomarkers of sepsis-induced immunosuppression, through genome, metabolome, transcriptome, microbiome and cell population analysis
- The (primary) health economic outcome is cost per quality-adjusted life-year (QALY) gained for clarithromycin compared with placebo.

**POWER CALCULATION**

This is done for the primary study endpoint. We are planning a study of independent cases and controls with 1 control per case. Prior data indicate that the mortality among placebo-treated patients with sepsis and respiratory dysfunction was 55% and that this was decreased to 30%^11^. We need to study 55 subjects into each group to be able to reject the null hypothesis that the failure rates for both groups are equal with probability (power) 0.8. The Type I error probability associated with this test of this null hypothesis is 0.10. We will use a continuity-corrected chi-squared statistic or Fisher’s exact test to evaluate this null hypothesis.

**STATISTICAL ANALYSIS**

Qualitative endpoints will be analyzed by the Fisher’s exact test or continuity corrected Chi- square test, as appropriate. Logistic regression models will be used to evaluate variables associated with 28-day mortality. The effect on sepsis-induced immunosuppression will be a composite endpoint and it will result from the bioanalysis of gene expression, circulating cytokines and stimulated cytokines of patients. More precisely, bioanalysis of gene expression is anticipated to provide pathways modulated by treatment. These pathways will be validated by measurement of representative cytokines in cell supernatants. Comparisons of over-time changes will be done between the two groups of treatment. A p - value lower than 0.05 will be considered statistically significant, unless stated otherwise.

For the cost analysis, cost of each intervention will be counted and compared between groups, with non-parametric Mann-Whitney test, as previously described^10^. This analysis will be subject to robustness control to assess potential differences between different methods of cost calculation. Cost-effectiveness will be expressed as an incremental cost-to-effectiveness ratio (ICER) of clarithromycin compared with placebo, in a within-trial analysis (90 days). The ICER will be compared to a willingness-to-pay factor, which is either defined by the National Institute for Health and Care Excellence threshold or by per capita Gross Domestic Product. ^19, 20^

To evaluate the variation and significance of the ICER estimate, a bootstrapping method will be used.

**DURATION OF THE STUDY**

The duration of the study is estimated to two years after approval by the Greek and Belgian regulatory authorities.

**ADVERSE EVENTS**

Adverse events (AEs) and Serious Adverse Events (SAEs) will be collected from baseline until the last patient’s last evaluation. An adverse event is any undesirable medical occurrence in a subject receiving a pharmaceutical product and which does not necessarily have a causal link with this treatment. The adverse event may be a sign, a symptom, or an abnormal laboratory finding.

An adverse reaction is any undesirable and unintended reaction due to investigational medicine product administration (or intervention), related with any dose administrated. If an adverse event/ reaction meets any of the following criteria, it is considered as a *Serious Adverse Event/ Reaction* (SAE):

- Death
- Life-threatening situation The subject was at risk of death at the time of the adverse event/ experience. It does not refer to the hypothetical risk of death if the AE/ adverse reaction were more severe or were to progress.
- Hospitalization or prolongation of existing hospitalization
- Persistent or significant disability/ incapacity Any AE having an outcome that is associated with a substantial disruption of the ability to carry out normal life functions, including the ability to work. This is not intended to include transient interruption of daily activities.
- Congenital anomaly/ birth defects Any structural abnormality in subject’s offspring that occurs after intrauterine exposure to treatment.
- Important medical events/ experiences that may not result in death, be life-threatening, or require hospitalization, may be considered as SAE when, based upon appropriate medical judgment, they may jeopardize the subject and may require medical or surgical intervention to prevent one of the outcomes listed above, i.e. death, a life-threatening adverse event/experience, inpatient hospitalization or prolongation of existing hospitalization, a persistent or significant disability/ incapacity, or a congenital abnormality/ birth defect. Examples of such medical events/ experiences include allergic bronchospasm requiring intensive treatment in an emergency room or at home, blood dyscrasias or convulsions that do not result in inpatient hospitalization, or the development of drug dependency or drug abuse.
- Spontaneous and elective abortions experienced by study subject

*A non-serious adverse event* is one that does not meet the previous definition of a SAE. The severity of the non-serious adverse events will be graded using the following definitions:

- *Mild* - the adverse event/ reaction is transient and well tolerated by the patient
- *Moderate* – The adverse event/ reaction causes discomfort and affects the usual activities of the patient.
- *Severe* – The adverse event/ reaction affects the usual activities of the patient to an important degree and may cause disability or be life-threatening.

*Relationship with the drug*

The time relationship is established if the AE occurs during therapeutic treatment and until 5 half-lives after treatment discontinuation. The investigator will use the following definitions to assess the relationship of the adverse events with the study drug:

- Probably Related: The adverse event has a strong time relationship with the drug or relapses if re-induced, and another etiology is improbable or clearly less probable.
- Possibly Related: The adverse event has a strong time relationship to the drug and an alternative etiology is as probable or less probable.
- Probably not Related: The adverse event has a slight or no time relationship to the drug and/or there is a more probable alternative etiology.
- Unrelated: The adverse event is due to an underlying or concomitant disease or to another pharmaceutical product and is not related to the drug (no time relationship and a much more probable alternative etiology).

If an investigator’s opinion of possibly related, probably not related or not related to study drug is given, an alternative etiology must be provided by the investigator. Please note that a severe adverse event/ experience is not necessarily serious, as the term severe is a measure of intensity, while a serious adverse event is determined based on the aforementioned regulatory criteria.

Individual un-blinding thought to be necessary for the management of an adverse event will be documented in the subject CRF.

All Investigators are held to report every adverse event and evaluate the severity and possible causality with the study drug according to aforementioned criteria. All adverse events/ reactions are reported to Sponsor. The sponsor is responsible for the evaluation of all AEs. All Serious Adverse Events/ Serious Adverse Reactions must be reported to the Sponsor within 24 hours after having the information, by completion of the SAE form and fax to Hellenic Institute of Sepsis. Untoward events resulting from sepsis or infection (such as death or non-resolving infection) will not be recorded as AEs or SAEs, since they are study endpoints.

The Sponsor must evaluate whether an adverse event is expected or not. A SAE may qualify for expedited reporting to regulatory authorities if it is determined to be a suspected, unexpected serious adverse reaction (SUSAR- an adverse reaction, the nature or severity of which is not consistent with the applicable product information -e.g. investigator’s brochure for an unauthorised investigational product or summary of product characteristics for an authorised product). The Sponsor is responsible for submitting expedited safety reports to the appropriate regulatory authority and ethics committee for all confirmed SUSARs. In the case of a fatal or life-threatening SUSAR, the Sponsor will notify the regulatory authorities and the ethics committee as soon as possible but in no case later than 7 calendar days after the Sponsor’s initial receipt of the information. For a non-life-threatening SUSAR, the report will be submitted no later than 15 days after the Sponsor is made aware of the event.

The Sponsor has the obligation to submit annually a drug safety updated report (DSUR) according to global experience to the appropriate regulatory authorities. The electronic submission to Eudravigilance will be performed through the Organization ID: HISS.

The above pharmacovigilance procedures will be performed on behalf of the Sponsor (Hellenic Institute for the Study of Sepsis) by the Consultant Company «SUSTCHEM Engineering P.Braimiotis-P. Scarlatos LTD», 144 3^rd^ Septemvriou str, 11251, Αthens, and the Qualified Person for Pharmacovigilance (QPPV) will be Mrs Areti Voulomenou. (contact details in Appendix I).

**QUALITY CONTROL AND ASSURANCE**

Quality control and assurance checks are performed by the Sponsor in order to allow periodic review of adequacy of the study activities and practices and allow for revising of those practices, as needed, so the data and process are maintained, the study meets the protocol and procedural requirements, and is reproducible.

Before enrolling any subject in this study, sponsor personnel and the investigator review the protocol, the Investigator’s Brochure, the CRFs and instructions for their completion, the procedure for obtaining informed consent, and the procedure for reporting AEs and SAEs.

A qualified representative of the sponsor monitors the conduct of the study by visiting the site and by conduct of the study by visiting the site and by contacting the site by telephone and e-mail. During these site visits, all source documents are reviewed and information recorded in the CRFs is verified against them.

Beside routine monitoring quality assurance will be documented through independent auditing of the quality control activities and where applicable, by regulatory authorities through inspections.

**ETHICAL CONSIDERATIONS**

Prior to the initiation of this study, the study design will receive ethical, scientific and regulatory review. Investigators will conduct this study in accordance with the principles of the Declaration of Helsinki, Good Clinical Practice, and applicable regulatory requirements.

Regarding informed consent form (ICF) signature, before any procedures specified in the protocol are performed, a subject must:

- Be informed of all pertinent aspects of the study and all elements of informed consent
- Be given time to ask questions and to consider the decision to participate
- Voluntarily agree to participate in the study
- Sign and date the updated and approved by ethics committee and regulatory authorities ICF version.

**PROTOCOL ADHERENCE AND AMENDMENTS**

Investigators ascertain that they will apply due diligence to avoid protocol deviations. All significant protocol deviations will be recorded and reported in the clinical study report (CSR). Any change or addition to the protocol can only be made in a written protocol amendment that must be approved and signed by the sponsor, investigator, ethics committee and, where required, regulatory authorities.

**REFERENCES**

1. Kadri SS, Rhee C, Strich JR, Morales MK, Hohmann S, Menchaca J, et al. Estimating ten-year trends in septic shock incidence and mortality in United States academic medical centers using clinical data*.* *Chest* 2016
2. SepNet Critical Care Trials G. Incidence of severe sepsis and septic shock in German intensive care units: the prospective, multicentre INSEP study*.* *Intensive Care Med* 2016; 42: 1980-9.
3. Singer M, Deutschman CS, Seymour CW, Shankar-Hari M, Annane D, Bauer M, et al. The third international consensus definitions for sepsis and septic shock (Sepsis-3)*.* *JAMA* 2016; 315: 801-10.
4. Dellinger RP, Levy MM, Rhodes A, Annane D, Gerlach H, Opal SM, et al. Surviving Sepsis Campaign Guidelines Committee including The Pediatric S. Surviving Sepsis Campaign: international guidelines for management of severe sepsis and septic shock, 2012*.* *Intensive Care Med* 2013;39:165-228.
5. Mouncey PR, Osborn TM, Power GS, Harrison DA, Sadique MZ, Grieve RD, et al. Trial of early, goal-directed resuscitation for septic shock*.* *N Engl J Med* 2015; 372: 1301-11.
6. Ranieri VM, Thompson BT, Barie PS, Dhainaut JF, Douglas IS, Finfer S, et al. Drotrecogin alfa (activated) in adults with septic shock*.* *N Engl J Med* 2012; 366: 2055-64.
7. Herath SC, Poole P. Prophylactic antibiotic therapy for chronic obstructive pulmonary disease (COPD)*.* *Cochrane Database Syst Rev* 2013: CD009764.
8. Nie W, Li B, Xiu Q. Beta-lactam/macrolide dual therapy versus beta-lactam monotherapy for the treatment of community-acquired pneumonia in adults: a systematic review and meta-analysis*.* *J Antimicrob Chemother* 2014; 69: 1441-6.
9. Giamarellos-Bourboulis EJ, Pechère JC, Routsi C, Plachouras D, Kollias S, Raftogiannis M, et al. Effect of clarithromycin in patients with sepsis and ventilator-associated pneumonia*.* *Clin Infect Dis* 2008; 46: 1157-64.
10. Tsaganos T, Raftogiannis M, Pratikaki M, Christodoulou S, Kotanidou A, Papadomichelakis E, et al. Clarithromycin leads to long-term survival and cost benefit in ventilator-associated pneumonia and sepsis*.* *Antimicrob Agents Chemother* 2016; 60: 3640-6.
11. Giamarellos-Bourboulis EJ, Mylona V, Antonopoulou A, Tsangaris I, Koutelidakis I, Marioli A, et al. Effect of clarithromycin in patients with suspected Gram-negative sepsis: results of a randomized controlled trial*.* *J Antimicrob Chemother* 2014; 69: 1111-8.
12. Spyridaki A, Raftogiannis M, Antonopoulou A, Tsaganos T, Routsi C, Baziaka F, et al. Effect of clarithromycin in inflammatory markers of patients with ventilator-associated pneumonia and sepsis caused by Gram-negative bacteria: results from a randomized clinical study*.* *Antimicrob Agents Chemother* 2012; 56: 3819-25.
13. Schultz MJ, Speelman P, Hack CE, Buurman WA, van Deventer SJ, van der Poll T. Intravenous infusion of erythromycin inhibits CXC chemokine production, but augments neutrophil degranulation in whole blood stimulated with Streptococcus pneumoniae*.* *J Antimicrob Chemother* 2000; 46: 235-40.
14. Kanoh S, Rubin BK. Mechanisms of action and clinical application of macrolides as immunomodulatory medications*.* *Clin Microbiol Rev* 2010; 23: 590-615.
15. Kalil AC, Metersky ML, Klompas M, Muscedere J, Sweeney DA, Palmer LB, et al. Management of adults with hospital-acquired and ventilator-associated pneumonia: 2016 clinical practice guidelines by the Infectious Diseases Society of America and the American Thoracic Society*.* *Clin Infect Dis* 2016; 63: e61-e111.
16. Calandra T, Cohen J. The international sepsis forum consensus conference on definitions of infection in the intensive care unit*.* *Crit Care Med* 2005; 33: 1538-48.
17. Charslon ME, Pompei P, Ales KL, MacKenzie CR. A new method of classifying prognostic comorbidity in longitudinal studies: development and validation. *J Chronic Dis* 1987; 40: 373-383
18. Brooks R. EuroQol: the current state of play. Health Policy 1996; 37: 53-72
19. <https://www.nice.org.uk/process/pmg6/chapter/assessing-cost-effectiveness>
20. Marseille E, Larson B, Kazi DS, Kahn JG, Rosen S. Thresholds for the cost-effectiveness of interventions: alternative approaches. *Bull World Health Organ* 2015; 93: 118-124

**APPENDIX I** Participating Sites

4^th^ Department of Internal Medicine, ATTIKON University Hospital, Athens, Greece

2^nd^ Department of Critical Care Medicine, ATTIKON University Hospital, Athens, Greece

Intensive Care Unit, THEAGENEION General Hospital, Thessaloniki, Greece

Intensive Care Unit, G. GENNIMATAS General Hospital, Thessaloniki, Greece

Intensive Care Unit, AGHIOS DIMITRIOS General Hospital, Thessaloniki, Greece

Intensive Care Unit, HIPPOKRATION General Hospital, Thessaloniki, Greece

Intensive Care Unit, KORGIALENEIO BENAKEIO General Hospital, Athens, Greece

Intensive Care Unit, LAIKO General Hospital, Athens, Greece

2^nd^ Department of Internal Medicine, SISMANOGLEION General Hospital, Athens, Greece

Department of Intensive Care, ERASME University Hospital, Brussels, Belgium

Department of Intensive Care, Horta Site, BRUGMANN Hospital, Brussels, Belgium

Department of Intensive Care, Brien Site, BRUGMANN Hospital, Brussels, Belgium

Department of Intensive Care, SAINT-PIERRE Hospital, Brussels, Belgium

Monitor of the study as assigned by the Sponsor is:

For Greece: Mrs Kotsaki Antigoni, MD, PhD

e-mail: antigonebut@yahoo.com, tel. number: +30 694 6637164, 21058312562

For Belgium: Mrs Kyriazopoulou Evdoxia, MD, PhD(c)

e-mail: [ekyri@med.uoa.gr](mailto:ekyri@med.uoa.gr), tel. number: +30 694 7415205‬

QPPV of the study as assigned by the Sponsor is Mrs Areti Voulomenou, MEng, MSc

e-mail: voulomenou@suschem.gr, tel. number: +30 2108252 510

**APPENDIX II** The SOFA score^3^

| **Variable** | **0 points** | **1 point** | **2 points** | **3 points** | **4 points** |
| --- | --- | --- | --- | --- | --- |
| PaO_2_/FiO_2_(mmHg) | ≥400 | <400 | <300 | <200 | <100 |
| Platelets (per mm^3^) | ≥150 | <150 | <100 | <50 | <20 |
| Hypotension | MAP≥ 70 mmHg | MAP<70 mmHg | Dobutamine whatever dose | Adrenaline ≤0.1* or  Noradrenaline≤ 0.1* | Adrenaline>0.1* or  Noradrenaline >0.1* |
| Glasgow Coma Scale | 15 | 13-14 | 10-12 | 6-9 | <6 |
| Bilirubin (mg/dl) | <1.2 | 1.2-1.9 | 2.0-5.9 | 6.0-11.9 | ≥12 |
| Creatinine (mg/dl) or Urine output | <1.2 | 1.2-1.9 | 2.0-3.4 | 35-4.9 or <500ml/day | ≥5.0 or  <200ml/day |

*μg/kg/min

Each variable is scored between 0 and 4. The SOFA score is the sum of the score of each variable

**APPENDIX III Calculation of the APACHE (acute physiology and chronic health evaluation) II score.**

| **PARAMETER** | **VALUES ABOVE NORMAL** | | | | **NORMAL** | **VALUES BELOW NORMAL** | | | |
| --- | --- | --- | --- | --- | --- | --- | --- | --- | --- |
|  | +4 | +3 | +2 | +1 | 0 | +1 | +2 | +3 | +4 |
| 1. Rectal temperature (^o^C) | >41 | 39-40.9 |  | 38. 5-38. 9 | 36-38.4 | 34-35.9 | 32-33.9 | 30-31.9 | <29.9 |
| 2. Mean arterial pressure (mm Hg) | >160 | 130-159 | 110-129 |  | 70-109 |  | 50-69 |  | <49 |
| 3. Heart ventricular rate | >180 | 140-179 | 110-139 |  | 70-109 |  | 55-69 | 40-54 | <39 |
| 4. Respiratory rate (mechanical or no ventilation) | >50 | 35-49 |  | 25-34 | 12-24 | 10-11 | 6-9 |  | <5 |
| 5. Oxygenation: AaDO_2_ or PaO_2_ (mmHg) |  |  |  |  |  |  |  |  |  |
| α) FiO_2_>0. 5: calculate AaDO_2_ | >500 | 350-499 | 200-349 |  | <200 |  |  |  |  |
| β) FiO_2_<0. 5: only PaO_2_ |  |  |  |  | PaO_2_>70 | PaO_2_ 61-70 |  | PaO_2_ 55-60 | PaO_2_ <55 |
| 6. Arterial pH | >7.7 | 7.6-7.69 |  | 7.5-7.59 | 7.33-7.49 |  | 7.25-7.32 | 7.15-7.24 | <7. 15 |
| 7. Serum sodium (mMol/L) | >180 | 160-179 | 155-159 | 150-154 | 130-149 |  | 120-129 | 110-119 | <110 |
| 8. Serum potassium (mMol/L) | >7 | 6-6.9 |  | 5.6-5.9 | 3.5-5.4 | 3-3.4 | 2.5-2.9 |  | <2.5 |
| 9. Serum creatinine (mg/dL)  (x 2 in case of chronic renal failure) | >3.5 | 2-3.4 | 1. 5-1.9 |  | 0. 6-1.4 |  | <0.6 |  |  |
| 10. Hematocrit (%) | 60 |  | 50-59.9 | 46-49.9 | 30-45.9 |  | 20-29.9 |  | <20 |
| 11. White blood cells (1000/mm^3^) | >40 |  | 20-399 | 15-19.9 | 3-14.9 |  | 1-2.9 |  | <1 |
| 12. Glasgow Coma Score GCS (Scoring = 15 - GCS) | 15 – GCS = | | | | | | | | |
| **Total Acute Physiology Score (APS)** | Addition of scores for parameters 1-12 = | | | | | | | | |
| HCO_3_^-^serum (venous blood – mMol/L) | 52 | 41-51.9 |  | 32-40.9 | 22-31.9 |  | 18-21.9 | 15-17.9 |  |
| To be used only if absent arterial gas |  |  |  |  |  |  |  |  |  |

| **Age** | **Score** | **Age** | **Score** | **Age** | **Score** | **Age** | **Score** | **Age** | **Score** |
| --- | --- | --- | --- | --- | --- | --- | --- | --- | --- |
| <44 | 0 | 44-54 | 2 | 55-64 | 3 | 65-74 | 5 | >75 | 6 |

**Chronic disease score**

If the patient has history of severe organ insufficiency or is immunodeficient, scoring is done as follows:

a. No surgery or emergency surgery: + 5 points

b. Post-operative patient after programmed surgery: + 2 points

**TOTAL APACHE II SCORE: ΑPS + AGE + CHRONIC DISEASE SCORE**

**APPENDIX IV** EQ-5D-3L (UK English version)

By placing a tick in one box in each group below, please indicate which statements best describe your own health state today.

**Mobility**

I have no problems in walking about ❑

I have some problems in walking about ❑

I am confined to bed ❑

**Self-Care**

I have no problems with self-care ❑

I have some problems washing or dressing myself ❑

I am unable to wash or dress myself ❑

**Usual Activities** (*e.g. work, study, housework, family or leisure activities)*

I have no problems with performing my usual activities ❑

I have some problems with performing my usual activities ❑

I am unable to perform my usual activities ❑

**Pain/Discomfort**

I have no pain or discomfort ❑

I have moderate pain or discomfort ❑

I have extreme pain or discomfort ❑

**Anxiety/Depression**

I am not anxious or depressed ❑

I am moderately anxious or depressed ❑

I am extremely anxious or depressed ❑

To help people say how good or bad a health state is, we have drawn a scale (rather like a thermometer) on which the best state you can imagine is marked **100** and the worst state you can imagine is marked **0**.

We would like you to indicate on this scale how good or bad your own health is today, in your opinion. Please do this by drawing a line from the box below to whichever point on the scale indicates how good or bad your health state is today.

Best imaginable health state

9 0

8 0

7 0

6 0

5 0

4 0

3 0

2 0

1 0

100

0

Your own health state today

Worst imaginable

health state

**APPENDIX V** Procedures on each study visit (until day 28 or until hospital discharge)

|  | **Study visits** | | | | | | | | | | | | | | | | | | | | | | | | | | | | |  |
| --- | --- | --- | --- | --- | --- | --- | --- | --- | --- | --- | --- | --- | --- | --- | --- | --- | --- | --- | --- | --- | --- | --- | --- | --- | --- | --- | --- | --- | --- | --- |
|  | **1** | **2** | **3** | **4** | **5** | **6** | **7** | **8** | **9** | **10** | **11** | **12** | **13** | **14** | **15** | **16** | **17** | **18** | **19** | **20** | **21** | **22** | **23** | **24** | **25** | **26** | **27** | **28** | **Last** | |
| Study drug | x | x | x | x |  |  |  |  |  |  |  |  |  |  |  |  |  |  |  |  |  |  |  |  |  |  |  |  |  | |
| SOFA score* | x | x | x | x | x | x | x | x | x | x | x | x | x | x | x | x | x | x | x | x | x | x | x | x | x | x | x | x |  | |
| Survival |  | x | x | x | x | x | x | x | x | x | x | x | x | x | x | x | x | x | x | x | x | x | x | x | x | x | x | x | x | |
| CCI | x |  |  |  |  |  |  |  |  |  |  |  |  |  |  |  |  |  |  |  |  |  |  |  |  |  |  |  |  | |
| APACHE score | x |  |  |  |  |  |  |  |  |  |  |  |  |  |  |  |  |  |  |  |  |  |  |  |  |  |  |  |  | |
| Vital signs** | x | x | x | x | x | x | x | x | x | x | x | x | x | x | x | x | x | x | x | x | x | x | x | x | x | x | x | x |  | |
| Concomitant drugs/ procedures | x | x | x | x | x | x | x | x | x | x | x | x | x | x | x | x | x | x | x | x | x | x | x | x | x | x | x | x |  | |
| Infectious site identification | x |  |  | x |  |  |  |  |  |  |  |  |  |  |  |  |  |  |  |  |  |  |  |  |  |  |  |  |  | |
| Resolution/ recurrence of infection** |  |  |  |  |  |  | x | x | x | x | x | x | x | x | x | x | x | x | x | x | x | x | x | x | x | x | x | x |  | |
| AE monitoring | x | x | x | x | x | x | x | x | x | x |  |  |  |  |  |  |  |  |  |  |  |  |  |  |  |  |  |  |  | |
| Microbiology** | x | x | x | x | x | x | x | x | x | x | x | x | x | x | x | x | x | x | x | x | x | x | x | x | x | x | x | x |  | |
| Pregnancy test*** | x |  |  |  |  |  |  |  |  |  |  |  |  |  |  |  |  |  |  |  |  |  |  |  |  |  |  |  |  | |
| PAX Gene | x |  |  |  | x |  |  |  |  |  |  |  |  |  |  |  |  |  |  |  |  |  |  |  |  |  |  |  |  | |
| Serum metabolomics | x |  |  |  | x |  |  |  |  | x |  |  |  |  |  |  |  |  |  |  |  |  |  |  |  |  |  |  |  | |
| PBMCs isolation | x |  |  |  | x |  |  |  |  | x |  |  |  |  |  |  |  |  |  |  |  |  |  |  |  |  |  |  |  | |
| Stool sample | x |  |  |  | x |  |  |  |  |  |  |  |  |  |  |  |  |  |  |  |  |  |  |  |  |  |  |  |  | |

- * SOFA score: if available according to usual clinical practice. On days where SOFA components are not available (e.g. no blood analysis programed by the physician or discharged patient), the last known value will be recorded.
- ** If available and justified by clinical context ***For female patients of reproductive age

# 3. Details of amendments to the original protocol

| **Previous and new wording in track change modus** | **New wording** | **Comments/explanation/reasons for substantial amendment** |
| --- | --- | --- |
| **Protocol**   - Section “Aim of the Study”, page 10 of previous version of protocol, was formulated as following: “Secondary aims are the effect on overall 90-day mortality, sepsis resolution and recurrence, 28-day mortality in the subgroup of patients with septic shock and, finally, impact on genomic, metabolomics and microbiome profile, especially biomarkers of sepsis-induced immunosuppression.” - Section “Study Design”, page 10 of previous version of protocol was formulated as following: “This is a double- blind, randomized, placebo-controlled clinical study that will be conducted in patients admitted in departments of Intensive Care Medicine (ICU) and Internal Medicine in Greece and Belgium” - Section “Exclusion criteria”, page 11 of previous version of protocol, was formulated as following: “Corticosteroid oral or intravenous intake greater than 0.4 mg/kg of equivalent prednisone daily over the last 15 days” and later:   “Known active neoplasms compromising short-term survival (1 month)”   - Section “Definitions”, page 11of previous version of protocol, was formulated as following: “Radiological findings from abdominal ultrasound or abdominal computed tomography consistent with one IAI.” - Section “Study visits”, paragraphs “Visits 11-28” and “Last Visit”, page18-19 of previous version of protocol, were formulated as following:   “Visits 11-28 are on days 11-28 respectively. They include  -Recording of SOFA score if available  -Administered antimicrobials, other administered drugs and need for source infection control either by percutaneous interventions or by any operation.  -Survival status  -Evaluation of resolution of the infection and potential infection recurrence according to the judgment of the attending physician  -Evaluation of potential adverse events  Last visit: A phone call follow-up will be done on day 90 to assess survival status.”   - Section “Laboratory analysis”, page 19 of previous version of the protocol, refers to the following:   -“PAXgene tubes or tubes with RNAlater® for full transcriptomics  -EDTA tubes for isolation of peripheral blood mononuclear cells and further cytokine stimulation, as well as flow cytometry.  -Serum for metobolomic analysis and serum markers”   - Section “Cost analysis”, as well as section “Cost-effectiveness” analysis, did not figure on the previous version of the protocol - Section “Secondary study endpoints”, page 20 of the previous version of the protocol, the last bullet refers to “[the effect of clarithromycin on] real cost at hospital discharge”. - Section “Statistical analysis”, page 20 of the previous version of the protocol, an alinea is added - Section “Adverse events”, page 20 of the previous version of the protocol has been fully replaced. The original form was as following:   “Adverse events (AEs) and Serious Adverse Events (SAEs) will be collected from baseline until the last patient’s last evaluation. An adverse event is any undesirable medical occurrence in a subject receiving a pharmaceutical product and does not necessarily have a causal link with this treatment. The time relationship is established if the AE occurs during therapeutic treatment and until 5 half-lives after treatment discontinuation. The adverse event may be a sign, a symptom, or an abnormal laboratory finding.  Serious adverse events must be reported to the Greek and Belgian agencies of pharmacovigilance for marketed products (EOF and AFMPS- within 7 days for life-threatening situations and within 15 days for serious adverse events of occurrence. The Principal Investigators are held to report SAEs to the sponsor within 24h after having the information.  If an adverse event meets any of the following criteria, it is considered as a Serious Adverse Event (SAE):  -Life-threatening situation The subject was at risk of death at the time of the adverse event. It does not refer to the hypothetical risk of death if the AE was more severe or had come to progress.  -Hospitalization  -Persistent or significant disability/ incapacity Any AE having an outcome that is associated with a substantial disruption of the ability to carry out normal life functions, including the ability to work. This is not intended to include transient interruption of daily activities.  -Important medical events that may not result in death, be life-threatening, or require hospitalization, may be considered as SAE when, based upon appropriate medical judgment, they may jeopardize the subject and may require medical or surgical intervention to prevent one of the outcomes listed above, i.e. death, a life-threatening adverse event/experience, inpatient hospitalization or prolongation of existing hospitalization, a persistent or significant disability/incapacity, (or a congenital abnormality/birth defect). Examples of such medical events include allergic bronchospasm requiring intensive treatment in an emergency room or at home, blood dyscrasias or convulsions that do not result in inpatient hospitalization, or the development of drug dependency or drug abuse.  -Pregnancy  -Spontaneous and elective abortions experienced by study subject  Since death is a study endpoint, deaths will not be reported as SAEs.  A non-serious adverse event is any untoward medical occurrence in a subject receiving a pharmaceutical product, and that does not necessarily have a causal link with this treatment. A non-serious adverse event is one that does not meet the previous definition of a SAE and must be reported to the EOF and AFMPS according to local laws for marketed products. The severity of the non-serious adverse events will be graded using the following definitions:  -Mild - the adverse event is transient and well tolerated by the patient  -Moderate – The adverse events causes discomfort and affects the usual activities of the patient. -Severe – The adverse events affects the usual activities of the patient to an important degree and may cause disability or be life-threatening.  Relationship with the drug. The investigator will use the following definitions to assess the relationship of the adverse events with the study drug:  -Probably Related: The adverse event has a strong time relationship with the drug or relapses if re-induced, and another aetiology is improbable or clearly less probable.  -Possibly Related: The adverse event has a strong time relationship to the drug and an alternative aetiology is as probable or less probable.  -Probably not Related: The adverse event has a slight or no time relationship to the drug and/or there is a more probable alternative aetiology.  -Unrelated: The adverse event is due to an underlying or concomitant disease or to another pharmaceutical product and is not related to the drug (no time relationship and a much more probable alternative aetiology).  If an investigator’s opinion of possibly related, probably not related or not related to study drug is given, an alternate etiology must be provided by the investigator. Please note that a severe adverse event/experience is not necessarily serious, as the term severe is a measure of intensity while a serious adverse event is determined based on the aforementioned regulatory criteria”.   - Section “Quality control and assurance” did not figure on the previous version of the protocol - Section “Ethical Considerations” did not figure on the previous version of the protocol - Section “Protocol adherence and amendments” - Appendix I “The SOFA score”, page 26 of the previous version of the protocol was replaced and has been moved to Appendix II. - Appendix II “APACHE II score”, page 27 of the previous version of the protocol has been moved to Appendix III - Appendix III “Procedures on each study visit”, page 28 of the previous version of the protocol has been moved to Appendix V - Appendix IV did not figure on the previous version of the protocol - Appendix V did not figure on the previous version of the protocol | - This phrase is formulated as following: “Secondary aims are the effect on overall 90-day mortality, sepsis resolution and recurrence, 28-day mortality in the subgroup of patients with septic shock and impact on genomic, transcriptomic, metabolomic, microbiome and cell population profile, especially biomarkers of sepsis-induced immunosuppression”. The following phrase is added: “Finally, this study aims to perform a cost-effectiveness analysis of clarithromycin compared to placebo at 90 days.” - This phrase is formulated as following: “This is a double- blind, randomized, placebo-controlled clinical study that will be conducted in patients admitted in the following departments of Intensive Care Medicine (ICU) and Internal Medicine in Greece and Belgium (see Appendix I):   - 4^th^ Department of Internal Medicine, ATTIKON University Hospital, Athens, Greece  - 2^nd^ Department of Critical Care Medicine, ATTIKON University Hospital, Athens, Greece  - Intensive Care Unit, THEAGENEION General Hospital, Thessaloniki, Greece  - Intensive Care Unit, G. GENNIMATAS General Hospital, Thessaloniki, Greece  - Intensive Care Unit, Ο AGHIOS DIMITRIOS General Hospital, Thessaloniki, Greece  - Intensive Care Unit, HIPPOKRATION General Hospital, Thessaloniki, Greece  - Intensive Care Unit, KORGIALENEIO BENAKEIO General Hospital, Athens, Greece  - Intensive Care Unit, LAIKO General Hospital, Athens, Greece  - 2^nd^ Department of Internal Medicine, SISMANOGLEION General Hospital, Athens, Greece  - Department of Intensive Care, ERASME University Hospital, Brussels, Belgium  - Department of Intensive Care, Horta Site, BRUGMANN Hospital, Brussels, Belgium  - Department of Intensive Care, Brien Site, BRUGMANN Hospital, Brussels, Belgium  - Department of Intensive Care, SAINT-PIERRE Hospital, Brussels, Belgium   - At the end of the phrase, is added: “anti-cytokine biological agents (e.g. anti-TNFα), anti-lymphocyte immunoglobulins, Mycofenolate Mofetil, Tacrolimus (FK506) and m-TOR inhibitors (any dose of the above within the past 3 months), Chemotherapy within the past 3 months, Leflunomide intake within the past 2 years, Rituximab within the last year; Methotrexate, Azathioprine, Cyclosporine, Cyclophosphamide (any dose of the above within the last 3 months), splenectomy, known primary immunodeficiencies. Hydroxyurea, anti-Vascular Endothelial Growth Factor (VEGF), anti-Epithelial Growth Factor Receptor (EGFR), anti-Growth Factor Her-2 and Interferon a, b and γ intake are not considered as exclusion criteria”. Later, the phrase is reformulated as follows:   “Known active neoplasms or other medical conditions unrelated to sepsis (any of the two), that are compromising short-term survival (1 month)”   - It is modified as following: “Radiological findings from abdominal ultrasound or abdominal computed tomography or magnetic resonance imaging consistent with one IAI or peri-operative confirmation of an IAI. - Under “Visits 11-28” is added:“ If the patient is discharged before day 28, a phone call will be performed on day 28 to assess survival status, need for new hospitalization or antibiotic therapy and potential adverse events. End-of-life decisions, if any, will be recorded”.   Under “Last Visit” is added: “If available, data concerning any new hospitalization, or antibiotic cure and potential adverse events will be collected during that phone call. Moreover, a self or proxy-assessment of health-related quality of life will be performed through the EQ-5D-3L^™^ questionnaire on the same visit.   - The section is modified as following:   -“PAXgene tubes or tubes with RNAlater® for will be stored in -80°C for full transcriptomics  -EDTA tubes will be used: partly for direct isolation of peripheral blood mononuclear cells and further cytokine stimulation, partly for flow cytometry, and partly for whole blood immune functional assay. The remaining blood in EDTA tubes will be centrifuged and plasma will be stored in -20°C for metabolomics, cytokine analysis with enzyme immunoassay (EIA) and antibiotic concentration measurement with high-performance liquid chromatography (HPLC), in patients receiving meropenem, tigecyclin or colistin; leucocyte buffy coat remaining after plasma aspiration with sterile pipette will be equally stored in -20°C for genome analysis.  -Serum isolated from pyrogen and anticoagulant-free tube after centrifugation will be stored in -20°C to be used for metobolomic analysis and serum markers.  -Stool or rectal swab will be stored in -80°C, to be later processed for 16S rRNA sequencing of the intestinal microbiome.   - Under the “Cost analysis” section is added:   “The cost of hospitalization associated with each intervention will be calculated in two ways, both of them from a National Health Service and personal social services perspective:  -According to the first way, cost per day will be defined by the sum of multiplications of each counted item or resource (including administered antimicrobials, other drugs, fluids, blood products, any invasive procedure for source control, mechanical ventilation, renal replacement therapy, radiological examinations, insulin administered for standard glycemic control, enteral or parenteral nutrition) with its price in Euros (€) and the addition of the nominal cost of daily stay for the ICU or general ward. The unit price for each counted item will derive from the National Health Service tariff data official pricelist. The following are excluded from the analysis as their price is considered negligible and/ or equally distributed among groups: vitamins, reconstitution/ dilution fluids, electrolytes, catheter insertions, perfusion devices, monitoring devices, consumables such as laboratory testing, cleaning packs, gloves, or masks, echocardiography performed in ICU for monitoring purposes, transport costs at discharge, cost of human resources such as salary of nursing or medical personnel. Counting of the items will be performed from enrollment until discharge or day 28, by investigators completely blind to the allocated treatment.  -According to the second way, the total cost of the index hospitalization (where enrollment took place) will be extracted from administrative hospital and insurance records, which are based on Diagnosis Related Groups (DRGs), a combination of ICD-10 diagnosis and codes of medical acts. This is completed by any additional cost of medication or medical equipment plus any extra bed-days per medical or surgical ward or ICU stay, for medications and bed-days exceeding the estimated length of stay per DRG.  Direct cost of additional medical care (e.g. new hospitalizations) up to 90 days will be recorded, if available.  Under the “Cost-Effectiveness analysis” section is added:  “The EuroCoL EQ-5D-3L™ questionnaire at 90 days (proxy and telephone version) data will be used to convert patient responses into Quality-Adjusted Life-Years (QALYs), to define patient-level perception of health-related quality of life on a 0,1 scale. Deceased patients will be counted as a 0 on that scale. Quality of life will be compared with the cost of hospitalization”**.**   - The last bullet of this section is modified as following: “The (primary) health economic outcome is cost per quality-adjusted life-year (QALY) gained for clarithromycin compared with placebo”. - The following paragraph is added at the end of the section:   “ This analysis will be subject to robustness control to assess potential differences between different methods of cost calculation. Cost-effectiveness will be expressed as an incremental cost-to-effectiveness ratio (ICER) of clarithromycin compared with placebo, in a within-trial analysis (90 days). The ICER will be compared to a willingness-to-pay factor, which is either defined by the National Institute for Health and Care Excellence threshold or by per capita Gross Domestic Product. To evaluate the variation and significance of the ICER estimate, a bootstrapping method will be used”.   - The section is modified as following:   “Adverse events (AEs) and Serious Adverse Events (SAEs) will be collected from baseline until the last patient’s last evaluation. An adverse event is any undesirable medical occurrence in a subject receiving a pharmaceutical product and which does not necessarily have a causal link with this treatment. The adverse event may be a sign, a symptom, or an abnormal laboratory finding.  An adverse reaction is any undesirable and unintended reaction due to investigational medicine product administration (or intervention), related with any dose administrated. If an adverse event/ reaction meets any of the following criteria, it is considered as a Serious Adverse Event/ Reaction (SAE):  -Death  -Life-threatening situation The subject was at risk of death at the time of the adverse event/ experience. It does not refer to the hypothetical risk of death if the AE/ adverse reaction were more severe or were to progress.  -Hospitalization or prolongation of existing hospitalization  -Persistent or significant disability/ incapacity Any AE having an outcome that is associated with a substantial disruption of the ability to carry out normal life functions, including the ability to work. This is not intended to include transient interruption of daily activities.  -Congenital anomaly/ birth defects Any structural abnormality in subject’s offspring that occurs after intrauterine exposure to treatment.  -Important medical events/ experiences that may not result in death, be life-threatening, or require hospitalization, may be considered as SAE when, based upon appropriate medical judgment, they may jeopardize the subject and may require medical or surgical intervention to prevent one of the outcomes listed above, i.e. death, a life-threatening adverse event/experience, inpatient hospitalization or prolongation of existing hospitalization, a persistent or significant disability/ incapacity, or a congenital abnormality/ birth defect. Examples of such medical events/ experiences include allergic bronchospasm requiring intensive treatment in an emergency room or at home, blood dyscrasias or convulsions that do not result in inpatient hospitalization, or the development of drug dependency or drug abuse.  -Spontaneous and elective abortions experienced by study subject  A non-serious adverse event is one that does not meet the previous definition of a SAE. The severity of the non-serious adverse events will be graded using the following definitions:  -Mild - the adverse event/ reaction is transient and well tolerated by the patient  -Moderate – The adverse event/ reaction causes discomfort and affects the usual activities of the patient.  -Severe – The adverse event/ reaction affects the usual activities of the patient to an important degree and may cause disability or be life-threatening.  Relationship with the drug  The time relationship is established if the AE occurs during therapeutic treatment and until 5 half-lives after treatment discontinuation. The investigator will use the following definitions to assess the relationship of the adverse events with the study drug:  -Probably Related: The adverse event has a strong time relationship with the drug or relapses if re-induced, and another etiology is improbable or clearly less probable.  -Possibly Related: The adverse event has a strong time relationship to the drug and an alternative etiology is as probable or less probable.  -Probably not Related: The adverse event has a slight or no time relationship to the drug and/or there is a more probable alternative etiology.  -Unrelated: The adverse event is due to an underlying or concomitant disease or to another pharmaceutical product and is not related to the drug (no time relationship and a much more probable alternative etiology).  If an investigator’s opinion of possibly related, probably not related or not related to study drug is given, an alternative etiology must be provided by the investigator. Please note that a severe adverse event/ experience is not necessarily serious, as the term severe is a measure of intensity, while a serious adverse event is determined based on the aforementioned regulatory criteria.  Individual un-blinding thought to be necessary for the management of an adverse event will be documented in the subject CRF.  All Investigators are held to report every adverse event and evaluate the severity and possible causality with the study drug according to aforementioned criteria. All adverse events/ reactions are reported to Sponsor. The sponsor is responsible for the evaluation of all AEs. All Serious Adverse Events/ Serious Adverse Reactions must be reported to the Sponsor within 24 hours after having the information, by completion of the SAE form and fax to Hellenic Institute of Sepsis. Untoward events resulting from sepsis or infection (such as death or non-resolving infection) will not be recorded as AEs or SAEs, since they are study endpoints.  The Sponsor must evaluate whether an adverse event is expected or not. A SAE may qualify for expedited reporting to regulatory authorities if it is determined to be a suspected, unexpected serious adverse reaction (SUSAR- an adverse reaction, the nature or severity of which is not consistent with the applicable product information -e.g. investigator’s brochure for an unauthorized investigational product or summary of product characteristics for an authorized product). The Sponsor is responsible for submitting expedited safety reports to the appropriate regulatory authority and ethics committee for all confirmed SUSARs. In the case of a fatal or life-threatening SUSAR, the Sponsor will notify the regulatory authorities and the ethics committee as soon as possible but in no case later than 7 calendar days after the Sponsor’s initial receipt of the information. For a non-life-threatening SUSAR, the report will be submitted no later than 15 days after the Sponsor is made aware of the event.  The Sponsor has the obligation to submit annually a drug safety updated report (DSUR) according to global experience to the appropriate regulatory authorities. The electronic submission to Eudravigilance will be performed through the Organization ID: HISS. The above pharmacovigilance procedures will be performed on behalf of the Sponsor (Hellenic Institute for the Study of Sepsis) by the Consultant Company «SUSTCHEM Engineering P.Braimiotis-P. Scarlatos LTD», 144 3rd Septemvriou str, 11251, Αthens, and the Qualified Person for Pharmacovigilance (QPPV) will be Mrs Areti Voulomenou. (contact details in Appendix I)”.   - This section is formulated as following:   “Quality control and assurance checks are performed by the Sponsor in order to allow periodic review of adequacy of the study activities and practices and allow for revising of those practices, as needed, so the data and process are maintained, the study meets the protocol and procedural requirements, and is reproducible. Before enrolling any subject in this study, sponsor personnel and the investigator review the protocol, the Investigator’s Brochure, the CRFs and instructions for their completion, the procedure for obtaining informed consent, and the procedure for reporting AEs and SAEs. A qualified representative of the sponsor monitors the conduct of the study by visiting the site and by conduct of the study by visiting the site and by contacting the site by telephone and e-mail. During these site visits, all source documents are reviewed and information recorded in the CRFs is verified against them. Beside routine monitoring quality assurance will be documented through independent auditing of the quality control activities and where applicable, by regulatory authorities through inspections”.   - This section is formulated as following:   “Prior to the initiation of this study, the study design will receive ethical, scientific and regulatory review. Investigators will conduct this study in accordance with the principles of the Declaration of Helsinki, Good Clinical Practice, and applicable regulatory requirements.  Regarding informed consent form (ICF) signature, before any procedures specified in the protocol are performed, a subject must:  -Be informed of all pertinent aspects of the study and all elements of informed consent  -Be given time to ask questions and to consider the decision to participate  -Voluntarily agree to participate in the study  -Sign and date the updated and approved by ethics committee and regulatory authorities ICF version”.   - This section is formulated as following:   Investigators ascertain that they will apply due diligence to avoid protocol deviations. All significant protocol deviations will be recorded and reported in the clinical study report (CSR). Any change or addition to the protocol can only be made in a written protocol amendment that must be approved and signed by the sponsor, investigator, ethics committee and, where required, regulatory authorities.   - Appendix I has been replaced by the full descriptive Table of all Study Sites and the contact details of responsible monitors and pharmacovigilance. - Appendix II now contains “The SOFA score” - Appendix III now contains the “APACHE II score” - Appendix IV contains the EQ-5D-3L questionnaire - Appendix V contains the “Procedures on each visit” | Replacement of the simple cost analysis by a full cost-effectiveness analysis as a secondary endpoint  Detailed description of the investigational sites  Better characterization of the immunocompromized patients and the patients with short-term survival, that are to be excluded from the trial in order to fulfil the trial’s objectives  More accurate definition of an intra-abdominal infection  The investigator’s responsibilities of follow-up in case of patients’ discharge before day 28, as well as the follow-up on day 90, are clarified and extended.  A more detailed description of sample analysis and handling is provided. Moreover, the measurement of specific antibiotic concentrations is added.  Thorough description of the cost and cost-effectiveness analysis that was added in the objectives of the trial  Description of the metric of cost-effectiveness analysis, in addition to the previous endpoints  A more detailed description of statistical analysis is added, both on cost and cost-effectiveness comparisons.  Better compliance with Regulatory recommendations  Better compliance with regulatory procedures  Better compliance with regulatory procedures  Better compliance with regulatory recommendations  Due to the previous modification  Due to the previous modification  Measurement of efficacy (health-related quality of life) for the purposes of the cost-effectiveness analysis  Due to the previous modifications |

# 4. Statistical Analysis Plan

The INtravenous CLArithromycin in Sepsis with multiple organ dysfunction Syndrome (INCLASS) study aims to assess the efficacy of clarithromycin as adjunctive treatment in reducing all-cause 28-day mortality among severely ill patients with sepsis. The study also aims to provide insight on the mechanism of action underlying clinical outcomes. The study statistical analysis plan (SAP) was conducted by the protocol authors (E. J. Giamarellos-Bourboulis and E. Karakike), as well as the study statistician M. Kyprianou. B. P. Scicluna provided statistical advice on bioinformatics for RNA sequencing. The SAP was established on 26/06/2019, at the end of enrollment and before the unmasking, the database lock and the end of the follow-up period.

**Study Design**

The INCLASS study is a multicenter, international, placebo-controlled, double-blind, parallel group Randomized Clinical Trial (RCT). It is hypothesized that intravenous clarithromycin, as adjunctive treatment to standard of care for sepsis reduces all-cause 28-day mortality compared to placebo. The study aims to include 110 patients from 13 investigational sites, ICU and non-ICU, in Greece and in Belgium.

**Randomization and bliniding**

Patients are assigned to clarithromycin or placebo, following a random allocation sequence, with a 1:1 design and by block sizes of 10, stratified per study site. The allocation sequence was generated by an independent statistician prior to the study start and delivered within sealed individual envelopes, labeled per study participant code, to each study site. The envelope is unsealed by the study pharmacist (the only unblinded investigator), to prepare the study drug. All other parties involved (investigators, patients, healthcare providers) are blinded to the study arm.

Study intervention consists in either 1gr of clarithromycin or 20 ml of water for injection (placebo), administered once daily as a ready-to use intravenous solution of 250 ml D/W 5%. The intervention is administered for 4 consecutive days, as soon as possible after enrollment. There is no specific time window from enrollment to study drug administration, which is required to be as soon as logistically possible.

**Study Population**

**Inclusion Criteria**

- Adult patients (≥18 years)
- Patients of both genders
- Informed consent form signed by patient or by first-degree relative in case of patient unable to consent
- Negative (blood or urinary) pregnancy test for female patients of reproductive age
- Willingness to receive contraception during and seven days after the administration of the study drug.
- Presence of one or more of the following infections: hospital-acquired pneumonia (HAP), health-care associated pneumonia (HCAP), ventilator-associated pneumonia (VAP), primary Gram-negative bacteremia or intra-abdominal infections. Definitions for these infections are given below.
- Presence of sepsis as defined by the Sepsis-3 classification criteria^3^ (see definitions section below)
- Respiratory dysfunction defined as one PaO_2_/FiO_2_ ratio inferior to 200, independently of the PEEP level.
- Total sequential organ failure assessment (SOFA) score for organ dysfunctions other than respiratory system more than 3

***Exclusion criteria***

Patients who meet ANY of the exclusion criteria below cannot be enrolled in the study:

- Denial for informed consent
- Age inferior to 18 years
- Pregnancy (confirmed by blood or urinary pregnancy test) or lactation for female patients of reproductive age.
- Unwillingness to receive contraception during and seven days after the administration of the study drug.
- HIV infection (with known CD4 cell count ≤ 200/mm^3^)
- Solid organ, or bone marrow transplantation
- Corticosteroid oral or intravenous intake greater than 0.4 mg/kg of equivalent prednisone daily over the last 15 days; anti-cytokine biological agents (e.g. anti-TNFα), anti-lymphocyte immunoglobulins, Mycofenolate Mofetil, Tacrolimus (FK506) and m-TOR inhibitors (any dose of the above within the past 3 months); Chemotherapy within the past 3 months, Leflunomide intake within the past 2 year, Rituximab within the last year; Methotrexate, Azathioprine, Cyclosporine, Cyclophosphamide (any dose of the above within the last 3 months). Splenectomy, known primary immunodeficiencies. Hydroxyurea, anti-Vascular Endothelial Growth Factor (VEGF), anti-Epithelial Growth Factor Receptor (EGFR), anti-Growth Factor Her-2 and Interferon a, b and γ intake are not considered as exclusion criteria.
- Known active neoplasms or other medical conditions unrelated to sepsis (any of the two), that are compromising short-term survival (1 month)
- Neutropenia <1000/mm^3^
- Known allergy to macrolides
- Previous participation in the study
- Administration of a macrolide for the current infectious episode

**Primary study endpoint**

All-cause 28-day mortality.

**Secondary study endpoints**

- 28-day mortality in the subgroup of patients with septic shock
- All-cause 90-day mortality
- Early sepsis response, defined by an at least 25% decrease of day 1 SOFA score on day 3
- Sepsis resolution; this is defined by an at least 25% decrease of day 1 SOFA score on day 7
- New sepsis episode until day 28. A new sepsis episode is defined as a further increase of SOFA score by at least 2 points consequent to infection, in a patient who has experienced previous sepsis resolution (at least 25% decrease of day 1 SOFA score on day 7)
- Time until new sepsis episode. A new sepsis episode is noted in any patient who experiences any more than 25% decrease of day 1 SOFA score on day 7 and who has further increase of SOFA by at least 2 points, consequent to infection
- Biomarkers of host immune response, through genome, metabolome, transcriptome, microbiome and cell population analysis
- The (primary) health economic outcome is cost per quality-adjusted life-year (QALY) gained for clarithromycin compared with placebo.

**Safety outcomes**

- Adverse events (AEs)
- Serious adverse events (SAEs)
- Suspected unexpected serious adverse reactions (SUSARs)

Patients are followed-up daily until day 28 or hospital discharge (whatever comes first). In case of earlier discharge, the primary outcome, is assessed by phone call at day 28. 90-day mortality and quality of life by the EuroQol**^®^** 5-dimension 3-level (EQ-5D-3L) questionnaire (1), in case of discharge, are also assessed by phone call with the patient or their caregiver. Safety outomes are captured daily until day 28 or discharge (whichever comes first) and as patient or provider-reported events until day 90. All other secondary outcomes are assessed until day 28 or discharge.

**Sample size calculation**

This is done for the primary endpoint. Based on previous studies, we anticipated a 25% reduction of mortality among patients with sepsis and respiratory dysfunction, treated with clarithromycin. To detect this difference with a type-I error of 0.10 and a type-II error of 0.8 and anticipating a 10% loss to follow-up, 55 patients need to be enrolled in each study arm.

**Populations**

Analyses will be performed on an intention-to-treat (ITT) basis, including all randomized patients, independently of wheather they received the sudy intervention. The ITT population will enter analysis of the primary and all secondary outcomes. In case of consent withdrawal, all data collected prior to this point will be excluded.

The following subgroups of patients are potentially identified:

- Enrolled inside and outside of the intensive care unit (ICU)
- Enrollement in Greece and in Belgium
- Presence or absence of septic shock
- Presence or absence of acute respiratory dysfunction syndrome (ARDS)
- Extremely high severity, defined as patients within the highest quartile of SOFA score, vs all others.
- Presenting pneumonia, intra-abdominal infection or primary Gram-negative bacteremia
- Presence or absence of a Gram-negative pathogen (among infections with identified pathogen only)
- Adequate vs non-adequate empirical antimicrobial treatment (among infections with identified pathogen only). Empirical antimicrobial treatment (defined as the regimen given within 48 hours from infection onset, when cultures are pending) is considered as adequate when administered at the indicated dose and with penetration to the infected site; provided that the identified pathogen has documented or reasonably suspected susceptibility (e.g. metronidazole for *Clostridioides difficile* infection)
- Per protocol (PP) population, defined by patients receiving the entire intervention (all 4 daily doses)

Subgroup analyses will be performed for mortality outcomes (28-day and 90-day).

**General principles**

- This analysis plan and the primary manuscript will include clinical data up to 90 days, transcriptomic and cell population (by flow cytometry) analyses; the entire cost-effectiveness analysis, cytokine analysis (including serum cytokine concentrations and cytokine production from supernatants of ex-vivo stimulated peripheral blood mononuclear cells-PBMCs) and microbiome analysis will be reported separately.
- Regarding flow cytometry analysis (including cell population and HLA-DR analysis), only samples from patients enrolled in sites proximal to the central lab (Athens) could be included in the current study.
- Regarding trasncriptomic analysis, only a subset of sample sequencing could be covered by the study funding at the time of the present statistical analysis plan. Thus, it was decided to only include in the primary manuscript samples from the subgroup of patients of extremely high severity (within the highest quartile of baseline SOFA score), to demonstrate differences between death and survival. The entire transcriptomic analysis, combined with metabolome, cytokine and microbiome analysis will be reported separately.
- All p-values will be two-sided and the level of significance will be <0.05.
- No interim analysis is planned.
- Regarding clinical data, cell populations and HLA-DR measurements, no adjustment for multiple comparisons is planned and all secondary outcomes will be viewed as exploratory. For transcriptomic data, statistically significant differences will be defined by Benjamini & Hochberg adjusted probabilities < 0.05 and absolute fold expression ≤−1.5 or ≥1.5.
- Analyses will be conducted primarily by the SPSS statistical software, version 24 (IBM) or later; transcriptomic data twill be analysed in the R statistical framework (Version 3.51, R Core Team 2014. R: A language and environment for statistical computing. R Foundation for Statistical Computing, Vienna, Austria)
- Results will be presented by order of importance, starting with the primary outcome first. Due to the nature of the study, additional exploratory analyses will be performed based on the primary and secondary outcomes, in an effort to identify potential host immune pathways modulated by the study drug.

**Descriptives and baseline comparisons**

- Categorical variables will be expressed as counts (and percentages), providing the number of patients or events entered at every step of the analysis.
- The Kolmogorov-Smirnov test for normality will be conducted to the entire population and the arms of study intervention. Continuous variables with normal distribution will be presented with their mean and standard deviation (SD), those with a skewed distribution will be presented with their median and quartiles (Q1-Q3).
- The following variables will be compared among study groups:
  - **Severity and demographics.** Age at inclusion, gender, weight, (enrollment) acute pathophysiology and chronic health evaluation (APACHE) II score, (enrollment) Charlson Comorbitdy Index (CCI), baseline SOFA score (identified as the worst SOFA within any 24h between screening and randomization), sepsis start date, infection start date, time (days) from hosital admission to enrollment, ARDS at enrollment, mechanical ventilation at enrollment, septic shock at enrollment, laboratory data (the most abnormal value within the last 24h prior to enrollment), microbiological data (from infection start).
  - **Concomittant treatment,** including all medication, including inotropic, antimicrobial, fluids and blood products received at enrollment. Medication througout 28 days will be assessed for cost-effectiveness analysis.
  - **Adequacy** of antimicrobial treatment will be defined as susceptibility of the identified pathogen (if any) to the empirical treatment given 48h after sepsis start, with optimal dosing and penetration to infection site.

**Analyses for the primary outcome**

The primary outcome will be assesed on an intention-to-treat (ITT) basis, as a 2x2 contigency table by the Fischer’s exact test, and will be confirmed by logistic regression analysis, adjusted for the following severity variables: Age, gender, CCI, SOFA, APACHE II and adequacy of empirical antimicrobial treatment. In case of high collinearity between the model variables, such as age and APACHE, age and CCI or APACHE and SOFA (assessed by the variance inflation factor-VIF, and correlation), if VIF>2 or Pearson correlation coefficient ≥0.8, then only the variable with the lowest p-value in the univariate regression analysis will be entered in the multivariable model. Multivariable regression analysis will be performed in a forward step-wise manner.

Moreover, the rate of incidence of the primary outcome will be compared between the two arms by the log-rank test and the effect size will be quantified by Cox-regression analysis. Finally, the mean absolute difference in mortality among treatment arms will also be presented, with respective 95% CI and the z-statistic for significance.

- Subgroup analyses, with regards to the primary outcome, are planned according to all the subpopulations identified, as follows: per country; per ward of enrollment (ICU or general ward); per protocol (according to whether the study intervention was complete -all 4 doses received); per presence of septic shock (already present in the protocol); per highest SOFA score (defined as higher or lower than the upper SOFA quartile of the population); per presence of ARDS; per initial infectious site; per adequacy of empirical antimicrobial treatment; and per Gram-negative vs non-Gram negative pathogen, among infections with identified pathogens. Interactions between subgroups and arm of treatment were assessed with Breslow-Day test and confirmed with logistic regression for the primary outcome.

**Analyses for secondary outcomes**

The effect of the study arm on secondary outcomes will be compared with the Pearson chi-square test, or the two-sided Fisher’s exact test for 90-day mortality, early sepsis response and sepsis resolution, as well as new sepsis incidence. Clinical secondary outcomes will be evaluated on an ITT basis.

- Regarding 90-day mortality, the following subgroup analyses are planned (for all subpopulations identified): per country; per ward of enrollment (ICU or general ward); per protocol (according to whether the study intervention was complete -all 4 doses received); per presence of septic shock (already present in the protocol); per highest SOFA score (defined as higher or lower than the upper SOFA quartile of the population); per presence of ARDS; per initial infectious site; per adequacy of empirical antimicrobial treatment; and per Gram-negative vs non-Gram negative pathogen, among infections with identified pathogens. Interactions between subgroups and arm of treatment were assessed with Breslow-Day test and confirmed with logistic regression for the primary outcome.
- The time to new sepsis incidence will be compared only for patients experiencing a new sepsis episode both by the Mann-Whitney test, and as Cox-regression analysis of the treatment effect on the time to new sepsis episode, with death as competing event.
- Cell populations and monocytic HLA-DR variables will be assessed using Student’s t-test (for variables following a normal distribution), Mann-Whitney U (for variables following a skewed distribution), as appropriate. Overtime changes will be assessed by the k-related samples non-parametric Friedman test, separately per study arm, and the mixed ANOVA test with time and study arm as within-subject and between-subject factors respectively.
- Transcriptomic data wil be illustrated by Volcano plots for differential expression of genes among study groups, while gene set enrichement analysis will also be performed to identify pathways of transcriptomic changes among study groups. Any need for additional (post-hoc) analyses will be re-evaluated. Functionality of differentially expressed transcripts will be assessed by the ENCODE project.

Raw sequence data and count data will be uploaded on National Center for Biotechnology Information (NCBI) gene expression omnibus (GEO), to be publicly available.

**Safety outcomes**

The percentage of patients having at least one category of AE, SAE and SUSAR, will be compared by the Fisher’s exact test.

**Missing values**

Missing values will be treated as such, and the number and percentage of patients or events entering each step of the analysis will be provided. The Little’s test will be used to assess whether missing values are missing completely at random.

**Reference:**

1. Rabin R, de Charro F. EQ-5D: a measure of health status from the EuroQol Group. Ann Med. 2001 Jul;33(5):337-43. doi: 10.3109/07853890109002087.
